
JOURNAL INFORMATION
==============================
NLM Title Abbreviation: Crit Care
NLM Journal ID: 9801902

Critical Care

ISSN: 1364-8535
EISSN: 1466-609X
Publisher: BMC

ARTICLE INFORMATION
==============================
PMCID: PMC5496017
DOI: 10.1186/s13054-017-1706-1
Article ID: 1706
Article version: 1
Subjects: Meeting Abstracts

9th International Symposium on Intensive Care and Emergency Medicine for Latin America

São Paulo, Brasil. June 14-17, 2017

Electronic publication date: 2017 Jun 13
Publication date: 2017
Volume: 21
Issue: Suppl 2
Issue sponsor: Publication of this supplement was funded by ISICEM FOR LA
Electronic Location ID: 139
Copyright: © The Author(s). 2017
License: Open AccessThis article is distributed under the terms of the Creative Commons Attribution 4.0 International License (http://creativecommons.org/licenses/by/4.0/), which permits unrestricted use, distribution, and reproduction in any medium, provided you give appropriate credit to the original author(s) and the source, provide a link to the Creative Commons license, and indicate if changes were made. The Creative Commons Public Domain Dedication waiver (http://creativecommons.org/publicdomain/zero/1.0/) applies to the data made available in this article, unless otherwise stated.
License URL: https://creativecommons.org/licenses/by/4.0/

Conference name: 9th International Symposium on Intensive Care and Emergency Medicine for Latin America
Conference Acronym: ISICEM FOR LA 2017
Conference location: São Paulo, Brasil
Conference date: June 14 - 17, 2017
issue-copyright-statement: © The Author(s) 2017

==============================
*Critical Care* 2017, **21**(Suppl 2):0
DOI 10.1186/s13054-017-1706-1

Critical Care

MEETING ABSTRACTS Open Access

### 9th International Symposium on Intensive Care and Emergency Medicine for Latin America

São Paulo, Brasil. June 14-17, 2017

Published: 13 June 2017

### Cardiology

### P01
**Complications associated with the use of temporary pacemakers in hospitalized patients awaiting definitive implant procedure in a public hospital in Sao Paulo**

Andres Eduardo Larrovere Vasquez, Aymée Lustosa Nogueira Torres, Paulo Vinicius Prates Silva, Ana Helena Vicente Andrade, Luciana Gioli-Pereira
HMBM - Hospital Municipal Dr Moyses Deutsch, São Paulo, SP, Brasil
*Critical Care* 2017, **21(Suppl 2):**P01

**Introduction:** Indications for temporary and permanent pacemaker implantation are well established and atrioventricular blocks are the most common causes. Complications could occur, mainly with temporary pacemakers and they can be related to various conditions as the implantation position of the lead, infection and thrombo-embolic events [1,2]. The number of pacemaker implantations in Brazil by the public health system (SUS) is inferior compared to other countries. Besides, the demand is growing and the majority of public hospitals do not realize the procedure [3]. This scenario gets worse with the time to wait for a pacemaker and enhance the complications for the hospitalized patients.
**Objective:** We aim to evaluate clinical complications associated with prolonged hospitalization of patients who are waiting for a permanent pacemaker at a public hospital and the comparison of definitive pacemaker implant costs versus hospitalization required for the procedure.
**Methods:** This is an observational retrospective study that was carried out with medical records of the patients admitted at Dr Moysés Deutsch Municipal Hospital from January 2014 to December 2015 with atrioventricular blocks that requiring a temporary pacemaker. The inclusion criteria were patients aged at least 18 years old and a diagnosis related to atrioventricular blocks. The clinical data were collected in electronic medical records and the outcomes analyzed were all-cause mortality and clinical-surgical complications during hospitalization.
**Results:** Twenty seven patients that implanted a temporary pacemaker were included. The mean length of hospital stay was approximately 20 days. Eighteen (66.6%) patients presented some intercurrence during the hospitalization whose main causes were: worsening renal function (22.2%), decompensated heart failure (18.5%), urinary tract infection (18.5%) and pneumonia (11.1%). There were two (7.4%) deaths likely coronary acute syndrome and one (3.4%) due to sepsis.
**Conclusions:** Preliminary results suggest that there is a relation between hospitalization time and number of complications suffered by the patient. Besides, the complications were more frequent in long stay. The costs involved in the definitive pacemaker implantation procedure and an admission to SUS (public hospitals) will still be analyzed and presented later.

**References**
1. The Task Force on cardiac pacing and resynchronization therapy of the European Society of Cardiology (ESC). Developed in collaboration with the European Heart Rhythm Association (EHRA). 2013 ESC Guidelines on cardiac pacing and cardiac resynchronization therapy. European Heart Journal. 2013; 34: 2281–2329.
2. Pereira WL, Cardoso CC, Fumagalli AR, Carbone Filho F. Complicação rara do marcapasso transvenoso temporário: a formação de loopings e nós. Relampa. 2010; 23(3):134-137.
3. Pachón-Mateos JC et.al. RBM – Registro Brasileiro de Marcapassos, Ressincronizadores e Desfibriladores. Relampa. 2013; 26(1):39-49.

### P02
**Omentopexy as a mechanism of stem cell implantation and revascularization in the ischemic myocardium**

Luiz Fernando Kubrusly[1,2], Yorgos Graça Salles[1,3], Camila Moraes Marques[4,1], Fernando Bermudez Kubrusly[1,2], Angeline Garcez Massignan[3,1], Carolina De Marchi Capeletto[3,1], Luiza Milanesi Abeling[1,3], Caroline Aragão[5,1], Andressa de Souza Bertoldi[3,1], Larissa Maria Vosgerau[3,1], Gabriel Antonio Coltro[3,1]
[1]IDC - Instituto Denton Cooley, Curitiba, PR, Brasil; [2]IVEP - Instituto Vita de Ensino e Pesquisa, Curitiba, PR, Brasil; [3]FEPAR - Faculdade Evangélica do Paraná, Curitiba, PR, Brasil; [4]UFPR - Universidade Federal do Paraná, Curitiba, PR, Brasil; [5]HC- UFPR - Hospital de Clínicas/ UFPR,Curitiba, PR, Brasil
*Critical Care* 2017, **21(Suppl 2):**P02

**Introduction:** Despite improved techniques of myocardial revascularization in the treatment of ischemic diseases, there are patients who can not be benefited by their diffuse involvement of arteries with diameters incompatible with the techniques [1]. The implantation of stem cell at the ischemic myocardial has proved to be able to regenerate myocardium. The omentum is known for applications as highly vascularized graft, full of angiogenic and chemostats factors [2].
**Objective:** This research intends to investigate the efficacy of omentopexy as a indirectly method of revascularization and deliverer of stem cells in the ischemic myocardial.
**Methods:** Myocardial infarction was created in 4 pigs by direct ligation of the 1st and 2nd obtuse marginal branches of the circumflex artery. Lidocaine was administered 0.2% to avoid occurrence of arrhythmias. After 90 minutes of hemodynamic stabilization in 3 animals (Group A), followed by mobilization of the omentum into the mediastinum, the omentum was sutured in the infarcted area. In Group B (1 animal) nothing was done after the infarct. After 30 days of ligation, in both groups, the animals were euthanized. All hearts were removed for histologic evaluation. Nine transversal cuts from the base to the Apex, colored by Hematoxilin-Eosin. It was used CD 34 for expression of stem cells proliferation.
**Results:** Group A (with omentopexy) had progressive fibrosis and thinning of the ventricular wall since the area of the artery ligature until the area treated with the omentum. After this point there was progressive atenuation of ischemic changes up to the Apex where almost normal tissue was found. The CD 34 showed presence of stem cell in the myocardium. In Group B (without omentopexy) there were degrees of adherence, but without development of myocardial vessels. There was evident thinning of the infarcted myocardium.

© The Author(s). 2017 **Open Access** This article is distributed under the terms of the Creative Commons Attribution 4.0 International License (http://creativecommons.org/licenses/by/4.0/), which permits unrestricted use, distribution, and reproduction in any medium, provided you give appropriate credit to the original author(s) and the source, provide a link to the Creative Commons license, and indicate if changes were made. The Creative Commons Public Domain Dedication waiver (http://creativecommons.org/publicdomain/zero/1.0/) applies to the data made available in this article, unless otherwise stated.

**Conclusions:** Omentopexy was able to develop neovascularization in the ischemic myocardial, preserved its thickness and allowed stem cells to implant at the site of omentopexy.

### References

1. Cantero MA, et al. Análise dos resultados imediatos da cirurgia de revascularização do miocárdio com e sem circulação extracorpórea. Rev Bras Cir Cardiovasc.2012;27(1):38-44.
2. Wystrychowski W, et al. Multipotency and cardiomyogenic potential of human adipose-derived stem cells from epicardium, pericardium, and omentum. Stem Cell Research & Therapy.2016;7:84.

### P03

### Anxiety and depressive symptoms in adults and elderly submitted to cardiac surgery

Débora D`Agostini Jorge Lisboa[1], Eliane Lucia Colussi[1], Marlene Doring[1], Gabriela Decol Mendonça[1], Gabriela Colussi[1], Luana Battistella[2], Patricia De Carli Tonial Ghisolfi[1], Julia Mognon[2], Isadora Sisto Rebolho[2], Indaiara Medeiros da Silva[2], Renato Ravizon Lisboa[3]
[1]UPF - Universidade de Passo Fundo (Passo Fundo, RS, Brasil); [2]HCPF - Hospital da Cidade Passo Fundo (Passo Fundo, RS, Brasil); [3]PM - Prefeitura de Marau (Marau, RS, Brasil)
*Critical Care* 2017, **21(Suppl 2):**P03

**Introduction:** The patient undergoing cardiac surgery often experience strong feelings of distress and anxiety befor surgery because it is a highly invasive procedure [1,2]. Anxiety and depression symptoms before surgery can lead to complications after surgery [3].
**Objective:** Identify the presence of symptoms suggestive of depression and anxiety in patients undergoing cardiac surgery
**Methods:** It is an uncontrolled prospective cohort study in two hospitals in Passo Fundo. Data collection occurred in different times, preoperative, postoperative during hospitalization and after three months of surgery, through the Clinical Sociodemographic Questionnaire, Rating Scale Anxiety and Depression level. Data were analyzed using descriptive inferential analysis of the data, at the 0.05 significance level.
**Results:** Observed frequency of symptoms of anxiety in stage I was 38.6% at 18.6% phase II and phase III of 8.6% in patients undergoing cardiac surgery with symptoms suggestive of depression observed a frequency in phase I of 12 stage II and 10.0% in stage III of 7.1%.
**Conclusions:** After analyzing the data, patients have a higher degree of anxiety and depression in the preoperative (phase I), with significant reduction of these symptoms follow up three months after the procedure (phase III) (Table 1).

### References

1. Aikawa, P. et al. Reabilitação cardíaca em pacientes submetidos à cirurgia de revascularização do miocárdio. Rev Bras Med Esporte, v. 20, n. 1, p. 55–58, 2014.
2. Bahramnezhad, F. et al. Quality of Life in Patients Undergoing Percutaneous Transluminal Coronary Angioplasty (PTCA). Global journal of health science, v. 7, n. 5, p 50, jan. 2015.
3. Choinière, M. et al. Prevalence of and risk factors for persistent postoperative nonangial pain after c surgery & #8239;: a 2-year prospective multicentre study. CMAJ, v. 186, n. 7, p. 213–23, 2014. HAD: Escala hospitalar de ansiedade e depressão. Valores expressam frequência absoluta e relativa

**Table 1 (Abstract P03).** Prevalence of anxiety and depression in patients submitted at the study (*n* = 70), Passo Fundo, 2015

| | Preoperative | Postoerative during hospitazation | Postoperative three months after surgery |
|---|---|---|---|
| Variables | n(%) | n(%) | n(%) |
| Anxiety (HAD) - yes | 27 (38,6) | 13 (18,6) | 6 (8,6) |
| Depression (HAD) - yes | 9 (12,9) | 7 (10,0) | 5 (7,1) |

*HAD* Escala hospitalar de ansiedade e depressão
Valores expressam frequência absoluta e relativa

### Epidemiology

### P04

### The effects of weekend admission on the mortality of patients admitted to intensive care units: the role of organizational factors

Fernando Zampieri[1], Thiago Lisboa[2], Fernando Bozza[3], Jorge Salluh[3], Marcio Soares[3]
[1]IP-HCor - Instituto de Pesquisa, HCor-Hospital do Coração, São Paulo, SP, Brasil; [2]SC-Porto Alegre - Santa Casa de Misericórdia, Porto Alegre, RS, Brasil; [3]D´Or IR - D'Or Institute for Research and Education. Rio de Janeiro, RJ, Brasil
*Critical Care* 2017, **21(Suppl 2):**P04

**Introduction:** Weekend admission is considered a risk factor for worse outcomes in critically ill patients, but more robust information on the underlying mechanisms related to intensive care unit (ICU) organization is still lacking.
**Objective:** To assess whether ICU organizational and staffing patterns could explain the association between weekend admission and outcomes in critically ill patients.
**Methods:** Retrospective cohort study using the ORCHESTRA study database [1] comprising 59,614 patients admitted to the participant ICUs during 2013. The association between weekend admission and hospital mortality was assessed using mixed logistic regression adjusting for clinically relevant patient-level (age, severity of organ dysfunctions, comorbidities, performance status; admission type and length of hospital stay before ICU admission) and ICU-level (use of checklists on weekend; 24/7 full-time intensivists; nurse/bed ratio; unit type and number of protocols) characteristics.
**Results:** 41,894 patients (70.3%) were admitted on weekdays and 17,720 patients (29.7%) were admitted on weekends. In univariate analysis, weekend admitted patients had higher ICU (10.9% vs. 9.0%, $p < 0.001$) and hospital (16.5% vs. 13.5%, $p < 0.001$) mortality rates. After regression, weekend admission was not associated with higher hospital mortality (OR 1.05, 95% CI 0.99-1.12, $p = 0.095$). However, a "weekend effect" was still observed in scheduled surgical patients, as well as in ICUs not holding checklists and with a decrease in nurse/bed ratio during the weekends. In addition, units with a lower number of implemented protocols had also higher mortality for patients admitted during the weekends. For unscheduled admissions, no "weekend effect" was observed regardless of ICU´s characteristics.
**Conclusions:** Weekend admissions were associated with higher mortality in certain situations related to potentially modifiable patients' and centers' features, representing an opportunity to improve ICU performance and patients' outcomes.

### Reference

1. Soares M, Bozza FA, Angus DC, et al. Organizational characteristics, outcomes, and resource use in 78 Brazilian intensive care units: the ORCHESTRA study. Intensive Care Med. 2015 Dec;41(12):2149-60.

### P05

### Outcome of oncological patients admitted to ICU for full treatment who were discharged on exclusive palliative care with consensus to no readmission to ICU

Ana Paula Agnolon Praça, Pedro Caruso, Antônio Paulo Nassar Junior
AC Camargo - AC Camargo Cancer Center, São Paulo, SP, Brasil
*Critical Care* 2017, **21(Suppl 2):**P05

**Introduction:** The number of oncological patients admitted to intensive care unit (ICU) is increasing worldwide [1]. However, there have always been discussions about the benefit of ICU for certain groups of patients [2] and concerns with end of life care, because it is usually in the ICU that the transition from full treatment to exclusive palliative care occurs. The decision making process involves patients, relatives, the attending team and is usually a shared and consensual decision [3]. Our hypotheses are that the patients discharged from the ICU on exclusive palliate care would have a high hospital and long-term mortality, would have a small but not neglecting ICU readmission rate despite the consensus to no ICU readmission, and finally some would resume their cancer treatment.

**Objective:** The objectives were to evaluate on patients discharged from ICU on exclusive palliative care: 1. the hospital and long-term mortality; 2. the ICU readmission rate and 3. the resuming of cancer treatment. We believe that our results will help patients, the attending team and relatives to make a more reasoned decision.
**Methods:** This is a retrospective, descriptive study, performed in a medical-surgical ICU of a cancer center, from April 2012 to April 2016 including all patients with solid and hematological tumors, admitted to ICU on full treatment and who were discharged from ICU on exclusive palliative care.
**Results:** 13,928 patients were admitted during the study period and 351 were admitted to ICU on full code treatment and were discharged on exclusive palliative care with consensus of no readmission to ICU. The ICU readmission rate was 9.6%. Fourteen percent of the patients resumed their cancer treatment. The hospital mortality was 79.5%, the six-month mortality was 96.3% and the one-year mortality was 99.1%. From the 20.5% of patients who were discharged alive, most were on home nursing care (12%). Acute renal failure (OR = 2.42; CI 95%, 1.22-4.79) and delirium (OR = 1.92; CI 95%, 1,02-3.58) were identified as independent risk factors for hospital mortality.
**Conclusions:** Despite the high mortality rates, a significant proportion of patients (20%) was discharged from hospital, mainly with home nursing care. The ICU readmission and resume of cancer treatment rate for exclusive palliative patients reflect the need to improve the decision making process of end of life care. The knowledge about risk factors for mortality also provides consistent data for the decision-making process.

**References**

1. Taccone FS, Artigas AA, Sprung CL, Moreno R, Sakr Y, Vincent JL. Characteristics and outcomes of cancer patients in European ICUs. Crit Care 2009;13(1):R15.
2. Kostakou E, Rovina N, Kyriakopoulou M, Koulouris NG, Koutsoukou A. Critically ill cancer patient in intensive care unit: issues that arise. J Crit Care. 2014;29(5):817-22.
3. Guidelines for intensive care unit admission, discharge, and triage. Task Force of the American College of Critical Care Medicine, Society of Critical Care Medicine. Crit Care Med. 1999;27(3):633-8.

### P06

### Characteristics and outcomes of critical palliative patients in a private intensive care unit

Paulo Sergio Santos Oliveira, Eduardo da Rosa Borges, Djalma Barbosa Junior, Flavia Barboza
HFCP - Hospital Fornecedores de Cana de Piracicaba, Piracicaba, SP, Brasil
*Critical Care* 2017, **21(Suppl 2):**P06

**Introduction:** In intensive care unit (ICU), the palliative care (PC) plays an important role in critical illness. Ideally, PC delivery should be longitudinal, beginning at the time of a potentially life-limiting diagnosis and continuing throughout the course of the disease [1]. However, many patients only receive the initial palliative approach when they have already been hospitalized in the ICU.
**Objective:** The goal of the study is to describe the characteristics of patients in palliative care (PC) in ICU compared to the group not undergoing palliative care (nonPC). In addition, we made a subgroup analysis in the PC group, comparing oncologic (OC) and non oncologic (Non-OC) patients regarding demographic characteristics and outcomes.
**Methods:** A cross-sectional study was conducted based on a continuous register database. The ICU admissions, from September 2015 until November 2016, were included. Demographic information collected included age, gender, type admission (medical or surgical), comorbidities (actual neoplasms, chronic obstructive pulmonary disease, hypertension, diabetes, stroke, chronic kidney failure and performance status before hospital admission). The markers SAPS3 and SOFA score day 1 were collected. In addition, the use of life-enhancing measures (mechanical ventilation, vasoactive drugs and renal replacement therapy), the days of hospitalization prior to ICU, length of stay in the ICU and hospital were considered. Finally we evaluated the outcomes in the ICU and the hospital. For the quantitative variables was used the Mann–Whitney test. For the categorical variables, the chi-square test was used for comparison between groups. After univariate analysis all those with $p < 0.1$ were included in the multivariate analysis. Variables with $p < 0.05$ of the multivariate analysis were considered significant. We used R (v. 3.3.0) for all analysis.
**Results:** A total of 578 admission in ICU were evaluated, with the PC group including 46 (7,96%) e nonPC group 532 (92,04%) patients. After the multivariate analysis, factors such as age (odd: 1,04, CI: 1-1,08), oncological patients (5,67; 1,92-17,76), bedridden previous status (5,81; 1,74-20,37), clinical admission (8,72; 1,97-52,88), days of hospitalization in ICU (1,07; 1,01-1,12) and hospital mortality (8,39; 1,62-39,09) had significant association in PC. In the PC, only age (0,93; 0,88-0,99) was associated with the OC.
**Conclusions:** Patients in PC were older, with a significant functional limitation. The hospitalization was generally for clinical reasons, remaining more days hospitalized and with a higher hospital mortality in relation to the nonPC.

**Reference**

1. Levetown, Marcia MD, FAAHPM. Increasing Access to the Benefits of Palliative Care in the PICU. Pediatric Critical Care Medicine: August 2016 - Volume 17 - Issue 8 - p 804–805.

### P07

### Risk factors for intensive care acquired weakness: a systematic review and meta-analysis

Raquel Annoni[1], Jennifer Jones[2], Diogenes Seraphim Ferreira[3], Susan Berney[2], Linda Denehy[1]
[1]Unimelb - The University of Melbourne, Melbourne, Victoria, Australia; [2]Austin - Austin Health, Melbourne, Victoria, Australia; [3]Monash - Monash University, Melbourne, Victoria, Australia
*Critical Care* 2017, **21(Suppl 2):**P07

**Introduction:** Previous studies have shown discordant results on the risk factors and outcomes for intensive care acquired weakness (ICUAW).
**Objective:** We aimed to identify and synthetize the evidence about the prevalence, risk factors and outcomes of ICUAW in critically ill patients.
**Methods:** The systematic review was previously registered on International Prospective Register of Systematic Reviews (PROSPERO: CRD42014014521). Six electronic databases (PUBMED, MEDLINE, CINAHL, EMBASE, PEDro, SciELO) were searched from 2007 to 2017. Experimental and observational studies were eligible for inclusion if: 1) enrolled adult critically ill patients, 2) ICUAW was evaluated using clinical (MRC: Medical Research Council score), electrophysiological tests or a combination of both and, 3) reported at least one comparison between participants with and without ICUAW. A data collection form was developed and used to extract data from the included studies by one reviewer and cross-checked by a second reviewer. Studies using MRC score at awakening were included in a meta-analysis.
**Results:** Thirty-seven articles on 29 patient groups (n = 4011 patients) were included. Eighteen studies were conducted in Europe, 6 in the USA and 1 in each of the following countries: Australia, Brazil, Egypt, India and Vietnam. Twenty-two studies were cohorts, 6 randomized controlled trials (RCTs) and 1 cross-sectional study. Nine observational studies (9/23) and 2 RCTs (2/6) had low risk of bias. Fifteen studies used the MRC score to diagnose ICUAW and 14 used electrophysiological tests. Twenty-seven studies excluded patients with a previous history of neuromuscular disease. The pooled ICUAW prevalence (95% CI) was 40.1% (32.5, 47.7%) regardless of the diagnostic test used and time of first assessment. Eight studies evaluating 1488 patients were included in the meta-analysis. Risk factors for ICUAW measured with MRC score at awakening included older age, female gender, high SOFA score, sepsis on ICU admission and any use of corticosteroids during ICU stay (Table 1). In addition, ICUAW was associated with poor outcomes including longer ICU and hospital length of stay (Table 1).
**Conclusions:** Intensive care acquired weakness occurs in approximately 40% of general critically ill patients. The current meta-analysis provides evidence of risk factors for MRC diagnosed ICUAW. Further research should consider including these risk factors when building multivariable models to investigate the contributors to the development of ICUAW.

**Table 1 (Abstract P07).** ICUAW risk factors and outcomes

| | N | Effect estimate (95% CI) | $I^2$ (P value) |
|---|---|---|---|
| Age (years) | 5 | MD: 3.46 (0.94, 5.98) | 18% (p = 0.30) |
| Female gender | 4 | OR: 1.62 (1.22, 2.14) | 0% (P = 0.46) |
| SOFA score | 4 | MD: 1.96 (1.41, 2.50) | 0% (P = 0.77) |
| Sepsis on admission | 3 | OR: 1.48 (1.09, 2.00) | 0% (P = 0.62) |
| Use of corticosteroids | 3 | OR: 2.17 (1.21, 3.91) | 45% (P = 0.16) |
| ICU LOS (days) | 3 | MD: 8.67 (7.05, 10.28) | 0% (P = 0.85) |
| Hospital LOS (days) | 2 | MD: 15.31 (11.02, 19.61) | 0% (P = 0.67) |

*MD* mean difference, *OR* odds ratio, *LOS* length of stay

### P08

### Antibiotics use in intensive care units of a public hospital in the State of Ceará, Brazil

Antonio Pergentino Barreira Neto, Alana de Alcântara Brito, Milena de Azevedo Teles, Rafael Cabral Teixeira, Stephanie Wilkes da Silva, Iara Serra Azul Machado Bezerra, Marina Parente Albuquerque, Carlos Augusto Ramos Feijó, Francisco Albano de Meneses
HGF – Hospital Geral de Fortaleza, Fortaleza, CE, Brasil
*Critical Care* 2017, **21(Suppl 2):**P08

**Introduction:** The use of antibiotics (ATB) are becoming quite common in intensive care units (ICUs) throughout the world, being present in prescriptions of the vast majority of patients.
**Objective:** To seek for substrates capable to contribute in future optimization of use of antibiotics, due to the emergence of resistant microorganisms.
**Methods:** Retrospective study performed through database analysis of patients admitted to three ICUs of the Hospital Geral de Fortaleza, from October 2016 to January 2017.
**Results:** Our sample included 134 patients, mostly male (54.5%), age average of 53.5 ± 19 years (median: 55.5 years), average SOFA at admission was 5.6 ± 4.6 points (median: 5 points), length of stay average of 17.8 ± 17.5 days (median: 12 days), ATB use average, per patient, was 4.4 ± 3.8 (median: 4) and mortality reached 26.1%. In this group of patients, 119 (88.8%) used ATB at any moment during hospitalization time and 15 (11.2%) did not use it at all. Comparing the two groups, we noticed a higher SOFA score average among those ar used ATB (6.2 x 1.1, $p < 0.05$), and higher mortality rates (29.4% x 0%, p < 0,05). The most commonly used ATBs were meropenem (55.4%), piperacillin-tazobactam (50.4%) and polymyxin B (44.5%). SOFA score average and mortality were particularly higher considering patients in whom polymyxin B was used, than in non-users. Still regarding polymyxin B usage, the drug was prescribed to 6 (4.5%) patients immediately upon ICU admission.
**Conclusions:** The use of ATB remains very prevalent among patients admitted to ICUs. In addition, we were surprised by the amount of polymyxin B usage, reaching almost half of the studied population, and even prescribed to some patients right on ICU arrival. This leads us to imagine that an expressive part of our ICU patients are infected by resistant microorganisms, increasing the chance of treatment failure.

### P09

### Women's participation in authorship of original articles in intensive care

Carlos Augusto Ramos Feijó, Marina Parente Albuquerque, Francisco Albano de Meneses
HGF - Hospital Geral de Fortaleza, Fortaleza, CE, Brasil
*Critical Care* 2017, **21(Suppl 2):**P09

**Introduction:** Throughout the world, research shows that the participation of women as authors of original articles in the medical field is still not very representative.
**Objective:** To identify the participation of women as authors of original articles in the field of Intensive Care in Brazil.
**Methods:** Observational study of women, either as an author in general or as the first author, of original articles published in the Revista Brasileira de Terapia Intensiva (RBTI), from 2006 to 2015. The RBTI is an indexed quarterly publication of the Associação de Medicina Intensiva Brasileira and the Sociedade Portuguesa de Cuidados Intensivos. Review articles, case reports, comments, letters to editors and guidelines were excluded from the sample. It was not possible to discriminate the professional categories of the authors.
**Results:** We identified 356 original articles, with 2,049 authors, of which 48.5% were women. The mean number of authors was 5.8 (men: 3, women: 2.8). The regions with the highest prevalence of female authors were Southeast [SE] (52.9%), South [S] (26.1%) and Northeast (17.2%). More than half of the authors came from two states: São Paulo [SP] (37.3%) and Rio Grande do Sul [RS] (13.9%). Five of the 27 units of the federation (UF) had no authors in the period. From the total number of articles, 51 (14.3%) were written exclusively by women and 40 (11.2%) only by men. The regions that contributed with the largest portion of the articles published only by women were SE (63%) and S (20.4%), with 52% of them from SP and 13% from RS. Of the 356 main authors, 198 (55.6%) were women, predominantly from SE (55.1%). Only 14 of the 27 UF had women as the main authors, with SP (41.9%) and RS (14.1%) being the most representative. We also observed the participation of 40 women from other countries in the authorship of the articles, being 8 main authors. During the analyzed period, there was a predominance in authorship of women between 2007 and 2009 and between 2011 and 2014.
**Conclusions:** In Intensive Care publishing environment, the participation of Brazilian women in generating medical research, unlike that perceived in other countries, accompanies pari passu the male production. It should be stressed that they take the lead when one considers the position of first author.

#### References

1. Filardo G, da Graca B, Sass DM, Pollock BD, Smith EB, Ashley-Marie Martinez M. Trends and comparison of female first authorship in high impact medical journals: observational study (1994-2014). BMJ. 2016; 352:i847.
2. Erren TC, Groß JV, Shaw DM, Selle B. Representation of women as authors, reviewers, editors in chief, and editorial board members at 6 general medical journals in 2010 and 2011. JAMA Intern Med. 2014; 174(4):633-635.
3. Jagsi R, Guancial EA, Worobey CC, Henault LE, Chang Y, Starr R, et al. The "gender gap" in authorship of academic medical literature – a 35-year perspective. N Engl J Med. 2006; 355:281-87.

### P10

### Medical and surgical admission in an oncology ICU in the northeast of Brazil

Ana Paula Pierre Moraes, Gustavo Teixeira Alves, José Ricardo Santos Lima, Karina Viegas
HTLF - Hospital de Câncer do Maranhão Tarquinio Lopes Filho, São Luis, MA, Brasil
*Critical Care* 2017, **21(Suppl 2):**P10

**Introduction:** Advances in oncology have lead to reduction in mortality rates nowadays. The cancer patients are usually admitted to the ICU due to postoperative high-risk surgeries, clinical complications due to acute process, especially infection/sepsis and complications due to chemotherapy [1,2].
**Objective:** To evaluate the hospital mortality and morbidities outcomes in medical and surgical cancer patients requiring ICU admission
**Methods:** Retrospective study conducted in 11-bed ICU of a public cancer hospital in São Luis-Maranhão, northeast of Brazil. All patients with a definitive cancer diagnosis requiring ICU from January to December 2016 were classified based on the reason of ICU´s medical and surgical admissions. We evaluate demographic and clinical variables at ICU admission, ICU support and outcomes: ICU and hospital length of stay (LOS), ICU re-admission, nosocomial ICU infection, and ICU and hospital mortality, respectively. The statistical difference was tested using Pearson´s chi-square or Mann-Whitney tests. The significance level adopted was 0,05.
**Results:** 495 patients fulfilled the study criteria, 239 (48%) were admitted due to medical reasons and 256 (52%) due to surgical reasons. The main reasons for intensive care admission were postoperative care after elective surgery (47%), infection/sepsis (15%) and respiratory failure (10%). Medical and surgical admission did not differ in sex, age, nosocomial ICU infection and hospital LOS. Medical admission of cancer patients had higher SAPS 3 and SOFA scores, higher need for mechanical ventilation and vasopressors, higher length in ICU (p < 0,001 for all). The overall ICU and hospital mortality were 32% and 50% respectively; 55% and 80% for medical ICU admission and 10% and 25% for surgical ICU admission (p < 0,001) (Table 2).

**Conclusions:** Cancer patients that required ICU admission due to medical reasons were sicker at ICU admission and had worse outcomes compared to those admitted due to surgical reasons. The results corroborate the importance of early diagnosis, access to medical attendance and prompt reference. The possibility to early ICU admission may offer opportunities to prevent and better manage life-threatening complication.

**References**

1. Kostakou E, et al. J Crtit Care 2014 Oct; 29(5):817-22.
2. Soares M ,et al. Crit Care Med 2010 Jan; 38(1):9-15.

**Table 2 (Abstract P10).** Characteristics

| Characteristics | All n = 495 | Surgical n = 256 (51,7%) | Medical n = 239 (48,3%) | p |
|---|---|---|---|---|
| *Demographic variables* | | | | |
| Age | 60,0 (48-72) | 60,5 (51-72) | 60,0 (46-71) | 0,30 |
| Gender Male | 251 (51,7%) | 123 (48,0%) | 128 (53,6%) | 0,22 |
| *Anatomic Tumor Site* | | | | |
| Gastric and Esofageal | 92 (18,6%) | 63 (24,6%) | 29 (12,1%) | <0,001 |
| Colorectal | 35 (7,1%) | 28 (10,9%) | 7 (2,9%) | <0,01 |
| Uterine cervix | 56 (11,3%) | 24 (9,4%) | 32 (13,4%) | 0,16 |
| Ovarian | 42 (8,5%) | 31 (12,1%) | 11 (4,6%) | <0,01 |
| Prostate | 42 (8,5%) | 19 (7,4%) | 23 (9,6%) | 0,38 |
| Lung | 34 (6,9%) | 13 (5,1%) | 21 (8,8%) | 0,10 |
| Hematology | 59 (11,9%) | 3 (1,2%) | 56 (23,4%) | <0,001 |
| *Clinical variables* | | | | |
| Length hospital stay prior ICU | 2,0 (1-11) | 2,2 (1-10) | 3,0 (0-12) | 0,03 |
| Charlson comorbity Index (points) | 2,0 (2-4) | 2,0 (2-3) | 2,0 (2-6) | <0,001 |
| SAPS 3 (points) | 48,0 (35-65) | 35,5 (29-42) | 64,0 (56-77) | <0,001 |
| SOFA score on D1 (points) | 4,0 (2-7) | 2,0 (1-5) | 6,0 (3-8) | <0,001 |
| *ICU support* | | | | |
| Mechanical ventilantion on 1 h | 175 (35,4%) | 81 (31,6%) | 94 (39,3%) | <0,001 |
| Vasoactive drusg on 1 h | 61 (12,3%) | 11 (4,3%) | 50 (20,9%) | <0,001 |
| Mechanical ventilation | 209 (42,2%) | 83 (32,4%) | 126 (52,7%) | <0,001 |
| Vasoactive drugs | 79 (16,0%) | 17 (6,6%) | 62 (25,9%) | <0,001 |
| Renal replacement therapy | 25 (5,1%) | 5 (2,0%) | 20 (9,2%) | 0,001 |
| *Outcomes* | | | | |
| Readmission | 46 (9,3%) | 22 (8,6%) | 24 (10,0%) | 0,57 |
| Nosocomial ICU infection | 19 (3,8%) | 7 (2,7%) | 12 (5,0%) | 0,19 |
| ICU Los | 3 (1-6) | 2,0 (1-4) | 5,0 (2-9) | 0,001 |
| Hospital Los | 16 (8-28) | 16,0 (8-29) | 17,0 (7-28) | 0,84 |
| ICU mortality | 157 (31,7%) | 25 (9,8%) | 132 (55,2%) | <0,001 |
| Hospital mortality | 248 (50,1%) | 62 (24,7%) | 187 (80,3%) | <0,001 |

Results for continuous variables are reported as median (interquartile range)

### P11

### Acute Kidney Injury (AKI) related to pregnancy, mortality and survival of patients treated at the Maternal Intensive Care Unit of Hospital Materno Infantil de Brasília (ICU/HMIB)

Dilson Palhares Ferreira[2], Aline Mizusaki Imoto[2], Fábio Ferreira Amorim[1], Amanda Jacomeli Matsuura[1], Jaqueline Lima de Souza[3], Adriell Ramalho Santana[1]
[1]FEPECS - Fundação de Ensino e Pesquisa em Ciências da Saúde, Brasília/Distrito Federal, Brasil; [2]FEPECS - Programa de Pós Graduação em Ciências da Saúde, Brasília/Distrito Federal, Brasil; [3]ESCS - Escola Superior de Ciências da Saúde, Brasília/Distrito Federal, Brasil
*Critical Care* 2017, **21(Suppl 2):**P11

**Introduction:** Pregnancy-related acute kidney injury (AKI) can be defined as the abrupt decline of renal function during pregnancy or postpartum period[1]. It's important cause of maternal and fetal morbidity and mortality.

**Objective:** We attempted to classify the renal function of patients admitted to a maternal intensive care unit by KDIGO Criteria [2] and to evaluate the impact of each category on mortality in a maternal ICU.

**Methods:** This was a retrospective analysis of observational data prospectively collected from January 2014 to April 2016 in the maternal ICU/HMIB, Brasilia, Federal District, Brazil. All consecutive patients diagnosed with AKI were included. Patients with a previous diagnosis of renal failure prior to pregnancy or kidney transplantation were excluded.

**Results:** From a total of 619 patients admitted in the ICU during the study period, pregnancy related AKI was present in 172 cases (27,8%). From the 172 patients with pregnancy related AKI, average age was 29 ± 7 years, gestational age was 32.5 ± 5.7 weeks, APACHE II was 12 ± 7, and SOFA score was 3 ± 3. The major causes of ICU admission were eclampsia (N = 63, 36.6%), preeclampsia (N = 39, 22.7%) and sepsis (N = 27, 15.7%). Fifty-three patients had prior arterial systemic hypertension (30.8%) and 15 had heart failure (8.7%). Thirteen parturients received no prenatal care (7.6%) and 133 had cesarian delivery (71.5%). ICU length of stay was 7.6 ± 10.2 days and ICU maternal mortality was 8.7% (N = 15), and newborn mortality was 14.5% (N = 25). One hundred ten patients were classified as KDIGO 1 (64.0%, ICU mortality rate of 0.9%), 43 as KDIGO 2 (20.9%, ICU mortality rate of 11.1%), and 22 patients as KDIGO 3 (15.1%, ICU mortality rate of 38.5%). Significant difference was observed in the Kaplan–Meier survival curves among KDIGO stages at 28 days, P = 0.00 (see Fig. 1). Indeed, there was significant difference in the Kaplan–Meier survival curves of the patients classified as KDIGO 3 with or without need for hemodialysis at 28 days, P = 0.29 (see Fig. 2). None of patients with conservative renal treatment classified as KDIGO stage 3 evolved to death. These aspects reinforces the importance of indication of renal replacement therapy at the appropriate time, as well as to classify AKI, especially in critical ill patients, since this makes it possible for predict prognosis.

**Conclusions:** KDIGO Criteria[2] was directly related to mortality in the pregnancy-related AKI, notably those who needed renal replacement therapy.

**References**

1. Hildebrand AM et al. Characteristics and Outcomes of AKI Treated with Dialysis during Pregnancy and the Postpartum Period. J Am Soc Nephrol. 2015 Dec;26(12):3085-91.
2. Kidney International Supplements. 2012 March; 2:124–138.

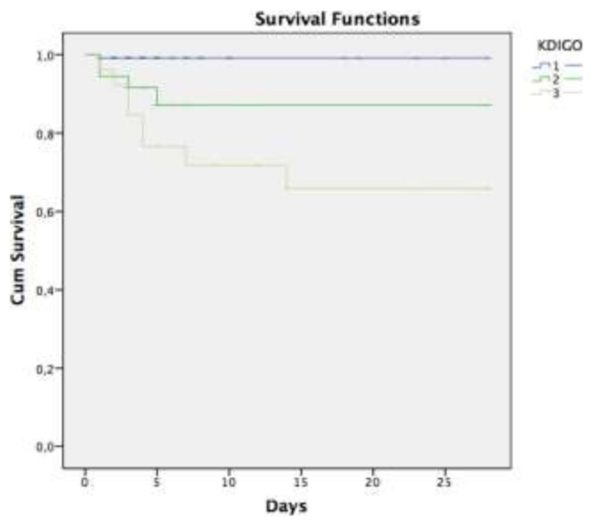

**Fig. 1 (Abstract P11).** Kaplan Meier curve to perform survival analysis in 28 days, with the KDIGO classification stages of patients with pregnancy-related AKI admitted to the ICU/HMIB, Jan/2014 to Apr/2016

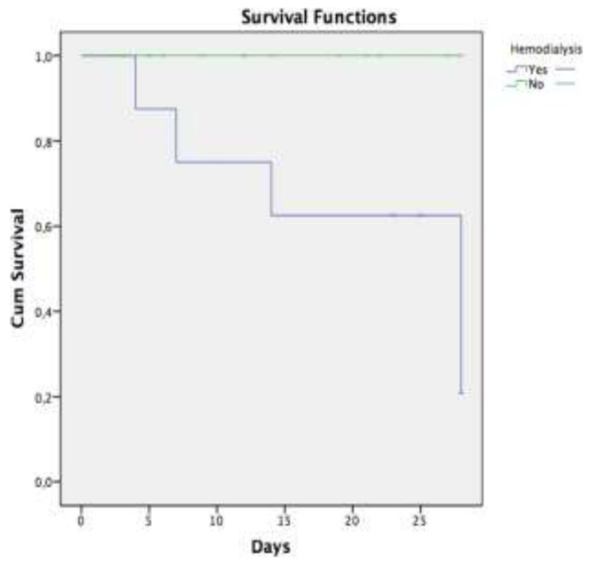

**Fig. 2 (Abstract P11).** Kaplan Meier Curve for the evaluation of 28-day survival of pregnant patients admitted to ICU/HMIB, stage 3 AKI (KDIGO), with and without need for hemodialysis

### Hemodynamics/Shock

### P12

### Plant poisoning: cardiologic and neurologic manifestations

Yuri Cavalcanti Albuquerque Tenorio, Amauri Clemente da Rocha, Helena Moraes da Silva
UNCISAL - Universidade Estadual de Ciências da Saúde de Alagoas, Maceió, AL, Brasil
*Critical Care* 2017, **21(Suppl 2):**P12

**Introduction:** Plant intoxication is a common topic in the veterinary medicine; however, when compared to the human medicine, this topic is not as known beyond the health professionals. Plants produce secondary metabolites, some are used to defend themselves from predators; consequently, some species are toxic to humans and other animals. The symptoms of poisonous plants intoxication vary from a simple pruritus to a neurologic disturbance leading to coma and death.

**Objective:** Facilitate the identification of cardiologic and neurologic symptoms of patients with history of plant poisoning.

**Methods:** This paper is a literature review discussing published information on cardiologic and neurologic manifestations of plant poisoning of the most common poisonous plants in Brazil.

**Results:** The cardioneurologic manifestations have a variety of different symptoms; consequently, the manifestations were divided into three groups (Fig. 3). The first group is characterized by an atropine-like intoxication, with a rapid onset of nausea and vomiting. Then the skin becomes dry, with facial flushing, dry mucous membranes, tachycardia, mydriasis, psychomotor agitation, fever, behavioral disorders, hallucinations and delusions. In severe cases occurs neurological depression and coma, cardiovascular, respiratory and death disorders. The second group is the digitalis-like intoxication. The ingestion causes pain followed by sialorrhea, nausea, vomiting, abdominal cramps, and diarrhea. The neurological manifestations are headache, dizziness, mental confusion and visual disturbances and the cardiovascular disorders are arrhythmias, bradycardia, and hypotension. The last group consists in cellular anoxia caused by cyanuric acid. There are gastrointestinal disorders causing nausea, vomiting, abdominal cramps, diarrhea, that might lead to metabolic acidosis. The neurological manifestations are drowsiness, numbness, seizures and coma. A typical crisis consists in a triad of opisthotonus, trismas and mydriasis. This subgroup also has respiratory disorders, such as dyspnea, apnea, secretions, cyanosis, and, finally, the cardiocirculatory disorders are hypotension in the final phase.

**Conclusions:** Some species of poisonous plants have metabolites that cause life-threatening symptoms; consequently, the physician must be aware of the different clinical features from the big variety of poisonous plants.

### References

1. Beyer J, Drummer O & Maurer H. Analysis of toxic alkaloids in body samples. Foren Scien Internat, 2009. 185, 1-9. doi:doi:10.1016/j.forsciint.2008.12.006
2. SINITOX: Dados de intoxicação, Brasil, 2013. Retrieved from: http://sinitox.icict.fiocruz.br/dados-nacionais in 2/25/2017, at 10:04 AM
3. Diaz J. Poisoning by Herbs and Plants: Rapid Toxidromic Classification and Diagnosis. Wilde & Environ Med, 2016. 27, 136-152.
4. Nikolau P, et al. Accidental poisoning after ingestion of "aphrodisiac" berries: diagnosis by analytical toxicology. The Journal of Emergency Medicina 2012. 42(6), 662-5.

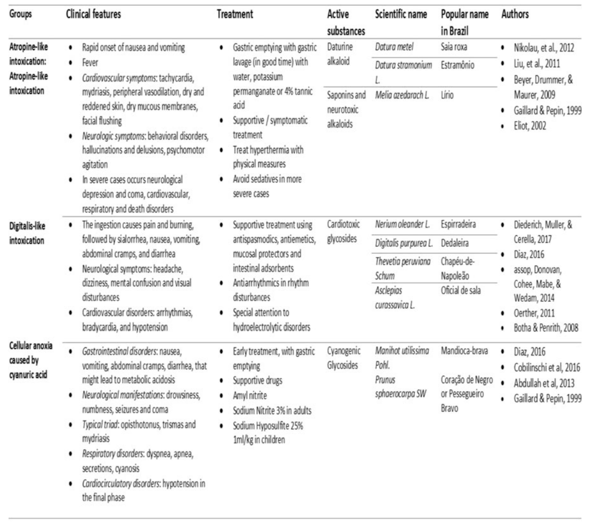

| Groups | Clinical features | Treatment | Active substances | Scientific name | Popular name in Brazil | Authors |
|---|---|---|---|---|---|---|
| **Atropine-like intoxication: Atropine-like intoxication** | • Rapid onset of nausea and vomiting<br>• Fever<br>• *Cardiovascular symptoms*: tachycardia, mydriasis, peripheral vasodilation, dry and reddened skin, dry mucous membranes, facial flushing<br>• *Neurologic symptoms*: behavioral disorders, hallucinations and delusions, psychomotor agitation<br>• In severe cases occurs neurological depression and coma, cardiovascular, respiratory and death disorders | • Gastric emptying with gastric lavage (in good time) with water, potassium permanganate or 4% tannic acid<br>• Supportive / symptomatic treatment<br>• Treat hyperthermia with physical measures<br>• Avoid sedatives in more severe cases | Daturine alkaloid | *Datura metel* | Saia roxa | • Nikolau, et al., 2012<br>• Liu, et al., 2011<br>• Beyer, Drummer, & Maurer, 2009<br>• Gaillard & Pepin, 1999<br>• Eliot, 2002 |
| | | | | *Datura stramonium L.* | Estramônio | |
| | | | Saponins and neurotoxic alkaloids | *Melia azedarach L.* | Lírio | |
| **Digitalis-like intoxication** | • The ingestion causes pain and burning, followed by sialorrhea, nausea, vomiting, abdominal cramps, and diarrhea<br>• Neurological symptoms: headache, dizziness, mental confusion and visual disturbances<br>• Cardiovascular disorders: arrhythmias, bradycardia, and hypotension | • Supportive treatment using antispasmodics, antiemetics, mucosal protectors and intestinal adsorbents<br>• Antiarrhythmics in rhythm disturbances<br>• Special attention to hydroelectrolytic disorders | Cardiotoxic glycosides | *Nerium oleander L.* | Espirradeira | • Diederich, Muller, & Cerella, 2017<br>• Diaz, 2016<br>• assop, Donovan, Cohee, Mabe, & Wedam, 2014<br>• Oerther, 2011<br>• Botha & Penrith, 2008 |
| | | | | *Digitalis purpurea L.* | Dedaleira | |
| | | | | *Thevetia peruviana Schum* | Chapéu-de-Napoleão | |
| | | | | *Asclepias curassavica L.* | Oficial de sala | |
| **Cellular anoxia caused by cyanuric acid** | • *Gastrointestinal disorders*: nausea, vomiting, abdominal cramps, diarrhea, that might lead to metabolic acidosis<br>• *Neurological manifestations*: drowsiness, numbness, seizures and coma<br>• *Typical triad*: opisthotonus, trismas and mydriasis<br>• *Respiratory disorders*: dyspnea, apnea, secretions, cyanosis<br>• *Cardiocirculatory disorders*: hypotension in the final phase | • Early treatment, with gastric emptying<br>• Supportive drugs<br>• Amyl nitrite<br>• Sodium Nitrite 3% in adults<br>• Sodium Hyposulfite 25% 1ml/kg in children | Cyanogenic Glycosides | *Manihot utilissima Pohl.*<br>*Prunus sphaerocarpa SW* | Mandioca-brava<br>Coração de Negro or Pessegueiro Bravo | • Diaz, 2016<br>• Cobilinschi et al, 2016<br>• Abdullah et al, 2013<br>• Gaillard & Pepin, 1999 |

**Fig. 3 (Abstract P12).** Clinical features of poisonous plants intoxication with cardiologic and neurologic manifestations divided into three different groups with each etiology and treatment. Results from the literature review

### P13

### Microvascular reactivity in patients with and without circulatory shock: an exploratory analysis

Renato Carneiro de Freitas Chaves, Roberto Rabello Filho, Ary Serpa Neto, Murillo Santucci Cesar de Assunção, Adriano José Pereira, Flavia Fernandes Manfredi de Freitas, Maria Laura Romagnoli, Thiago Domingos Corrêa
HIAE - Hospital Israelita Albert Einstein, São Paulo, SP, Brazil
*Critical Care* 2017, **21(Suppl 2):**P13

**Introduction:** Abnormalities in microvascular reactivity accessed with thenar near-infrared spectroscopy (NIRS) with a vascular occlusion test (VOT) have been described in critically ill patients [1].
**Objective:** Our objective was to confirm such findings in terms of static and dynamic NIRS derived parameters in health volunteers and in critically ill patients with and without circulatory shock.
**Methods:** This prospective single-center study was approved by the ethics committee of Hospital Israelita Albert Einstein. Written informed consent was obtained from each participant. Twenty adult healthy volunteers [29 (27-34) years, median (IQR)] and 40 critically ill patients with and without shock (n = 20, each) admitted to the ICU within 24 h were included in this study. Tissue O2 saturation (StO2) was measured at the thenar eminence using an InSpectra StO2 Tissue Oxygenation Monitor (model 650; Hutchinson Technology, Hutchinson, MN, USA) using a 15 mm probe. Vascular occlusion test was performed by inflating a sphygmomanometer in the upper arm 30 mmHg above the systolic arterial pressure, which was quickly deflated after 3 min of ischemia (1). A research software (Hutchinson Technology Inc., Hutchinson, MN) was used for data collection and analysis.
**Results:** Shock patients (80% septic shock; 20% cardiogenic shock) were older than non-shock patients [66 (56-73) vs. 50 (44-60) years, median (IQR); p = 0.024] and more frequently female [11 (55%) vs. 6 (30%); p = 0.017). Shock patients had a higher SAPS III score [53 (45-65) vs. 30 (22-46); p < 0.001], higher SOFA score [8 (6-10) vs. 4 (1-5); p < 0.001] and higher 28-day mortality [5 (25.0%) vs. 0 (0.0%); p = 0.047). Shock patients showed a lower maximum StO2 after VOT than patients without shock (Fig. 4). Recovery time and hyperemia area differed between health volunteers and critically ill patients, but did not differ between patients with and without shock.
**Conclusions:** In our studied population, NIRS static and dynamic parameters poorly discriminate shock and non-shock patients. The role of thenar near-infrared spectroscopy in the care of critically ill patients needs to be further addressed in large clinical trials.

#### Reference

1. Lima A et al. The relation of near-infrared spectroscopy with changes in peripheral circulation in critically ill patients. Crit Care Med. 2011; 39(7):1649-54

| Characteristics | Volunteers | Non-shock | Shock | P value |
|---|---|---|---|---|
| $StO_2$, % | 78 (76-81) | 86 (76-90) | 81 (76-83) | 0.096 |
| $StO_2$ min, % | 50 (47-55) | 55 (53-65)# | 50 (47-57) | 0.030 |
| $StO_2$ max, % | 94 (89-95) | 93 (90-95) | 87 (80-92)&¥ | 0.007 |
| Descending slope, %/min | 9.1 (7.6-10.2) | 8.4 (6.0-9.8) | 7.9 (6.7-9.4) | 0.527 |
| Ascending slope, %/sec | 3.8 (3.2-4.6) | 2.2 (1.6-3.4)# | 2.1 (1.2-3.1)& | 0.001 |
| Recovery time, sec | 12.5 (12.0-14.0) | 16.5 (13.0-24.0)# | 24.0 (16.0-32.0)& | <0.001 |
| AUC | 21.2 (14.6-26.6) | 8.9 (4.0-13.3)# | 8.6 (4.7-15.2)& | <0.001 |

Values represent median (IQR). AUC: area under the curve of reactive hyperemia. Comparisons significant at the 0.016 level: #: Non-shock vs. Volunteers, &: Shock vs. Volunteers and ¥: Shock vs. Non-shock.

**Fig. 4 (Abstract P13).** NIRS derived variables

### P14

### Effect of extracorporeal membrane oxygenation on microcirculation and tissue oxygen saturation in ARDS: a case report

Renato Carneiro de Freitas Chaves, Murillo Santucci Cesar de Assunção, Ary Serpa Neto, Roberto Rabello Filho, Bruno de Arruda Bravim, Philipe Franco do Amaral Tafner, Carmen Silva Valente Barbas, Thiago Domingos Corrêa
HIAE - Hospital Israelita Albert Einstein, São Paulo, SP, Brazil
*Critical Care* 2017, **21(Suppl 2):**P14

**Introduction:** The impact of ECMO on microcirculation and tissue oxygenation (StO2) in patients with acute respiratory distress syndrome (ARDS) is poorly understood.
Objective: Our objective was to evaluate the effect of veno-venous ECMO (VV-ECMO) on microcirculation and tissue oxygen saturation during the first 24 h on ECMO support.
**Methods:** A written informed consent was obtained from a next of kin of the patient. We report a case of a 27-year-old Brazilian woman with diagnosis of Granulomatosis with Poliangiitis. The patient was referred to our intensive care unit (ICU) due to a severe ARDS requiring venous-venous ECMO support. Sublingual microcirculation [Cytocam-IDF imaging® (Braedius Medical BV, Huizen, Netherlands)] and thenar StO2 [InSpectra StO2 Tissue Oxygenation Monitor (model 650; Hutchinson Technology, Hutchinson, MN, USA)] were measured immediately before ECMO (Baseline), 4 h (T4h) and 24 h (T24h) after the beginning of ECMO [1,2]. Reported parameters from Cytocam-IDF were total vessel density (TVD; mm/mm2), proportion of perfused vessels (PPV; %), perfused vessel density (PVD; mm/mm2) and microvascular flow index (MFI). Vascular occlusion test (VOT) was performed by inflating a sphygmomanometer in the upper arm 30 mmHg above the systolic arterial pressure, which was quickly deflated after 3 min of ischemia[2]. A research software (Hutchinson Technology Inc., Hutchinson, MN) was used for NIRS data collection.
**Results:** After 24 h of VV-ECMO, clinical parameters improved, but the microcirculatory parameters did not (Fig. 5). After 24 h of ECMO, PPV increased, TVD and PVD decreased and MFI remained constant (Fig. 5). While Basal StO2 remained stable after 4 h on ECMO, StO2 min and max after VOT improved and ascending slope worsened. The patient died after 19 days in the ICU.
**Conclusions:** Microcirculation abnormalities and microvascular reactivity in ARDS patients on ECMO and their relationship with outcomes in this population of critically ill patients remain poorly understood and need to be evaluated in future studies.

#### References

1. Aykut G, et al. Cytocam-IDF (incident dark field illumination) imaging for bedside monitoring of the microcirculation. Intensive Care Med Exp. 2015;3:40.
2. Lima A, et al. The relation of near-infrared spectroscopy with changes in peripheral circulation in critically ill patients. Crit Care Med. 2011; 39(7):1649-54.

| | Baseline | T4h | T24h |
|---|---|---|---|
| **Patients Characterist** | | | |
| MAP, mmHg | 83 | 69 | 66 |
| Cardiac index, l/min/m² | 6.9 | 5.4 | 6.6 |
| Arterial pH | 7.02 | 7.08 | 7.13 |
| $PaO_2$, mmHg | 140 | 138 | 124 |
| $PaCO_2$, mmHg | 68 | 41 | 56 |
| Arterial lactate, mg/dl | 47 | 39 | 37 |
| **CytoCam** | | | |
| TVD, mm/mm² | 22.2 (21.9-22.5) | 18.4 (17.3 – 19.5) | 17.4 (14-19.9) |
| PPV, % | 60.9 (51.5-64.6) | 47.5 (28.2-66) | 77.5 (57.4-90.9) |
| PVD, mm/mm² | 13.5 (11.6-14.2) | 9.3 (5.1-11.4) | 12.7 (9.9-15.4) |
| MFI | 3 (3-3) | 3 (3-3) | 3 (3-3) |
| **NIRS** | | | |
| $StO_2$, % | 53 | 53 | 45 |
| $StO_2$ min, % | 33 | 44 | 35 |
| $StO_2$ max, % | 63 | 65 | 55 |
| Descending slope, %/min | -7.00 | -4.86 | |
| Ascending slope, %/sec | 26 | 20 | |

Data presented as median (IQR).

**Fig. 5 (Abstract P14).** Microcirculatory parameters

### P15

### Accuracy of arterial pressure measurement in critically ill patients: The impact of the central to radial pressure gradient

Rogerio Passos, Adelmo Oliveira, Michel Ribeiro, Joao Ramos, Mauricio Teixeira, Andre Gobatto, Marcel Miranda, Paulo Batista
HSR - Hospital São Rafael, Salvador, Bahia, Brasil
*Critical Care* 2017, **21(Suppl 2):**P15

**Introduction:** Invasive arterial pressure monitoring is essential in managing critically ill patients. Therefore, the method of measurement and subsequent accuracy of blood pressure values is important. The radial site is most commonly used but, in patients requiring vasoactive drugs, gradients in mean arterial pressure (MAP) may develop from the central to the peripheral arterial tree.
**Objective:** The aim of this study is to evaluate the presence and determinants of femoral–radial gradients in MAP in a critically ill population.
**Methods:** This was a prospective observational study. Twenty-nine critically ill patients with clinical indication of invasive arterial pressure monitoring were included in the study. Simultaneous measurements were registered in central (femoral) and peripheral (radial) arteries in a medical-surgical intensive care unit. Bias and precision between simultaneous measurements of MAP via the femoral and radial arteries were determined by Bland–Altman analysis; hemodynamic and demographic factors associated with a MAP gradients were assessed by multiple linear regression
**Results:** 215 observations were made in 29 patients. Mean age of patients was 65 (SD +/- 14) years, and mean APACHE II score was 24 (S+/- 8). Overall mean bias between radial and femoral MAP measurements was 6,7 mmHg (limits of agreement, −2.2 to 9.5 mmHg). Multivariate analysis demonstrated that fluid responsive patients with systolic volume variation (SVV) higher than 15%, norepinephrine dose higher than 0,6 mcg/kg/min and higher BMI were associated with MAP gradient.
**Conclusions:** Our study demonstrated a systematic difference in MAP measured at the radial and femoral sites. The femoral artery may be the preferred site of measurement in some group of patients.

### P16

### Assessment of fluid responsiveness in spontaneously breathing patients: a systematic review of literature

Renato Carneiro de Freitas Chaves, Thiago Domingos Corrêa, Ary Serpa Neto, Bruno de Arruda Bravim, Ricardo Luiz Cordioli, Fabio Tanzillo Moreira, Murillo Santucci Cesar de Assunção
HIAE - Hospital Israelita Albert Einstein, São Paulo, SP, Brazil
*Critical Care* 2017, **21(Suppl 2):**P16

**Introduction:** Intravascular volume expansion is a common intervention in critically ill patients. Assessment of fluid responsiveness prior to volume expansion is critical to avoid fluid overload, which has been associated with poor outcomes. Maneuvers to assess fluid responsiveness are well established in mechanically ventilated patients [1]; however, few studies evaluated maneuvers to predict fluid responsiveness in spontaneously breathing patients.
**Objective:** Our objective was to perform a systematic review of literature addressing the available methods to assess fluid responsiveness in spontaneously breathing patients.
**Methods:** Studies were identified through electronic literature search of PUBMED from 01/08/2009 to 01/08/2016 by two independent authors. Original articles were selected for inclusion if one of the following definitions of fluid responsiveness was adopted: increase in stroke volume ≥10%, cardiac output ≥10%, cardiac index ≥10% or aortic velocity-time integral (VTI) ≥10% after a fluid challenge. No restrictions on language or clinical scenario were adopted. Intensive care unit (ICU), emergency department (ED) and operating room (OR) patients were included. Fluid challenge was deemed adequate if at least 5 ml/kg over 30 minutes were intravenously infused. Quality of included studies was evaluated with Quality Assessment of Diagnostic Accuracy Studies tool. Primary endpoint was to summarize methods of assess fluid responsiveness assessment in spontaneously breathing patients. Secondary end point was to construct a receiver operating characteristics curve (ROC) for the methods found in literature. Review Manager (RevMan) [Computer program], Version 5.3. Copenhagen, 2014 was used to create the ROC curves.
**Results:** Our search strategy identified 6,156 studies, and three studies were added through manual search. Of these, seven studies (5 ICU patients, 1 OR and 1 ED patients) were retrieved and included in this analysis. In total, 329 spontaneously breathing patients were assessed for fluid responsiveness. Of these, 171 (52%) were deemed fluid responsive. Eighteen maneuvers to assess fluid responsiveness in spontaneous breathing in patients were found (Fig. 6). Deep inspiration maneuver-induced change in pulse pressure and deep inspiration maneuver-induced change in velocity peak of femoral artery flow showed the highest accuracy to predict fluid responsiveness in this population of patients (Fig. 7).
**Conclusions:** Our systematic review indicates that spontaneous breathing is not a limitation to accurately assess fluid responsiveness in critically ill patients. Further well-designed studies, with adequate simple size and power, are necessary to confirm the real accuracy of the different methods used to assess fluid responsiveness in this population of patients.

**Reference**
1. Marik PE, et. al. Dynamic changes in arterial waveform derived variables and fluid responsiveness in mechanically ventilated patients: a systematic review of the literature. Critical care medicine. 2009;37(9):2642-7

| Author, year | Maneuver | Sensibility | Specificity | PPV | NPV | AUC [± SD or (95%CI)] |
|---|---|---|---|---|---|---|
| Airapetian et al, 2015 | cIVC>42% | 0.31 | 0.97 | 0.90 | 0.60 | 0.62 (0.66-0.88) |
| | IVCmax at baseline<2.1 cm | 0.93 | 0.33 | 0.57 | 0.83 | 0.62 (0.49-0.75) |
| | ΔCO-PLR > 10 % | 0.52 | 0.87 | 0.79 | 0.65 | 0.78 (0.66-0.88) |
| Duus et al, 2015 | PLR | 0.80 | 0.61 | 0.79 | 0.65 | 0.74 (0.65-0.83) |
| Hong et al, 2014 | $PPV_{FIB}$ of 13.7% | 0.90 | 0.87 | 0.87 | 0.90 | 0.91 (0.80–0.96) |
| Lanspa et al, 2013 | cIVC ≥15% | 1.00 | 0.67 | 0.62 | 1.00 | 0.83 (0.58-1.00) |
| | SVV≥17% | 0.60 | 1.00 | 1.00 | 0.82 | 0.92 (0.73-1.00) |
| | AoVV≥25% | 0.75 | 0.67 | 0.50 | 0.85 | 0.67 (0.32-1.00) |
| Muller et al, 2012 | cIVC of 40% | 0.70 | 0.80 | 0.72 | 0.83 | 0.77 (0.60- 0.88) |
| | E wave velocity of 0.7 | 0.67 | 0.90 | 0.84 | 0.83 | 0.83 (0.68-0.93) |
| Préau et al, 2012 | ΔPP ≥10% | 0.60 | 1.00 | 1.00 | 0.76 | 0.71 ± 0.12 |
| | ΔPPdim ≥12% | 0.90 | 1.00 | 1.00 | 0.93 | 0.95 ± 0.05 |
| | ΔVF ≥10% | 0.60 | 1.00 | 1.00 | 0.76 | 0.74 ± 0.11 |
| | ΔVFdim ≥12% | 0.90 | 1.00 | 1.00 | 0.93 | 0.95 ± 0.05 |
| Préau et al, 2010 | ΔSV-PLR ≥10% | 0.86 | 0.90 | 0.86 | 0.90 | 0.94 ± 0.04 |
| | ΔPP-PLR ≥9% | 0.79 | 0.85 | 0.79 | 0.85 | 0.86 ± 0.08 |
| | ΔVF-PLR ≥8% | 0.86 | 0.80 | 0.75 | 0.89 | 0.93 ± 0.04 |

PPV: positive predictive value. NPV: negative predictive value. cIVC: inferior vena cava collapsibility index. IVCmax: inferior vena cava maximum diameter. ΔCO-PLR: change in cardiac output between baseline and after passive leg raising. PLR: passive leg raising. $PPV_{FIB}$: pulse pressure variation during forced inspiratory breathing. SVV: stroke volume variation. AoVV: aortic velocity variation. ΔPP: pulse pressure variation. ΔPPdim: deep inspiration maneuver-induced change in pulse pressure. ΔVF: respiratory change in the velocity peak of femoral artery flow. ΔVFdim: deep inspiration maneuver-induced change in velocity peak of femoral artery flow. ΔSV-PLR: passive leg raising induced-change in stroke volume. ΔPP-PLR: passive leg raising induced-change in radial pulse pressure. ΔVF-PLR: passive leg raising induced-change in the peak velocity of femoral artery flow.

**Fig. 6 (Abstract P16).** Performance of available methods to assess fluid responsiveness in spontaneously breathing patients

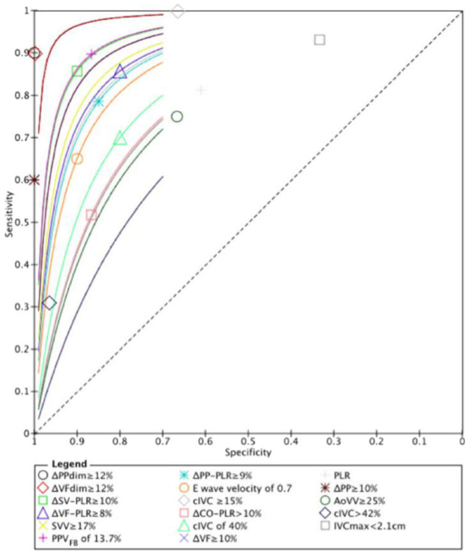

**Fig. 7 (Abstract P16).** ROC curves of methods to assess fluid responsiveness in spontaneous breathing patients

### P17

### Agreement between muscle tissue hemoglobin index measured with near-infrared spectroscopy and blood hemoglobin levels in critically ill patients

Renato Carneiro de Freitas Chaves, Roberto Rabello Filho, Ary Serpa Neto, Murillo Santucci Cesar Assunção, Denise Carnieli Cazati, Letícia Bagatini Meneghini, Thiago Domingos Corrêa
HIAE - Hospital Israelita Albert Einstein, São Paulo, SP, Brazil
*Critical Care* 2017, **21(Suppl 2):**P17

**Introduction:** The reliability of tissue hemoglobin index (THI) provided by near-infrared spectroscopy (NIRS) in critically ill patients is not defined [1]. Objective: Our objective was to assess the agreement between THI and blood hemoglobin level measured at the central lab in critically ill patients with and without circulatory shock.
**Methods:** This prospective single-center study was approved by the ethics committee of Hospital Israelita Albert Einstein. Written informed consent was obtained from each participant. Thirty-eight critically ill patients admitted to the ICU within 24 h were included in this study. Tissue (skeletal muscle) hemoglobin index was measured at the thenar eminence using an InSpectra StO2 Tissue Oxygenation Monitor (model 650; Hutchinson Technology, Hutchinson, MN, USA) using a 15 mm probe [1]. Blood hemoglobin levels (HbLab) were collected as part of the care of critical ill patients and analyzed with Sysmex XN-9000® (Sysmex, Inc. Kobe Japan). Agreement (Bland and Altman method) and Pearson correlation between THI and HbLab were accessed in all patients and in pooled patients accordingly to presence of circulatory shock.
**Results:** Thirty-eight critically ill patients [median IQR; 59 (46-70) years] were included in this analysis. Of these, 20 (52.6%) had circulatory shock (80% septic shock and 20% cardiogenic shock). Tissue hemoglobin index [median (IQR); 12.3 (9.9-14.3) a.u.] was higher than HbLab [10.3 (9.0-11.8) g/dL], mean difference: 1.53; 95%CI: 0.48 to 2.58; p = 0.005. Agreement (mean error, 1.53, IC95%, 0.52 to 2.54; Fig. 8, panel A) and correlation (r = 1.04, p = 0.533; Fig. 8, panel B) between THI and HbLab were poor and not affected by presence of shock (Fig. 8, panels E-F).
**Conclusions:** Agreement between THI and blood hemoglobin concentration is low and the measurement is not interchangeable. Consequently, THI should not be used in clinical practice with the purpose of predicting blood hemoglobin concentration.

### Reference

1. Creteur J, et. al. Near-infrared spectroscopy technique to evaluate the effects of red blood cell transfusion on tissue oxygenation. Critical care. 2009;13 Suppl 5:S11.

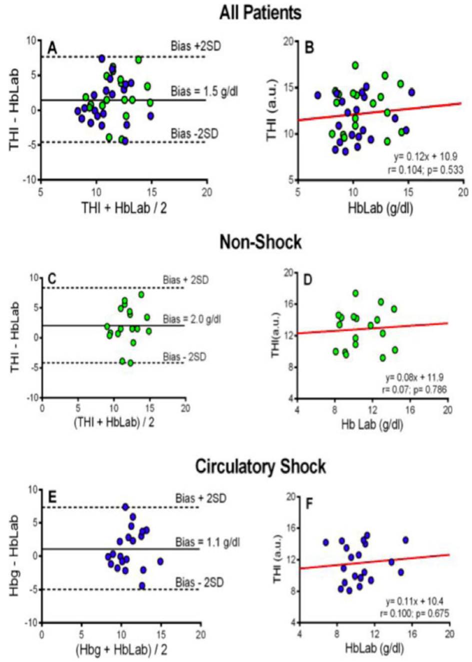

**Fig. 8 (Abstract P17).** Agreement and correlation between tissue hemoglobin index determined with near-infrared spectroscopy (THI) and blood hemoglobin levels measured at central lab (HbLab) in critically ill patients

### Infection

### P18

### Clinical approach to poisonous plants' intoxication

Yuri Cavalcanti Albuquerque Tenorio, Amauri Clemente da Rocha, Helena Moraes da Silva
UNCISAL - Universidade Estadual de Ciências da Saúde de Alagoas, Maceió, AL, Brasil
*Critical Care* 2017, **21(Suppl 2):**P18

**Introduction:** Plants produce many metabolites and some are used to defend themselves from predators; consequently, some species are toxic to humans. There are three main plant poisoning causes: unintentional intoxication, intentional intoxication (homicides and suicides) and poisoning due to abuse of plant material (medicinal use) [1]. Children are the population at risk to suffer unintentional intoxication from poisonous plants, especially in schools with ornamental plants [2].
**Objective:** Facilitate the clinical approach to poisonous plants intoxication, separating the clinical features into syndromes, with the perspective treatment to each.
**Methods:** This paper is a literature review discussing different mechanisms of action, etiology, clinical features and treatment of the most common ornamental poisonous plants in Brazil (Fig. 9).
**Results:** The symptoms of plant intoxication were divided into five syndromes (Table 3). The first is the skin irritation [3] and the second is the eye irritation [4]. The treatment of both syndromes is hygiene and symptomatic drugs. The third is the gastrointestinal irritation (GII) without systemic manifestations (SM), that causes abdominal cramps, vomiting, diarrhea, and dehydration and hepatotoxicity in severe cases. The treatment consists in avoiding gastric lavage or emesis, stimulating demulcent liquids and using symptomatic drugs[3]. The fourth is the syndrome of GII with SM, that has the same GI symptoms, with addition to fever, dyspnea, arrhythmia, cardiac arrest and kidney failure. The treatment is similar to the GII without SM, but with an early correction to the hydroelectrolytic disorders[1,6]. The last is the cardioneurotoxicity, that simulates intoxication to atropine or digitalis, or causes cellular anoxia by cyanuric acid. Cardiovascular symptoms are tachycardia or bradycardia, hypotension, arrhythmias and the neurological symptoms are headache, seizures, coma. Respiratory disorders and renal failure can be associated [5,6]. See the treatment on (Table 3).

**Conclusions:** Symptoms of plants intoxication vary from a simple pruritus to coma and death; consequently, physicians must be aware of the different clinical features from the big variety of poisonous plants, and know how to manage each situation.

### References

1. Beyer J, et al. Analysis of toxic alkaloids in body samples. Foren Scien Internat, 2009. 185, 1-9.
2. SINITOX. Dados de intoxicação, Brasil, 2013. Retrieved from: http://sinitox.icict.fiocruz.br/dados-nacionais in 2/25/2017, at 10:04 AM
3. Ernst M, et al. Global medicinal uses of Euphorbia L. (Euphorbiaceae). Jour Ethnopharm. 2015, 176, 90-101.
4. Dutta J, et al. Penetrating keratoplasty restoring vision in an unusual case of corneal opacity following exposure to Euphorbia latex. Trop Doct. 2015. 45(4), 239-40.
5. Kaland M, et al. Toxalbumin exposures: 12 years' experience of U.S. poison centers. Toxicon. 2015. 1(99), 125-9.
6. Diaz J. Poisoning by Herbs and Plants: Rapid Toxidromic Classification and Diagnosis. Wilde & Environ Med, 2016. 27, 136-152.

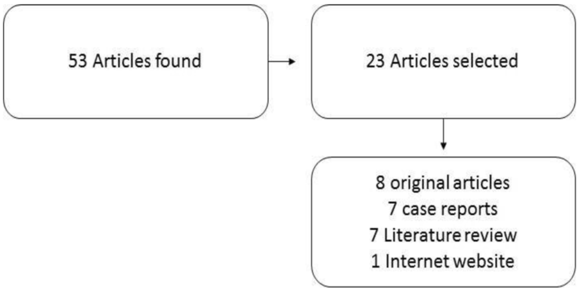

**Fig. 9 (Abstract P18).** Selection of articles to the literature review

**Table 3 (Abstract P18).** Clinical features of poisonous plants intoxication divided into different syndromes with each treatment. Results from the literature review

| Syndrome | Clinical features | Treatment | Active substances | Scientific name | Popular name in Brazil | Authors |
|---|---|---|---|---|---|---|
| Syndrome of skin irritation | • Irritation of the skin and mucous<br>• Hyperemia or the appearance of vesicles, blisters and pustules<br>• Pruritus and pain with a sensation of burning skin | • Hygienic care, lavage with potassium permanganate 1: 10,000<br>• Ointments with corticosteroids<br>• Antihistamines (such as systemic desloratadine 5 mg tablets once daily)<br>• In severe cases the corticoids can be used, such as topical methylprednisolone aceponate 0.1% | Irritating latex sap | *Euphorbia milii L.*<br>*Euphorbia pulcherrima Willd*<br>*Euphorbia tirucalli L.* | Coroa-de-Cristo<br>Bico-de-papagaio<br>Avelós | • Ernst, et al., 2015<br>• Darlenski, Kazandjieva, & Tsankov, 2014<br>• Kesler, 2009<br>• Wilken & Schempp, 2005<br>• Kaland, Klein-Schwartz, & Anderson, 2015 |
| | | | Toxalbumin | *Jatropha curcas L.*<br>*Ricinus communis L.*<br>*Datura suaveolens L.* | Pinhão-roxo<br>Mamona<br>Saia-branca | |
| | | | Histamine, acetylcholine and serotonin | *Fleurya aestuans L.* | Urtiga | |
| Syndrome of eye irritation | • Keratoconjunctivitis<br>• Superficial epithelial defects<br>• mild to moderate corneal edema<br>• Photophobia and tearing<br>• Lesions in the cornea<br>• Anterior uveitis<br>• Descemet membrane folds<br>• Raised intraocular pressure and rarely corneal opacity in severe untreated cases | • Delayed washing with running water<br>• Antiseptic and analgesic eye drops, if necessary<br>• Obtain medical attention from ophthalmologist if irritation persists | Irritating latex | *Euphorbia milii L.*<br>*Euphorbia pulcherrima Willd*<br>*Euphorbia tirucalli L.* | Coroa-de-Cristo<br>Bico-de-papagaio<br>Avelós | • Dutta, et al., 2015<br>• Froberg, Ibrahim, & Furbee, 2007 |
| | | | Toxalbumin | *Jatropha curcas L.*<br>*Ricinus communis L.*<br>*Datura suaveolens L.* | Pinhão-roxo<br>Mamona<br>Saia-branca | |

**Table 3 (Abstract P18).** Clinical features of poisonous plants intoxication divided into different syndromes with each treatment. Results from the literature review *(Continued)*

| | | | | | | |
|---|---|---|---|---|---|---|
| | | | Histamine, acetylcholine and serotonin | *Fleurya aestuans L.* | Urtiga | |
| | | | Cardiotoxic glycosides | *Nerium oleander L.*<br>*Digitalis purpurea L.*<br>*Thevetia peruviana Schum*<br>*Asclepias curassavica L.* | Espirradeira<br>Dedaleira<br>Chapéu-de-Napoleão<br>Oficial de sala | |
| Syndrome of gastrointestinal irritation without systemic manifestation | • Erythema and edema (swelling) of the lips, tongue, palate and pharynx<br>• Burning pain<br>• Abdominal cramps, nausea, vomiting and diarrhea<br>• Sialorrhea, dysphagia and asphyxia | • Avoid gastric lavage or emesis<br>• Stimulate demulcent liquids (milk, egg white, olive oil, mouthwash with aluminum hydroxide)<br>• Symptomatic drugs: analgesics, antispasmodics, antihistamines<br>• Corticosteroids in severe cases | Calcium oxalate | *Zantedeschia aethiopica Spreng*<br>*Dieffenbachia picta Schott*<br>*Caladium bicolor Vent*<br>*Colocasia antiquorum Schott*<br>*Rollinia leptopetala* | Copo-de-leite<br>Comigo-ninguém-pode<br>Tinhorão<br>Taioba-brava<br>Banana-de-macaco | • Ernst, et al., 2015<br>• Froberg, Ibrahim, & Furbee, 2007 |
| | | | Irritating latex | *Euphorbia milii L.*<br>*Euphorbia pulcherrima Willd*<br>*Euphorbia tirucalli L.* | Coroa-de-Cristo<br>Bico-de-papagaio<br>Avelós | |
| Syndrome of gastrointestinal irritation with systemic manifestation | • GII symptoms: abdominal pain, nausea, vomiting, severe cramps, sometimes bloody diarrhea<br>• Systemic manifestations: hypotension, dyspnea, arrhythmia, and cardiac arrest<br>• Evolution for severe dehydration, shock, hydroelectrolytic disorders, torpor, hyporeflexia, and coma. Kidney failure may occur | • Antispasmodics<br>• Antiemetics, possibly antidiarrheals.<br>• Early correction of hydroelectrolytic disorders must be done | Toxalbumin | *Jatropha curcas L.*<br>*Ricinus communis L.*<br>*Datura suaveolens L.* | Pinhão-roxo<br>Mamona<br>Saia-branca | • Kaland, Klein-Schwartz, & Anderson, 2015<br>• Thornton, Darracq, & Lo, 2014<br>• Froberg, Ibrahim, & Furbee, 2007) |
| Cardioneurotoxic syndrome | Atropine-like intoxication:<br>• Rapid onset of nausea and vomiting<br>• Fever<br>• *Cardiovascular symptoms*: tachycardia, mydriasis, peripheral vasodilation, dry and reddened skin, dry mucous membranes, facial flushing<br>• *Neurologic symptoms*: behavioral disorders, hallucinations and delusions, psychomotor agitation<br>• In severe cases occurs neurological depression and coma, cardiovascular, respiratory and death disorders | • Gastric emptying with gastric lavage (in good time) with water, potassium permanganate or 4% tannic acid<br>• Supportive / symptomatic treatment<br>• Treat hyperthermia with physical measures<br>• Avoid sedatives in more severe cases | Daturine alkaloid | *Datura metel*<br>*Datura stramonium L.* | Saia roxa<br>Estramônio | • Nikolau, et al., 2012<br>• Liu, et al., 2011<br>• Beyer, Drummer, & Maurer, 2009<br>• Gaillard & Pepin, 1999<br>• Eliot, 2002 |
| | | | Saponins and neurotoxic alkaloids | *Melia azedarach L.* | Lírio | |
| | Digitalis-like intoxication:<br>• The ingestion causes pain and burning, followed by sialorrhea, nausea, vomiting, abdominal cramps, and diarrhea<br>• Neurological symptoms: headache, dizziness, mental confusion and visual disturbances<br>• Cardiovascular disorders: arrhythmias, bradycardia, and hypotension | • Supportive treatment using antispasmodics, antiemetics, mucosal protectors and intestinal adsorbents<br>• Antiarrhythmics in rhythm disturbances<br>• Special attention to hydroelectrolytic disorders | Cardiotoxic glycosides | *Nerium oleander L.*<br>*Digitalis purpurea L.*<br>*Thevetia peruviana Schum*<br>*Asclepias curassavica L.* | Espirradeira<br>Dedaleira<br>Chapéu-de-Napoleão<br>Oficial de sala | • Diederich, Muller, & Cerella, 2017<br>• Diaz, 2016<br>• assop, Donovan, Cohee, Mabe, & Wedam, 2014<br>• Oerther, 2011<br>• Botha & Penrith, 2008 |
| | Cellular anoxia caused by cyanuric acid:<br>• *Gastrointestinal disorders*: nausea, vomiting, abdominal cramps, diarrhea, that might lead to metabolic acidosis<br>• *Neurological manifestations*: drowsiness, numbness, seizures and coma<br>• *Typical triad*: opisthotonus, trismas and mydriasis<br>• *Respiratory disorders*: dyspnea, apnea, secretions, cyanosis<br>• *Cardiocirculatory disorders*: hypotension in the final phase | • Early treatment, with gastric emptying<br>• Supportive drugs<br>• Amyl nitrite<br>• Sodium Nitrite 3% in adults<br>• Sodium Hyposulfite 25% 1 ml/kg in children | Cyanogenic Glycosides | *Manihot utilissima Pohl.*<br>*Prunus sphaerocarpa SW* | Mandioca-brava<br>Coração de Negro or Pessegueiro Bravo | • Diaz, 2016<br>• Cobilinschi et al, 2016<br>• Abdullah et al, 2013<br>• Gaillard & Pepin, 1999 |

### P19

### Correlation between mass and volume of collected blood with positivity of blood cultures

Lariessa Neves, Alexandre R Marra, Thiago Z S Camargo, Maura C Santos, Patricia C Silva, Natalia A Moura, Elivane S Victor, Jacyr Pasternak, Oscar F Pavão, Michael B Edmond, Marines D V Martino, Flavia Zulin
HIAE - Hospital Israelita Albert Einstein,São Paulo, SP, Brasil
*Critical Care* 2017, **21(Suppl 2):**P19

This abstract is not included here as it has already been published [1].

#### References

1. BMC Research Notes 20158:383 DOI: 10.1186/s13104-015-1365-8

### P20

### Incidence of healthcare-associated infections in adult burn patients and antimicrobial resistance pattern of microorganisms isolated

Elisangela Zampar, Alexandre Narciso, Renan Petinelli, Mariani Garcia, Lucienne Cardoso, Claudia Carrilho, Cintia Grion
UEL - Universidade Estadual de Londrina, Londrina, Paraná, Brasil
*Critical Care* 2017, **21(Suppl 2):**P20

**Introduction:** In Burn Treatment Centers, healthcare-associated infections are responsible for 75% to 80% of deaths. There is a greater incidence of sepsis in patients with burns when compared to other types of trauma. Infection can lead to deterioration in wound healing and serious systemic complications.
**Objective:** The objective of this study was to analyze the incidence of hospital acquired infections in burn patients, and to determine the principal infection sites and the sensitivity profile of the microorganisms to antimicrobials.
**Methods:** This is a retrospective cohort study in a specialized center for the treatment of burns from January 2009 to December 2013. The sample consisted of 404 patients, divided into two groups: the first group of 142 patients without infection and the second group of 262 patients who acquired healthcare-associated infections.
**Results:** There was a predominance of males in both groups, and the mean age of the patients without infection was 37 years (standard deviation SD 14.89) and of the patients with healthcare-associated infections 38 years (SD 15.78). Of the 523 infections observed in this study, the most frequent sites were pneumonia with 216 (41%) cases and urinary tract infections with 137 (26%) episodes. The pathogens identified were Acinetobacter baumannii 93 (40%), Pseudomonas aeruginosa 50 (21%) and Klebsiella (pneumoniae/oxytoca) 23 (10%) and were resistant to most common antimicrobials. In the study population, no pathogens resistant to vancomycin were found.
**Conclusions:** The present study describes high rates of infection in burn victims. The most frequent infections were pneumonia, followed by urinary tract infections caused respectively by non-fermenting bacteria with a high frequency of antimicrobial resistance.

#### References

1. Guggenheim M, Zbinden R, Handschin AE, Gohritz A, Altintas MA, Giovanoli P. Changes in bacterial isolates from burn wounds and their antibiograms: a 20-year study (1986-2005). Burns: journal of the International Society for Burn Injuries. 2009;35(4):553-60.
2. Santucci SG, Gobara S, Santos CR, Fontana C, Levin AS. Infections in a burn intensive care unit: experience of seven years. The Journal of hospital infection. 2003;53(1):6-13.

### P21

### Bacteremia in patients admitted to a private tertiary hospital

Lanara Alves Pereira[1], Andréia Pardini[1], Flávia Fernandes Manfredi Freitas[1], Manuella Carvalho Feitosa[2], Marta Maria Alves Pereira[3]
[1]HIAE - Hospital Israelita Albert Einstein, São Paulo, SP, Brasil; [2]UFRR - Universidade Federal de Roraima, Boa Vista, RR, Brasil; [3]UFPI - Universidade Federal do Piauí, Teresina, PI, Brasil
*Critical Care* 2017, **21(Suppl 2):**P21

**Introduction:** Bacteremia, also called bloodstream infection (BSI), refers to the presence of bacteria in the bloodstream and it is conceptually classified into primary and secondary bacteremia. In the hospital environment, the BSI represents a very significant number and it is usually indicative of severe events, with lethality attributed around 30-50%[1,2]. Included in the scope of hospital infections, we can also highlight the Health Care-Associated Infection (HCAI), which are among the five leading causes of death in the world.
**Objective:** The objectives of this study were to evaluate the occurrence of bacteremia, classify it as primary or secondary, evaluate the associated factors with it and their respective clinical manifestations, in addition to characterize the clinical profile of patients hospitalized in a tertiary care hospital.
**Methods:** Descriptive, quantitative, retrospective study; It was carried out in the department of critical care of a large private hospital in the state of São Paulo - between July 2016 and January 2017. The Chi-square test and the Fisher's exact test were used to perform the clinical correlations. The T-Student test (for the independent groups) and the Mann-Whitney test were also used.
**Results:** The total sample size was 392. The female patients were the majority (77,5%). In the studied groups, the Simplified Acute Physiology Score (SAPS 3) and high mortality risk were observed. These results were higher in the group that did not present bacteremia (mean: 54,5); 25,0% of the infections presented in patients with bacteremia were associated with medical devices. The frequency of primary bacteremia was higher (85,0%) than secondary bacteremia. There was an association between the occurrence of BSI and infections related to the use of devices ($p = 0{,}000$), with a very high prevalence ratio (34,4%). It was demonstrated by logistic regression, that "BSI", "infectious diagnoses" and "endocrine comorbidities" have a significant influence on the occurrence of bacteremia, according to the Wald test, with values of 0,033, 0,039 and 0,045, respectively.
**Conclusions:** Our study showed high severity (SAPS 3 ranged from 96% to 80%) in the study population. Most of the bacteremia detected was related to medical devices, and the frequency of the primary bacteremia was greater than the secondary ones.

#### References

1. Guilarde AO, Turchi MD, Martelli CMT, Primo MGB, Batista LJA. Bacteremias em pacientes internados em hospital universitário. Rev Assoc Med Bras. 2007; 53 (1): 34-8.
2. Garcia OMA, Colmenero CJD. Modelos prognósticos em bacteriemia y sepsis. An Med Interna (Madrid). 2006; 23: 53-55.

### Nephrology

### P22

### Early versus delayed initiation of renal replacement therapy for acute kidney injury– an updated systematic review, meta-analysis, meta-regression and trial sequential analysis of randomized controlled trials

Fabio Tanzillo Moreira[1], Henrique Palomba[1], Renato Carneiro de Freitas Chaves[1], Catherine SC Bouman[2], Marcus Schultz[2,3], Ary Serpa Neto[1]
[1]HIAE - Hospital Israelita Albert Einstein, São Paulo, SP, Brasil; [2]AMC - Academic Medical Center; University of Amsterdam, Amsterdan, The Netherlands; [3]LEICA - Laboratory of Experimental Intensive Care and Anesthesiology, Amsternan, The Netherlands
*Critical Care* 2017, **21(Suppl 2):**P22

**Introduction:** Acute kidney injury (AKI) is a common condition in critically ill patients, associated with higher mortality, particularly if renal replacement therapy (RRT) is required. While life-threatening AKI related symptoms of fluid overload, or metabolic disorders are clear indications for RRT, it is uncertain when to initiate RRT in absence of these conditions.
**Objective:** The aim of the present study was to evaluate whether early initiation of RRT is associate with lower mortality in patients with AKI compared to delayed initiation.
**Methods:** We performed a systematic review and meta-analysis of randomized controlled trials (RCT) comparing early versus delayed initiation of RRT in patients with AKI without life-threatening AKI related symptoms of fluid overload or metabolic disorders. The primary outcome was mortality at the longest follow-up.
**Results:** Six RCTs (1,292 patients) were included (Fig. 10). There was no statistically significant difference between early and delayed initiation of RRT regarding the primary outcome (OR 0.82; 95% CI, 0.48 – 1.42; p = 0.488); there was an increased risk of catheter-related bloodstream infection when RRT was initiated early (OR 1.77; 95% CI, 1.01 – 3.11; p = 0.047) (Fig. 11). The quality of evidence generated by our meta-analysis for the primary outcome was considered low due to the risk of bias of the included studies and the heterogeneity among them.
**Conclusions:** Early initiation of RRT is not associated with improved survival. However, the quality of the current evidence is low and the criteria used for 'early' and 'delayed' initiation of RRT are too heterogenous among studies.

**References**

1. Tolwani A. Continuous renal-replacement therapy for acute kidney injury. N Engl J Med 2012;367:2505-14.
2. Doyle JF, Forni LG. Acute kidney injury: short-term and long-term effects. Crit Care 2016;20:188.
3. Villa G, Ricci Z, Ronco C. Renal Replacement Therapy. Crit Care Clin 201;31:839-48.
4. Kidney International. KDIGO clinical practice guideline for acute kidney injury. Kidney Int Suppl 2012;2:1-138.
5. Palomba H, do Amaral Campos PP, Corrêa TD, et al. Defining and treating acute kidney injury patients in Brazilian intensive care units: Results from a cross-sectional nationwide survey. J Crit Care 2016;34:33-7.
6. Wierstra BT, Kadri S, Alomar S, Burbano X, Barrisford GW, Kao RL. The impact of "early" versus "late" initiation of renal replacement therapy in critical care patients with acute kidney injury: a systematic review and evidence synthesis. Crit Care 2016;20:122.

| Study | Design | Population | Number of Patients | | | Criteria for Initiation of RRT | | Modality | Never Received RRT | |
|---|---|---|---|---|---|---|---|---|---|---|
| | | | Total | Early | Late | Early | Late | | Early | Late |
| Bouman, 2002[11] | RCT, MC | AKI in mixed patients | 106 | 70 | 36 | Within 12 hours after randomization* | Urea > 40 mmol/L or K > 6.5 mmol/L or pulmonary edema | Continuous | 0 (0%) | 6 (17%) |
| Sugahara, 2004[12] | RCT, SC | AKI after cardiac surgery | 28 | 14 | 14 | Urine output < 30 mL/h for three hours | Urine output < 20 mL/h for two hours | Continuous | 0 (0%) | 0 (0%) |
| Jamale, 2013[13] | RCT, SC | AKI in mixed patients | 208 | 102 | 106 | Urea > 70 mg/dL or creatinine > 7 mg/dL | Clinically indicated by the nephrologist | IHD | 9 (8.8%) | 18 (17%) |
| Wald, 2015[14] | RCT, MC | AKI in mixed patients | 100 | 48 | 52 | Within 12 hours after randomization** | K > 6 mmol/L or $HCO_3$ < 10 mmol/L or $PaO_2$ / $FiO_2$ < 200 and pulmonary edema | IHD or Continuous | 0 (0%) | 19 (36.5%) |
| Zarbock, 2016[6] | RCT, SC | AKI in mixed patients | 231 | 112 | 119 | Within 8 hours after diagnosis of stage 2 AKI by KDIGO | Within 12 hours after diagnosis of stage 3 AKI by KDIGO | Continuous | 0 (0%) | 11 (9.2%) |
| Gaudry, 2016[7] | RCT, MC | AKI in mixed patients | 619 | 308 | 311 | Within 6 hours after diagnosis of stage 3 AKI KDIGO | Oliguria or anuria > 72 hours or urea > 112 mg/dL or K > 6 mmol/L or pH < 7.15 or pulmonary edema | IHD or Continuous | 6 (1.9%) | 154 (49.5%) |

**Fig. 10 (Abstract P22).** Characteristics of the studies included

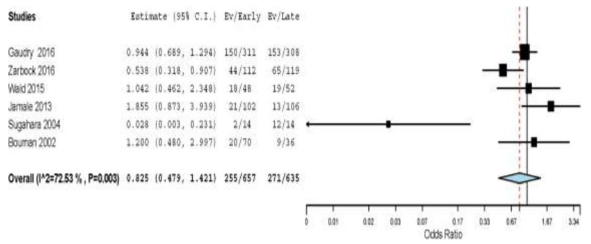

**Fig. 11 (Abstract P22).** See text for description

### P23

### Early versus late initiation of renal replacement therapy in critically ill patients: systematic review and meta-analysis

Bruno Adler Maccagnan Pinheiro Besen, Thiago Gomes Romano, Pedro Vitale Mendes, César Albuquerque Gallo, Fernando Godinho Zampieri, Antônio Paulo Nassar Jr., Marcelo Park
HCFMUSP - Hospital das Clínicas da FMUSP, São Paulo, SP, Brasil

*Critical Care* 2017, **21(Suppl 2):**P23

**Introduction:** Early initiation of renal replacement therapy (RRT) effect on survival and renal recovery of critically ill patients is still uncertain.
**Objective:** We aimed to systematically review current evidence to compare outcomes of early versus late initiation of RRT in critically ill patients.
**Methods:** We searched the Medline (via Pubmed), LILACS, Science Direct and CENTRAL databases from inception until November 2016 for randomized clinical trials (RCT) or observational studies comparing early versus late initiation of RRT in critically ill patients, according to each study's definition, including trials of patients with acute kidney injury (AKI) or not. The primary outcome was the last described mortality. Duration of mechanical ventilation, intensive care unit (ICU) length-of-stay (LOS), hospital LOS and renal function recovery were secondary outcomes. Random-effects meta-analysis and trial sequential analysis (TSA) was used for the primary outcome; and meta-analysis for secondary outcomes.
**Results:** 62 studies were retrieved, including 11 RCTs. There was no difference in mortality between early and late initiation of RRT among RCTs (OR = 0.78; CI 95%, 0.52-1.19; I2 = 63.1% - Fig. 12). TSA of mortality across all RCTs achieved futility boundaries at both 1% (Fig. 13) and 5% type I error rates, although a subgroup analysis of studies including only AKI patients was not conclusive. There was also no difference in time on mechanical ventilation, ICU and hospital LOS, or renal recovery among studies. Early initiation of RRT was associated with reduced mortality among prospective (OR = 0.69; CI 95%, 0.49-0.96; I2 = 85.9%) and retrospective (OR = 0.61; CI 95%, 0.41-0.92; I2 = 90.9%) observational studies, both with substantial heterogeneity. However, subgroup analysis excluding low quality observational studies did not achieve statistical significance.
**Conclusions:** The potential benefit of reduced mortality associated with early initiation of RRT was limited to low quality observational studies, which indicates potential selection bias and confounding by indication. Pooled analysis of randomized trials indicates early initiation of RRT is not associated with lower mortality rates. Furthermore, TSA suggests futility boundaries have been achieved, although the level of confidence in this conclusion is not high, given that this finding did not hold true when considering only patients with AKI.

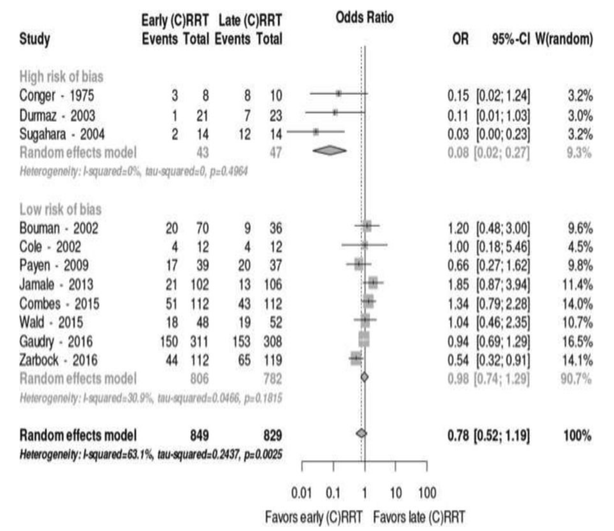

**Fig. 12 (Abstract P23).** Forest plot showing the pooled effects on mortality of all randomized studies comparing early or late initiation of renal replacement therapy in critically ill patients, stratified according to the risk of bias

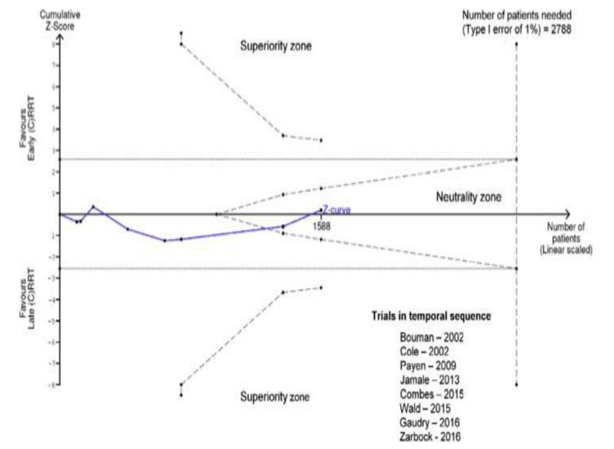

**Fig. 13 (Abstract P23).** Trial sequential analysis of low risk of bias randomized studies comparing the impact on mortality of early vs. late initiation of renal replacement therapy in critically ill patients

### P24

### Predicting AKI reversibility at ICU with renal Doppler resistive index: which factors could influence it?

Raphael A.G. Oliveira[1,2], Pedro Mendes[1,2], Marcelo Park[1], Leandro U. Taniguchi[1,2]
[1]HC FMUSP/SP - Hospital das Clínicas FMUSP/SP (São Paulo, SP, Brasil); [2]HSL - Hospital Sirio-Libanês (São Paulo, SP, Brasil)
*Critical Care* 2017, **21(Suppl 2):**P24

**Introduction:** Renal Doppler resistive index (RI) is a rapid and non-invasive investigative tool used to predict acute kidney injury (AKI) reversibility at ICU setting. However, despite recent meta-analysis had showed a good RI ability to predict persistent AKI, a marked heterogeneity among the studies included was observed. Therefore, it's imperative to discern factors that could influence the RI performance in critically ill patients.
**Objective:** Evaluate the variables that could impact RI in critically ill patients.
**Methods:** Prospective observational study performed at medical-surgical ICU from November 2013 to October 2014. Doppler RI was performed daily until third day after ICU admission, death or RRT requirement. Clinical and blood data were also collected throughout this period. AKI was defined according KDIGO criteria. AKI's reversibility was categorized on transient (normalization of renal function within 48 hours of AKI onset) and persistent (non-resolution of AKI within 48 hours of onset or need for RRT). Linear mixed model was performed between the interested variables (presence of circulatory shock, sepsis, AKI categories, SAPS 3, age, serum chloride, serum lactate and pulse pressure) to evaluate the factors that could influence RI analysis.
**Results:** Eighty-three consecutive patients were included. 65% were male and 50.6% was medical admissions. SAPS 3 were $47 \pm 16$. No differences were observed in age, gender and vasopressor therapy requirement between AKI groups. Sepsis was more common in patients with persistent AKI (14.7%) when compared to other groups ($p = 0.01$). Serum lactate, pulse pressure and serum chloride was not different between groups. Doppler RI was statistically different between no-AKI ($0.64 \pm 0.06$), transient AKI ($0.64 \pm 0.07$) and persistent AKI patients ($0.70 \pm 0.08$, $p < 0.01$). RI also showed a good accuracy to predict persistent AKI on patients with AKI at ICU admission (AUC = 0.78, 95% IC 0.65-0.91 – Fig. 14). Variables associated with RI variations were pulse pressure, lactate, age and AKI category ($p < 0.05$).
**Conclusions:** We observed a goof ability of RI to predict AKI reversibility in critically ill patients. However, some relevant factors might influence RI on this setting.

**Reference**

S. Ninet et al.: Journal of Critical Care 2015; 30: 629–635

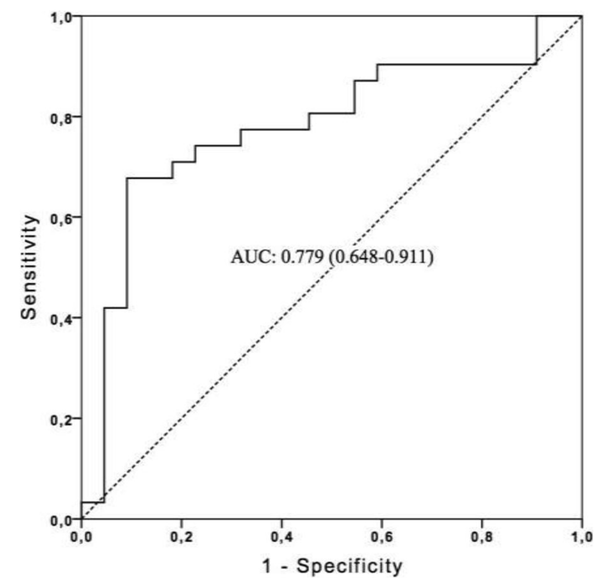

**Fig. 14 (Abstract P24).** ROC curve depicting the ability of RI to detect persistent AKI in patients with AKI at ICU admission

### P25

### Acute Kidney Injury (AKI) related to pregnancy and prognostic factors for hemodialysis in patients treated at Maternal Intensive Care Unit of Hospital Materno Infantil de Brasília(ICU/HMIB)

Dilson Palhares Ferreira[1], Aline Mizusaki Imoto[1], Fábio Ferreira Amorim[1], Amanda Jacomeli Matsuura[1], Adriell Ramalho Santana[1], Jaqueline Lima De Souza[1]
[1]FEPECS - Fundação de Ensino e Pesquisa em Ciências da Saúde, Brasília, Distrito Federal, Brasil; [2]FEPECS - Programa de Pós Graduação em Ciências da Saúde, Brasília, Distrito Federal, Brasil
*Critical Care* 2017, **21(Suppl 2):**P25

**Introduction:** Pregnancy-related acute kidney injury (AKI) is an important cause of maternal morbidity and mortality with rates greater than 30%1. Determining the right moment to start hemodialysis can minimize mortality1.

**Objective:** We attempt to identify the prognostic factors for need for hemodialysis (HD) in pregnancy-related AKI patients admitted to ICU/HMIB.

**Methods:** This was a retrospective analysis of observational data prospectively collected from January 2014 to April 2016 in the maternal ICU/HMIB, Brasilia, Federal District, Brazil. All consecutive patients diagnosed with AKI were included. Patients with a previous diagnosis of renal failure prior to pregnancy were excluded.

**Results:** From a total of 619 patients admitted in the ICU during the study period, pregnancy related AKI was present in 172 cases (27,8%). From the 172 patients with pregnancy related AKI, average age was 29 ± 7 years, gestational age was 32.5 ± 5.7 weeks, APACHE II was 12 ± 7, SOFA score was 3 ± 3, ICU length of stay was 7.6 ± 10.2 days and mortality rate was 8.7% (N = 15). One hundred ten patients were classified as KDIGO 1 (64.0%), 43 as KDIGO 2 (20.9%), and 22 patients as KDIGO 3 (15.1%). Thirteen patients needed HD (7.6%). These patients showed greater APACHE II (26 ± 8 vs. 11 ± 6, P = 0.00), need for invasive mechanical ventilation (IMV 92.3% vs. 24.1%, P = 0.00), use of vasopressors (92.3% vs. 11.9%, P = 0.00) and use of blood products transfusion (53.8% vs. 6.9%). Furthermore, patients required HD had lower mean artery pressure at admission (66 ± 25 vs. 93 ± 21 mmHg, P = 0.00) and use of magnesium sulfate therapy (7.7% vs. 74.2%, P = 0.00) (Fig. 15).

**Conclusions:** In pregnancy related AKI, higher APACHE II, higher SOFA score, lower mean artery pressure, need for IMV, use of vasopressors and use of blood products transfusion were associated with need for HD. Besides, patients that used magnesium sulfate therapy had lower need for HD.

**Reference**

1. Kamal EM et al. RIFLE Classification and Mortality in Obstetric Patients Admitted to the Intensive Care Unit With Acute Kidney Injury: A 3-Year Prospective Study. Reprod Sci 2014;21(10):1281-7.

| Parameter | Hemodialysis (N=13) | Conservative treatment (N=159) | P value |
|---|---|---|---|
| Age, in years (mean±SD) | 30±7 | 29±7 | 0.63 |
| Gestational age, in weeks (mean±SD) | 31±10 | 33±5 | 0.61 |
| APACHE II (mean±SD) | 26±8 | 11±6 | 0.00 |
| Mean artery pressure at admission, in mmHg (mean±SD) | 66±25 | 93±21 | 0.00 |
| Multiparity (N,%) | 9, 69.2 | 94, 59.1 | 0.48 |
| Invasive mechanical ventilation (N,%) | 12, 92.3 | 38, 24.1 | 0.00 |
| Vasoactive drug (N,%) | 12, 92.3 | 19, 11.9 | 0.00 |
| Cesarian delivery (N,%) | 9, 69.2 | 124, 78.0 | 0.47 |
| Blood products transfusion (N,%) | 7, 53.8 | 11, 6.9 | 0.00 |
| Magnesium sulfate therapy (N,%) | 1, 7.7 | 118, 74.2 | 0.00 |

**Fig. 15 (Abstract P25).** Need for therapeutic measures and HD performed in the ICU, in pregnant patients with AKI, admitted to the ICU/HMIB, Jan/2014 to Apr/2016

### Neurology

### P26

### Nursing work time distribution in a neurological intensive care unit

Natalia Nunes Felix, Ana Maria Laus
EERP - USP - Escola de Enfermagem de Ribeirão Preto da Universidade de Sã, Ribeirão Preto, SP, Brasil
*Critical Care* 2017, **21(Suppl 2):**P26

**Introduction:** The knowledge of time spent in nursing activities in patient care makes possible to identify parameters that helps to set up a method of quantifying number of professionals required to assist a unit and is considered management tool of health assistance and human resources.

**Objective:** Identify and analyze nursing work time distribution of a neurological intensive care unit.

**Methods:** It is a quantitative, descriptive, observational and cross-sectional study, developed in the neurological intensive care unit of a large high-complexity hospital in the city of São Paulo. This research considers nursing activities and interventions identified for intensive care units{1}, in according to the standardized language system Nursing Intervention Classification NIC and classified as direct and indirect care interventions, associated activities and personal time. Data were collected using the work sampling method, by means of the direct observation of the activities and interventions performed by the nursing professionals every 10 minutes, during 24 hours, in January 2014.

**Results:** 96 hours data were collected , 10,656 samples of interventions and activities performed by the nursing professionals were obtained Documentation (22.9%), were the most prevalent intervention followed by Medications task (5.9%), Bath (5.4%) and Vital Signs Monitoring (4.4%).Among registered nurses and technicians Documentation is the most frequent for both of them 29,1% and 21,6%. The nursing staff spent 42.1% of their time in direct care interventions 37.1% in indirect care 16.6% in personal activities and 4.2% in associated activities. Registered Nurses spent 55.4% of their time in indirect care interventions, 20.5% in direct care. 16.4% in personal activities and 7.7% in associated activities. Nursing technicians expended more working time in direct care (46.6%), followed by indirect care (33.2%), personal activities (16.6%) and associated activities (3.5%). The mean productivity of the nursing staff reached 83.4%, which is considered excellent as per the literature.

**Conclusions:** The present study provides an objective understanding of the working process of the nursing staff, in neurological intensive care unit and evidences new perspectives of research that support the planning of the nursing staff in critical care units.

**Reference**

1. Mello MC. Carga de trabalho de enfermagem: indicadores de tempo em unidades de clínica médica, cirúrgica e terapia intensiva adulto [tese]. São Paulo: Universidade de São Paulo, Escola de Enfermagem; 2011.

### P27

### Thrombolysis with intravenous alteplase in ischemic stroke with support from the neurologist telemedicine at a secondary public hospital in Sao Paulo experience

Patricia Bulgarelli[1], Carlos Alberto Abreu[1,2], Julio Pereira[1], Lizzie Erthal De Burgo[1], Renata Morbeck[2], Ana Helena Vicente Andrade[1]
[1]HMBOIMIRIM - Hospital Municipal Moyses Deutsch, Sao Paulo, SP, Brasil; [2]HIAE - Hospital Israelita Albert Einstein, Sao Paulo, SP, Brasil
*Critical Care* 2017, **21(Suppl 2):**P27

**Introduction:** Hospital Moyses Deutsch, in the southern city of São Paulo, is indicated for the use of rtPA intravenously in patients with ischemic stroke frame according to the protocol established by the service since 2011. It is necessary to ask neurological evaluation the distance trough of the Hospital Israelita Albert Einstein Telemedicine.
Objective: Demonstrate experience the use of intravenous alteplase clinical practice with Neurologist Telemedicine support, checking the efficiencies and procedure safety.

**Methods:** Retrospective study, in all cases of ischemic stroke who received intravenous alteplase, from December 2011 to December 2016. The protocol indicates the use of alteplase for patients with inclusion criteria, the period between the onset of symptoms and hospital admission up to 4.5 hours and no contraindication to the use of thrombolytics, NIHSS (National Institutes of Health Scale course) calculated on admission and 24 hours after thrombolysis. Computed tomography (CT) on admission and after 24 hours. Solicitation evaluation required by neurological Telemedicine Hospital Israelita Albert Einstein shortly after the conclusion of the TC Cranio. The rt-PA dose of 0.9 mg / kg, 10% of the remaining bolus dose continuous infusion over 1 hour to a maximum dose of 90 mg.

**Results:** Alteplase was used in 104 patients with diagnosis of ischemic stroke. Evaluation Neurology Telemedicine trough has been triggered in 89 cases. Time between onset of symptoms and drug administration, 49 patients less than 90 minutes between 38 90 and 180 minutes, 17 patients between 180-360 minutes The average NIHSS at admission was 18, with 66 patients showed a reduction of 5 or more the points NIHSS score within the first 24 hours. 11 patient non-symptomatic intracranial hemorrhage and 9 symptomatic intracranial hemorrhage and 12 deaths during the period.

**Results:** Alteplase was used in 104 patients with diagnosis of stroke ischemic. Evaluation Neurology Telemedicine trough has been triggered in 89 (86%) cases as shown in Fig. 16. Time between onset of symptoms and drug administration, 49 (37%) patients less than 90 minutes between 38 (47%) 90 and 180 minutes and 17 (16%) patients between 180-360 minutes as shown in Fig. 17. The average NIHSS at admission was 18, with 66 patients showed a reduction of 5 or more the points NIHSS score within the first 24 hours as shown in Fig. 18. 11 (8%) patient non-symptomatic intracranial hemorrhage and 9 (6%) symptomatic intracranial hemorrhage and 12 deaths during the period as shown in Fig. 19.

**Conclusions:** All patients receiving alteplase in the recommended time interval and underwent CT Cranio control. Some cases were not triggered by the evaluation of Neurology Telemedicine. There was improvement in NIHSS score similar percentage observed in reference studies. The protocol implementation has been adequate excellent support of Telemedicine Neurology team. Good profitability of time and therapeutic efficacy. The mortality that correlated with the severity of patients and the NIHSS admission. This data ratifies the safety of intravenous alteplase use in the treatment of ischaemic stroke.

### References

1. Jauch EC, Saver JL, Adams HP Jr, et al. Guidelines for the early management of patients with acute ischemic stroke: a guideline for healthcare professionals from the American Heart Association/American Stroke Association.
2. Stroke 2013; 44:870,
3. Mansur AP, Souza MFM, Favarato D, Avakian SD, César LAM, Aldrigui JM, et al.
4. Stroke and ischemic heart disease mortality trends in Brazil from 1979 to 1996. Neuroepidemiology. 2003;22:179-83.
5. A.H.V. Andrade et al. Thrombolysis with intravenous alteplase in ischemic stroke with support from the neurologist telemedicina at a secondary hospital, experience - Municipal Hospital Moyses Deutsch - Sao Paulo Brazil Intensive Care Medicine Experimental 2016, 4(Suppl 1):27

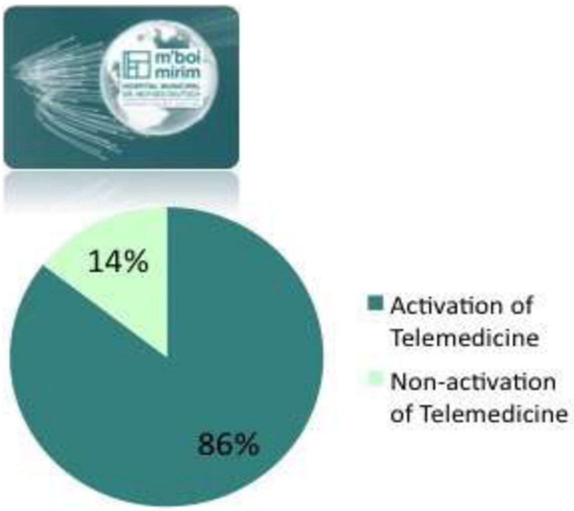

**Fig. 16 (Abstract P27).** Relative frequency of cases that were evaluated by the neurology team through Telemedicine

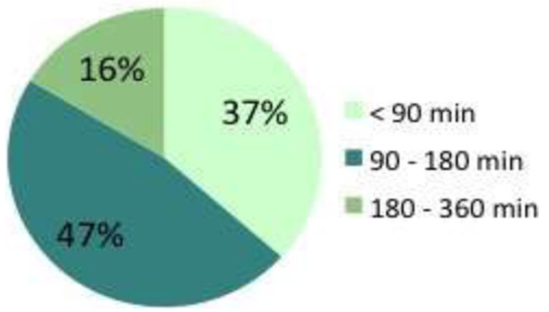

**Fig. 17 (Abstract P27).** Relative frequency of time elapsed for patients to undergo thrombolysis with rt-PA, since hospital admission

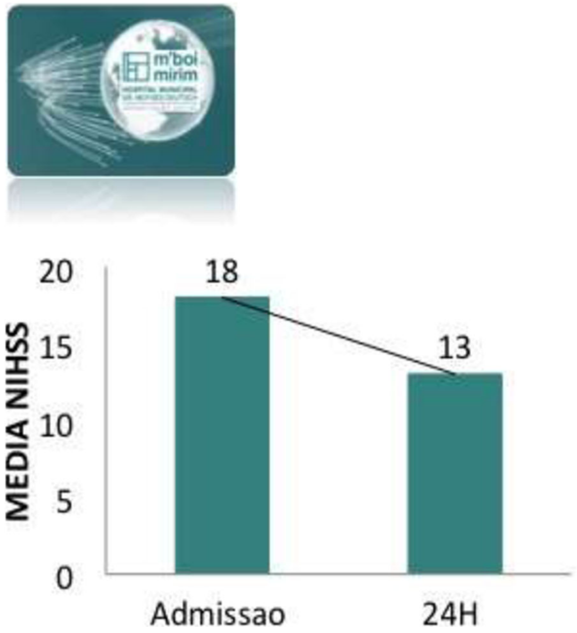

**Fig. 18 (Abstract P27).** Average of the NIHSS scale at admission and 24 hours after the patient underwent thrombolytic therapy

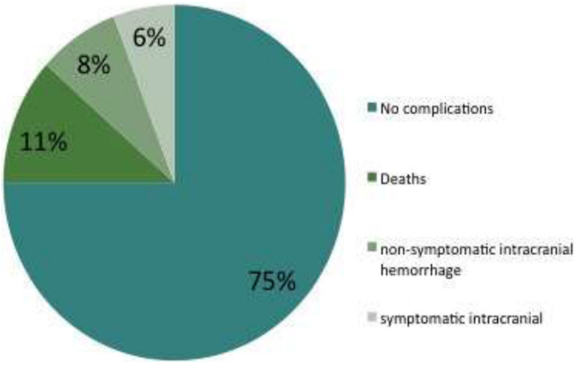

**Fig. 19 (Abstract P27).** Relative frequence of complications and related thrombolytic therapy in patients undergoing chemical thrombolysis with rt-PA

### P28

### Dysphagia in patients affected by Ischemic Stroke

Gabriela Decol Mendonça, Débora D`Agostini Jorge Lisboa, Eliane Lucia Colussi, Gabriela Colussi, Bianca Knapp Ten Caten, Patricia Jost, Angélica Savoldi, Patrícia De Carli Tonial Ghisolfi
UPF - Universidade de Passo Fundo, Passo Fundo, RS, Brasil
*Critical Care* 2017, **21(Suppl 2):**P28

**Introduction:** The presence of the dysphagia symptom is relevant in patients who had a stroke episode. This symptom can cause numerous changes in the patient's clinical condition, so there is a need to obtain data that allow the profile of the patients affected by this swallowing disorder to be pointed out [1]
**Objective:** Check the rate and degree of dysphagia in patients after stroke-I evaluated by the speech therapy team.
**Methods:** It is a longitudinal quantitative qualitative study of retrospective methodology of records from the Hospital da Cidade de Passo Fundo – Rio Grande do Sul. The 152 patient records were analyzed, 89 men and 63 women, in the years 2014 and 2015 with stroke-I. It was included in the study patients with stroke-I diagnosis, of both genders, without age restriction. Did not enter in the search stroke-I patients who developed to stroke-H and other diseases such as Parkinson's, Alzheimer's and cancer patients. The evaluation found demographic and clinical data of the previous patients' history medical records.
**Results:** It was found a higher incidence of dysphagia in older adults with a mean of 70 years old for males. Prevailed mild dysphagia in both genders and oral dietary. From 152 patients, 6 died. In patients post-stroke with dysphagia, there are symptoms such as: absence or delay of the swallowing reflex, increasing the risk of aspiration; Pharyngeal transit levied by absence of intra-oral pressure, alteration of vocal behavior after oral diet suggesting laryngeal penetration, decrease in larynx elevation; Alteration of the labial sphincter, making it difficult to start the swallowing process, reducing tongue control during the oral phase dynamics[1]. Individuals with neurological oropharyngeal dysphagia had a higher frequency of severe grade (46%), followed by mild and moderate degrees (27%, respectively) [2]. The data are in contrast to the study which indicated that, in patients who had neurological oropharyngeal dysphagia, the most present degree was mild, followed by severe and moderate.
**Conclusions:** It was identified a high incidence of patients experiencing dysphagia symptom. Strengthening thus the importance of clinical assessment in patients after stroke-I episode to detect possible swallowing disorder.

#### References

1. Abdulmassih EM, Macedo Filho ED, Santos RS, Jurkiewicz AL. Evolução de pacientes com disfagia orofaríngea em ambiente hospitalar. Art Int Otorrinolaringol. 2009;13(1):55-62. 2. Lacerda NC, Santos SSC. Avaliação nutricional de idosos: um estudo bibliográfico. Rev Rene. 2007; 8(1):60-9.
3. Moraes MAS, Coelho WJP, Castro G, Nemr K. Incidência de disfagia em unidade de terapia intensiva de adultos. Rev. CEFAC. 2006;8(2):171-7.

### Nutrition /Metabolism

### P29

### Protein intake guided by the quadriceps muscle ultrasound in a patient with GBS: case report

Dyaiane Santos, Branca Freitas, Debora Carneiro, Silvia Piovacari, Evandro Figueiredo, Diogo Toledo
HIAE - Hospital Israelita Albert Einstein, São Paulo, SP, Brasil
*Critical Care* 2017, **21(Suppl 2):**P29

**Introduction:** Guillan Barré Syndrome is caracterized by muscular weakness. The intensity may vary from mild weakness to complete tetraplegia with indication to mechanical ventilation which requires hospitalization in an Intensive Care Unit (ICU). Quadriceps muscle ultrasonography represents one of the strategies to evaluate the linear development of muscular mass in acute patients, as it is a non – invasive procedure and highly applicable to bedridden patient1. Nitrogen balance is a non-invasive and accessible technic to evaluate metabolic stress which consists of comparing the difference between ingestion and excretion of nitrogen. The higher the losses the higher the catabolism degree.
**Objective:** Our objective was to evaluate the efficacy of quadriceps muscle ultrasound serial in the patient with GBS in the ICU, to optimize to protein intake and minimize muscle mass loss.
**Methods:** This study obtained written informed consent from participant. Male adult patient, 39 years of age, with GBS, in the ICU from 8/18/2016 to 11/22/2016. Quadriceps muscle ultrasound, nitrogen balance and weight check were performed weekly to adjust protein intake during nutritional therapy. The thickness of the quadriceps musculature was quantified with a portable B-mode ultrasound device. With the patient lying supine, knees extended and relaxed, 2 landmarks on each quadriceps were identified and marked. The muscle thickness was quantified by the use of onscreen calipers and taken as the distance between the upper margin of the femoral bone and the lower boundary of the deep fascia of the rectus femoris. Each landmark was imaged twice and averaged across each leg and then between legs.
**Results:** In the first month at ICU the average protein intake was between 1,6 g to 1,8 g/Kg/day, having an average weight loss of 6,2 kg and nitrogen balance of -14. In the second month the average protein intake was between 2,0 g to 2,5 g/Kg/day, having an average weight loss of 10,3 kg, a decrease of 0,36 mm of quadriceps muscle and nitrogen balance of -7,3. In the third month the average protein intake was 3,5 g/Kg/day, having a stabilized weight, increase of 0,16 mm of quadriceps muscle and nitrogen balance of +3,0. During data collection the motor rehabilitation protocol was kept.
**Conclusions:** Quadriceps Ultrasonography serial measures were efficient and useful to optimize protein intake. This case showed a correlation between negative nitrogen balance and muscle mass loss and between positive nitrogen balance and muscle mass gain assessment by US.

#### Reference

Simpson F, Doig GS. Bedside nutrition evaluation and physical assessment techniques in critical illness. Curr Opin Crit Care 2016, 22:303–307.

### P30

### Bedside Ultrasound Is a Practical Measurement Tool for Assessing Quadriceps Muscle: a pilot study

Diogo Toledo, Branca Freitas, Dyaiane Santos, Debora Carneiro, Rogério Dib, Silvia Piovacari, Ricardo Cordioli, João Manoel Silva Jr
HIAE - Hospital Israelita Albert Einstein, São Paulo, SP, Brasil
*Critical Care* 2017, **21(Suppl 2):**P30

**Introduction:** Bedside ultrasound measurement has previously been used to quantify muscle layer thickness at the quadriceps muscle and some studies about nutritional assessment have showed excellent inter-rater and intra-rater reliability of the technique.
**Objective:** The objectives of this study were to evaluate the intra, inter-reliability and facility of measuring quadriceps muscle layer thickness (QMLT) using bedside ultrasound.
**Methods:** The study was approved by the ethics committee of Hospital Israelita Albert Einstein. A prospective study was carried out for measuring QMLT in two healthy volunteers. The thickness of the quadriceps musculature was quantified with a portable B-mode ultrasound device in volunteers the lying supine, knees extended and relaxed, beside 2 landmarks on each quadriceps were identified. QMLT was calculated by measuring at the border between the lower third and upper two-thirds between the anterior superior iliac spine (ASIS) and the upper pole of the patella, as well as the measurement of the midpoint between the ASIS and the upper pole of the patella. The muscle thickness was quantified using onscreen calipers and taken as the distance between the upper margin of the femoral bone and the lower boundary of the deep fascia of the rectus femoris (Fig. 20). To standardize measurements, an accompanying training with practical lessons and 6 hours' load time were held for team training. Trainer was 1 physician with advanced training in bedside ultrasound (expert). Trainees were comprised of 3 dietitians, 2 physicians, 1

physiotherapist with no prior ultrasound experience. To validate the image collection by ultrasound measurements were performed comparing between expert and all of trainees in different gender volunteers.

**Results:** Overall, 112 images were examined by expert and compared to trainees. The correlation of Person found excellent relation between expert and all of trainees R2 > 0.90 (Fig. 21). The best association was between the expert and dietitians R2 0.99, P < 0.001 and the worst between expert and physicians R2 0.92, P < 0.001. Regarding Bland Altman comparison, the highest percentage of error found between expert and trainees was 5.12% (CI 95% 3.64-12.37) and the lowest was 1.01% (CI 95% 0.72-2.58), in addition the highest bias of the values described was -0,12 ± 0,19 and lowest was -0,01 ± 0,04.

**Conclusions:** The data analyzed showed an excellent correlation of the measures between expert and trainees. Therefore, this exam as a new nutritional assessment tool is feasible and easily applicable for all health professionals.

**Reference**

1. Tillquist et al Bedside Ultrasound Is a Practical and Reliable Measurement Tool for Assessing Quadriceps Muscle Layer Thickness. JPEN J Parenter Enteral Nutr.2014; 38:886-890).

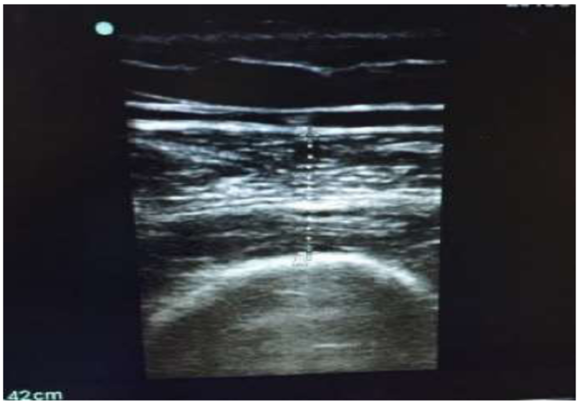

**Fig. 20 (Abstract P30).** See text for description

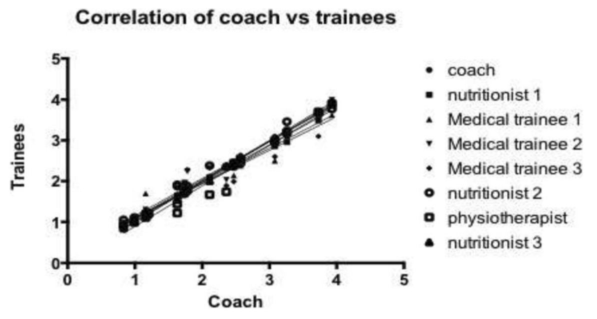

**Fig. 21 (Abstract P30).** See text for description

### P31

### Skeletal muscle mass assessment in critically ill patients by computed tomography

Diogo Toledo[1], Amanda Maria Rosa de Oliveira[2], Jerusa Toloi[2], Amanda Carvalho[2], Carla Prado[3], João Manoel Silva Jr[1]
[1]HIAE - Hospital Israelita Albert Einstein, São Paulo, SP, Brasil; [2]HCB - Hospital do Cancer de Barretos, Barretos, SP, Brasil; [3]UAlberta - University of Alberta, Canada, Edmonton, AB, Canada
*Critical Care* 2017, **21(Suppl 2):**P31

**Introduction:** Typically, in ICU, body composition is assessed through anthropometrics parameters but cannot specifically quantify skeletal muscle (SM). Recent findings shown that computed tomography (CT) have been used to identify critical ill patients with low SM associated with poor clinical outcomes (1).

**Objective:** We have evaluated low muscle mass by CT in comparison to anthropometric data and outcomes in critical ill patients.

**Methods:** The study was approved by the ethics committee of Hospital do Cancer de Barretos. At the period of 1 year, we included patients aged > 18 years, with ICU length of stay more than 72 hours, who performed abdominal CT around 72 hours of admission in the ICU. Demographic data parameters, anthropometric data (weight loss and body mass index-BMI), prognostic index (SAPS 3), complications, ICU length of stay (LOS), hospital mortality and CT abdomen image were collected for analysis. Skeletal muscle and adipose tissue cross-sectional areas were quantified using single-slice CT scans at the third lumbar vertebra (L3). A ROC for hospital mortality was applied to defined the groups of patients with sarcopenia and non-sarcopenia by CT analysis vales. A Cox regression was applied to find independent association between sarcopenia and mortality and a 30 days' survival curve adjusted by SAPS 3 and age was used to check the joint relationship of these features.

**Results:** In the first 72 hours of ICU length of stay, 99 patients were underwent to abdominal CT. The mean age was 61.64 years, 56% male, BMI 24,19 ± 4,49 kg/m2 and hospital mortality was 26%. According to weight loss criteria 43.1% of the patients were classified as malnourished and 19.4% according to BMI. However, BMI values showed no correlation with abdominal CT values for sarcopenia R2 = 0.39, P < 0.001. The cutoff point for determination of sarcopenia by CT was 41.2 cm2 /m2 (sensitivity 70%, specificity 69.5%, AUC 70.3), this was considered as a reference to classify sarcopenic patients (Fig. 22). The diagnosis of sarcopenia by CT with the parameters of nutritional evaluation were correlated with 64.2% by weight loss criteria and only 35.5% by BMI. Otherwise, sarcopenic patients when compared to nonsarcopenic had a worse 30 days survival (Fig. 23) curve adjusted by age and SAPS 3, HR 2.73 CI95% 1.02-7.35 (P = 0.04), higher hospital mortality (41.9% vs 14.6%, P = 0.006) and ICU complication (76.7% vs 52.1%, P = 0.016).

**Conclusions:** The sarcopenia assessed by abdominal CT demonstrated low correlation with malnutrition by BMI and was a risk factor for high hospital mortality, complications, as well as lower 30 days survival in critical ill patients.

**Reference**

Paris M; Mourtzakis, M. Assessment of skeletal muscle mass in critically ill patients: considerations for the utility of computed tomography imaging and ultrasonography. Curr Opin Clin Nutr Metab Care 2016, 19:125–130

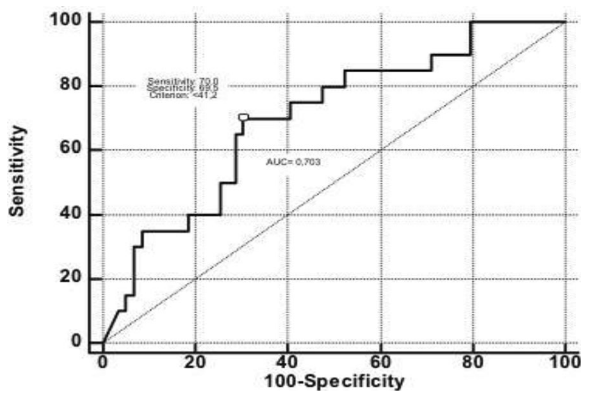

**Fig. 22 (Abstract P31).** See text for description

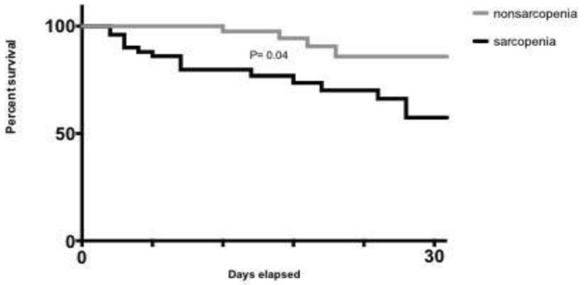

**Fig. 23 (Abstract P31).** See text for description

### P32

### Assessment of body composition by two methods: tomography computed serial imaging and BMI

Ana Paula Noronha Barrére, Branca Freitas, Evandro Figueiredo, Ronaldo Baroni, Silvia Piovacari, Diogo Toledo
HIAE - Hospital Israelita Albert Einstein, São Paulo, SP, Brasil
*Critical Care* 2017, **21(Suppl 2):**P32

**Introduction:** Resources about body composition in cancer have shown the influence in nutritional status during all the trajectory of this disease, in time of survive, in prognostic and tolerance of the antineoplastic treatment. The skeletal muscle mass could be quantify by images in computerized tomography (CT). CT is a technology precise and confiable to avaliate body composition. The use of CT possibilite a direct visualization of structures of transversal areas, muscle mass and adipose tissue (1).
**Objective:** Analyze body composition through serial tomography of a cancer surgical patient and adequacy of the nutrition therapy.
**Methods:** This study obtained written informed consent from participant. Case study of one surgery oncology patient (P.P.), with adenocarcinoma pancreatic cancer, male, 59 years old, from March/2016 to August/ 2016. He was submitted to gastro duodenopancreatectomy and surgery of complications of enteric fistula, in a general hospital of Sao Paulo. Abdominal CT images and BMI obtained during four months of hospitalization and were analyzed, by the SliceOmatic software, at the level of the third lumbar vertebra (L3) on admission and by month. Skeletal muscle mass was assessed by measures of the cross sectional muscle area, from which the skeletal muscle index (SMI) was obtained calculated by the formula cm2/heigh2 (considering normality parameter for male sex 54 cm2/m2). The monitoring was divided into five periods, we calculated the average intake of each period for calories and proteins, considering fasting.
**Results:** According to anthropometric parameters, monitoring patient presented significant weight loss (18.5%) being: initial weight 94 kg, final weight of 76.6 kg. Initial 28.3 kg/m2 BMI and BMI end 23.1 kg/m2. However, according to evaluation of the area by CT and SMI, suggests maintaining skeletal protein reserves in the last 3 periods evaluated. The average values of SMI found were of 36.3 cm2/m2, 37.5 cm2/m2, 36.5 cm2/m2. During this follow-up, the patient received oral nutritional therapy and enteral/parenteral, or being the initial mean values of 19.73 kcal/kg and 0.99 g/kg protein and end of 31.46 kcal/kg and 1.53 grams of protein/kg. In the last 3 periods, the offer was 1.76 average protein, 1.25 and 1.53 grams of protein/kg (Fig. 24).
**Conclusions:** Although nutritional diagnosis by BMI does not reveal malnutrition, assessment of body composition by CT indicated a loss of important muscle mass during hospitalization (sarcopenia). However, the nutritional therapy offered, according to nutritional needs, has contributed to the maintenance of lean mass in recent weeks.

#### Reference

Paris M, Mourtzakis M. Assessment of skeletal muscle mass in critically ill patients: considerations for the utility of computed tomography imaging and ultrasonography. Curr Opin Clin Nutr Metab Care 2016, 19:125–130

| | Períod | Weight (kg) | BMI ($kg/m^2$) | SMI ($cm^2/m^2$) | Diet | Kcal | kcal/kg | PTN/kg |
|---|---|---|---|---|---|---|---|---|
| 03/27 | 1 | 94 | 28,3 | 49,3 | VO | 1854,5 | 19,73 | 0,99 |
| 04/16 | 2 | 91,1 | 27,5 | 43,6 | TPN | 1496,3 | 16,42 | 0,99 |
| 04/27r | 3 | 82,2 | 24,8 | 36,3 | TPN/EN | 2216 | 26,96 | 1,76 |
| 05/20i | 4 | 78,8 | 23,8 | 37,5 | TPN/EN/ VO | 1587,8 | 20,15 | 1,25 |
| 08/18 | 5 | 76,6 | 23,1 | 36,5 | VO | 2410 | 31,46 | 1,53 |

**Fig. 24 (Abstract P32).** See text for description

### P33

### Bedside ultrasound muscle layer thickness assessment of the quadriceps in critically ill patient

Diogo Toledo, Branca Freitas, Debora Carneiro, Dyaiane Santos, Rogério Dib, Evandro Figueiredo, Silvia Piovacari, João Manoel Silva Jr
HIAE - Hospital Israelita Albert Einstein, São Paulo, SP, Brasil
*Critical Care* 2017, **21(Suppl 2):**P33

**Introduction:** Survivors of critical illness experience significant skeletal muscle wasting that may predict clinical outcome. Ultrasound (US) is a noninvasive method that can measure muscle quadriceps muscle layer thickness (QMLT) at the bedside (1,2).
**Objective:** The objective of this study was to evaluate the measuring quadriceps muscle layer thickness using bedside ultrasound in critically ill patient during 7 days follow up.
**Methods:** This was a prospective, single-center study, conducted in a tertiary care hospital. The study was approved by the ethics committee of Hospital Israelita Albert Einstein and written informed consent obtained from each study participant. Patients aged 18 years or older who during their ICU stay required mechanical ventilation were included. Demographic data, anthropometric data, prognostic index (SAPS 3), nutrition risk screening (NRS) and image of US QMLT were collected for analysis. The thickness of the quadriceps musculature was quantified with a portable B-mode ultrasound device. With the patient lying supine, knees extended and relaxed, 2 landmarks on each quadriceps were identified. The underlying tissues were then maximally compressed by the ultrasound probe, and the screen image was frozen. The muscle thickness was quantified using onscreen calipers and taken as the distance between the upper margin of the femoral bone and the lower boundary of the deep fascia of the rectus femoral. Each landmark was imaged and averaged across each leg. Measurements of the first (D1), third (D3) and seventh (D7) days were performed and the percentage of QMLT was displayed for left and right legs.
**Results:** It was enrolled 20 patients underwent to 40 QMLT using US in each leg. The mean age was 57.0 ± 20.2 years, 75% male, BMI 24.6 ± 3.0 kg/m2, SAPS 3 was 51.3 ± 17.4 and NRS 3.2 ± 1.0. Overall, 8.4% (22.0% to -8.9%) of muscle wasting in the right leg and 5.2% (22.6% to -11.0%) in the left leg assessed by the US occurred from the first to the seventh day. In the right leg the median values evaluated by the US were 1.16 cm (0.92 to 1.75) D1, 1.13 cm (0.92 to 1.60) D3, 1.02 cm (0.81 to 1.41) D7; P = 0.005 (Fig. 25). In the left leg, the median values evaluated by the US were 1.23 cm (0.93 to 1.43) D1, 1.16 cm (0.92 to 1.34) D3, 1.13 cm (0.89 to 1.31) D7; P = 0.017 (Fig. 26).
**Conclusions:** The measuring quadriceps muscle layer thickness using ultrasound demonstrated that critical ill patients present muscle wasting daily, and this procedure can be a great differential to identify patients most likely to benefit from enhanced nutritional and rehabilitation support.

**References**

1. Tillquist et al. Bedside Ultrasound Is a Practical and Reliable Measurement Tool for Assessing Quadriceps Muscle Layer Thickness. JPEN J Parenter Enteral Nutr.2014; 38:886-890)
2. Paris M; Mourtzakis, M. Assessment of skeletal muscle mass in critically ill patients: considerations for the utility of computed tomography imaging and ultrasonography. Curr Opin Clin Nutr Metab Care 2016, 19:125–130

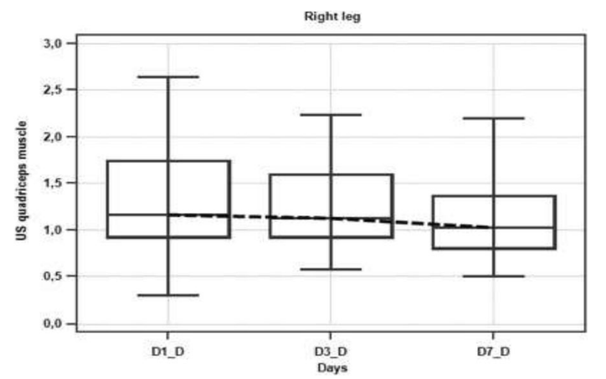

**Fig. 25 (Abstract P33).** See text for description

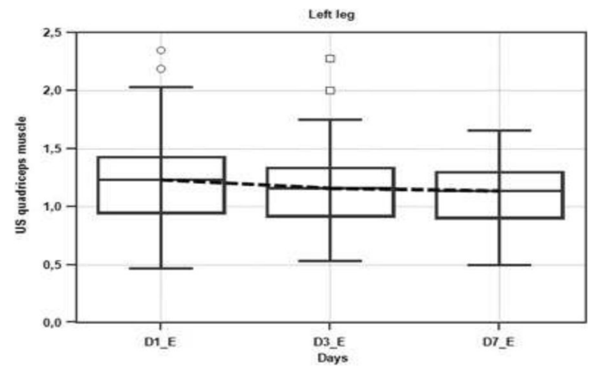

**Fig. 26 (Abstract P33).** See text for description

### P34

### Potential benefits of testosterone administration on ICU-acquired weakness and prolonged mechanical ventilation: a pilot study

Sara Cardoso da Rosa[1], Milena Artifon[1], Ana Carolina Peçanha Antonio[1,2], Silvia Regina Rios Vieira[1]
[1]HCPA - Hospital de Clínicas de Porto Alegre, Porto Alegre, RS, Brasil; [2]HMV - Hospital Moinhos de Vento, Porto Alegre, RS, Brasil
*Critical Care* 2017, **21(Suppl 2):**P34

**Introduction:** Intensive care unit acquired weakness (ICU-AW) is diagnosed in up to 67% among long-term ventilated patients and is associated with a high morbidity and mortality [1]. Testosterone has been shown to ameliorate the catabolism and increase net protein synthesis in severe burn injury [2], chronic obstructive pulmonary disease (COPD)[3] and acquired immunodeficiency syndrome (AIDS) [4].
**Objective:** To assess the potential role of testosterone on ICU-AW recovery and reduction in duration of mechanical ventilation (MV).
**Methods:** A pilot open-label, randomized, controlled trial of testosterone therapy on chronic critically ill adult patients requiring prolonged ventilatory support, defined as endotracheal intubation and MV for more than 14 days, or need for tracheostomy and MV for more than 8 days. Exclusion criteria: arterial or venous thromboembolism in the last 6 months; prior or current history of prostate cancer; refractory shock; severe thrombocytopenia (less than 20,000 platelets per microliter); acute liver failure or decompensate chronic liver disease; overt congestive heart failure; end-of-life care; and patient or surrogate's refusal to participate. Subjects are randomly assigned to receive standard nutritional support and physical therapy, or an additional bi-weekly dose of 200 mg intramuscular testosterone cypionate up to ICU discharge. We use a computer-generated, randomization scheme with varying block sizes ranging from 4 to 8, stratified by gender and coexistence of COPD and/or AIDS; allocation is concealed. Diaphragm ultrasound and Medical Research Council (MRC) score assessment are performed to evaluate muscular weakness. Patients are monitored through complete blood count, lipid panel, renal and hepatic function tests, blood gas analysis, serum free testosterone levels, and caloric and protein intake. Minimum sample foreseen is 15 individuals in each arm. Primary outcome is ventilator-free days.
**Results:** The present research is in initial phase of recruitment, and it will be finished by January 2018. Eleven individuals have been enrolled so far - 7 men and 4 women. In this sample, 7 patients were discharged from the ICU and four died. Currently, only one patient has completed three doses of testosterone, and duration of MV was 90 days. Eight participants have been randomized to intervention with anabolic steroids.
**Conclusions:** High mortality showed by chronic critically ill population is the main obstacle for conduction of present study. Firstly, we aim to better define which type of patient most derives benefit, if any, from anabolic steroids administration. Data obtained from this clinical research may provide great contribution to fill the gap in knowledge of best management of prolonged MV and ICU-AW.

**References**

1. Stanojcic M et al.: Curr Opin Crit Care. 2016 Aug; 22(4):325-31
2. Ferrando AA et al.: Crit Care Med 2001; 29:1936 – 1942
3. Pan L et al.: PLoS One. 2014 Jan 10; 9(1):e84855
4. Blick G et al.: Postgrad Med. 2013 Mar; 125(2):19-29

### Pneumology

### P35

### Validation of the PESI (Pulmonary embolism severity index) score for risk stratification after acute pulmonary embolism in a Brazilian retrospective cohort

Leonardo Soriano, Talita Castro, Kelvin Vilalva, Marcos Borges, Antônio Pazin-Filho, Carlos Miranda
FMRP/USP - Divisão de Emergências Clínicas -FMRP/USP, Ribeirão Preto, São Paulo, Brasil
*Critical Care* 2017, **21(Suppl 2):**P35

**Introduction:** The PESI is a score for risk stratification after acute pulmonary embolism. It was inserted in the 2014 ESC guidelines for guiding the management of these patients.
**Objective:** The objective of this study was validated the PESI score in a Brazilian cohort.
**Methods:** This investigation was a retrospective cohort that included patients hospitalized at the emergency department of our hospital with the diagnosis of acute pulmonary embolism from the period of January 2009 until December 2015. The PESI score (original and simplified version) was applied in all patients with the admission data. The mortality rate in 30 days was the outcome observed.
**Results:** One hundred twenty-three (123) patients were included (57 ± 17 years, 60%female). The mortality rate according to the PESI class (original version) was: Class I (0.80%), Class II (1.62%), Class III (4.87%), Class IV (3.25%), Class V (12.20%); $p < 0{,}0001$. Dichotomized analysis showed: Class I-II (5,8%) vs. Class III-IV-V (34,7%), relative risk (RR): 5.9; 95% confidence interval (CI): 1.88-18.51; $p = 0.0002$ and simplified version was 0 points (3,25%) vs. 1 points (19,51%); RR: 2.38; 95%CI: 0.89-6.38; $p = 0.06$. The survival analysis through the Kaplan-Meier curve showed that the Class I and II had similar curves ($p = 0.59$) and Class III-IV-V had similar curves too ($p = 0.25$) and the comparison of curves of the Class III-IV-V vs. Class I-II showed significantly higher mortality rate at the first group, RR: 7.63; 95%CI: 2.29-25.21; $p = 0.0001$(log-rank-test) justifying this dichotomized analysis (Fig. 27). The simplified version higher or equal 1 points was associated with higher mortality rate compared to 0 points (RR: 2.95; 95%CI: 1.02-8.51; p = 0.03). The dichotomized analysis of the original version showed higher accuracy than simplified version (ROC-curve area: 0.70 (95%CI: 0.62-0.77) vs. 0.60 (95%CI: 0.51-0.67); p = 0.05 for mortality prediction.
**Conclusions:** The PESI score measures adequately the prognostic after acute pulmonary embolism in a Brazilian cohort. The dichotomized analysis of the original version should have higher accuracy than simplified version to predict the 30 days.

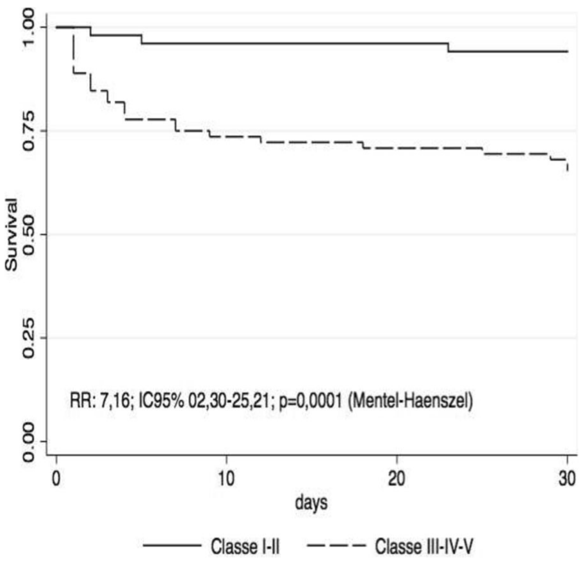

**Fig. 27 (Abstract P35).** Kaplan-Meier curve showing the survival at 30 days in patients with acute pulmonary embolism divided according the PESI score (Class I and II versus Class III, IV and V)

### P36

### Troponin and NT-proBNP levels for prognostic stratification in normotensive patients with acute pulmonary embolism in a Brazilian cohort

Leonardo Soriano, Talita Castro, Kelvin Vilalva, Marcos Borges, Antônio Pazin-Filho, Carlos Miranda
FMRP/USP - Divisão de Emergências Clínicas -FMRP/USP, Ribeirão Preto, São Paulo, Brasil
*Critical Care* 2017, **21(Suppl 2):**P36

**Introduction:** Troponin and N-terminal (NT)-pro natriuretic peptide of type B (BNP) can be used for prognostic stratification after acute pulmonary embolism (APE), mainly in haemodynamically stable patients. Despite this, the appropriate cut-off values of these biomarkers are not clear. Usually, the troponin cut-off value used is the 99th percentile of the curve of normal individuals, which is the same value used for myocardial infarction definition. The NT-proBNP cut-off value used is 600 pg/ml.
**Objective:** The objective was to evaluate the performance of this cut-off value for these biomarkers in patients with APE in a Brazilian cohort.
**Methods:** This is a retrospective cohort that included patients hospitalized at the emergency department of our hospital with APE diagnosis from the period of January 2009 until December 2015. We analyzed only the haemodynamically stable patients in which these biomarkers was quantified through Vidas® Troponin I Ultra and Vidas® NT-proBNP (bioMérieux, France). The 99th percentile for this troponin assay is 0.01 mcg/L. The mortality rate in 30 days was the outcome observed. The levels of biomarkers were expressed in median and interquartile range (75th percentile -25th percentile).
**Results:** Troponin levels were quantified in 75 patients (55 ± 11 years, 40%male), this biomarker was positive in 36 patients (48%), comparing its levels between survivors vs. non-survivors was not observed difference: 0.01(0.08) mcg/L vs. 0.19 (0.34) mcg/L; p = 0.19 and in survival analysis positive troponin was not a predictor of mortality (RR: 1.38; 95%CI: 0.42-4.54; p = 0.58). This troponin cut-off value showed low sensibility 55%; 95%CI: 23-83 e low specificity 53%, 95%CI: 40-66 and low accuracy (curve-ROC area: 0.53; 95%CI: 0.37-0.70) for mortality prediction. NT-proBNP levels were quantified in 64 patients (57 ± 17 years, 34% male), this biomarker was positive in 45 patients (70%), comparing its levels between survivors vs. non-survivors was observed a tendency of higher value in the second group: 1484 (3498) pg/ml vs. 3953 (8418) pg/ml; p = 0.09 and in survival analysis there was a tendency of higher mortality at the group with values higher than 600 pg/ml (RR: 5.12; 95%CI: 0.66-39.7); p = 0.08. This cut-off value of NT-proBNP levels showed adequate sensibility 92%; 95%CI: 62-100, but low specificity 35%; 95%CI: 54-74 and regular accuracy (curve-ROC area: 0.63; 95%CI: 0.52-0.73) for mortality prediction.
**Conclusions:** Despite the underpowered sample, the cut-off value usually used for troponin showed inadequate performance and the cut-off value used for NT-proBNP showed high sensibility however low specificity for mortality prediction in this setting.

### Reference

1. Konstantinides SV, Torbicki A, Agnelli G, Danchin N, et. al. 2014 ESC Guidelines on the diagnosis and management of acute pulmonary embolism. Eur Heart J. 2014; 35(43):3033-69

### P37

### Potentially Modifiable Factors Contributing To Outcome Of Invasively Ventilated Patients Without ARDS – The PRoVENT Study

Flavia Pfeilsticker[1], Ary Serpa Neto[1], Fabienne Simonis[2], Marcelo Gama Abreu[3], Paolo Pelosi[4], Marcus Schultz[2]; for the PROVE Network Investigators
[1]Hiae - Hospital Israelita Albert Einstein, São Paulo, Brazil; [2]AMC - UVA - University Of Amsterdam, Amsterdam, The Netherlands; [3]UHCGC - University Hospital Carl Gustav Carus, Dresden, Germany; [4]UNIGE - Irccs San Martino Ist, Genoa, Italy
*Critical Care* 2017, **21(Suppl 2):**P37

**Introduction:** Mechanical ventilation is increasingly recognized as potentially harmful intervention in patients without the acute respiratory distress syndrome (ARDS), and tidal volume size and airway pressures have been associated with worse outcomes in these patients.
**Objective:** To improve the outcome of invasively ventilated patients without the acute respiratory distress syndrome (ARDS), potentially modifiable factors associated with mortality need to be identified.

**Methods:** The observational 'PRactice of VENTilation in patients without ARDS' (PRoVENT) study was an international, multi–center, prospective cohort study of consecutive ventilated patients in a convenience sample of 119 ICUs from 16 countries across four continents. A pre-specified secondary aim was to examine which factors are associated with outcome. The Lung Injury Prediction Score (LIPS) was used to stratify risk of ARDS. The primary outcome measure was mortality, defined as mortality at hospital discharge or at 90 days after start of mechanical ventilation, whichever occurred first.
**Results:** Nine hundred thirty–five patients were included in the analysis. Patients at risk of ARDS and ventilated with higher maximum airway pressure (Pmax), higher driving pressure levels, higher levels of positive end-expiratory pressure (PEEP), and larger tidal volumes had a higher mortality compared to those receiving ventilation with lower parameters and not at risk of ARDS (Fig. 28). Potentially modifiable factors associated with increased mortality in multivariable analyses included Pmax and pHa (Fig. 29). Non-modifiable factors associated with worsened outcome included older age, presence of COPD, use of immunosuppression and a more dependent condition.
**Conclusions:** Lower maximum airway pressure is associated with improved survival in invasively ventilated patients without ARDS.

**References**
(1) Serpa Neto A, Nagtzaam L, Schultz MJ. Ventilation with lower tidal volumes for critically ill patients without the acute respiratory distress syndrome: a systematic translational review and meta-analysis. Curr Opin Crit Care 2014;20:25–32.
(2) Serpa Neto A, Simonis FD, Barbas CS V, et al. Association between tidal volume size, duration of ventilation, and sedation needs in patients without acute respiratory distress syndrome: an individual patient data meta-analysis. Intensive Care Med 2014; 40:950–7.
(3) Neto AS, Simonis FD, Barbas CS V, et al. Lung-Protective Ventilation With Low Tidal Volumes and the Occurrence of Pulmonary Complications in Patients Without Acute Respiratory Distress Syndrome. Crit Care Med 2015;43:2155–63.
(4) Neto AS, Barbas CS V, Simonis FD, et al. Epidemiological characteristics, practice of ventilation, and clinical outcome in patients at risk of acute respiratory distress syndrome in intensive care units from 16 countries (PRoVENT): an international, multicentre, prospective study. Lancet Respir Med 2016;4:882–93.

| | Odds Ratio (95% CI) | *p* value |
|---|---|---|
| **Clinical characteristics and co-morbidities** | | |
| Age | 1.02 (1.01 – 1.04) | 0.001 |
| Functional status | | |
| Independent | 1 (Reference) | |
| Partially dependent | 2.34 (1.39 – 3.96) | 0.002 |
| Totally dependent | 2.13 (0.97 – 4.69) | 0.060 |
| Hypertension | 0.97 (0.61 – 1.53) | 0.892 |
| Heart failure | 0.99 (0.57 – 1.72) | 0.960 |
| Chronic kidney disease | 1.17 (0.59 – 2.32) | 0.661 |
| COPD | 1.79 (1.00 – 3.21) | 0.049 |
| Immunosuppression | 3.31 (1.61 – 6.81) | 0.001 |
| **Severity of illness** | | |
| SOFA Total | 1.13 (1.05 – 1.22) | 0.002 |
| **Management** | | |
| Use of NIV before intubation | 0.95 (0.46 – 1.94) | 0.885 |
| Maximum airway pressure, $cmH_2O$ | 1.08 (1.02 – 1.13) | 0.016 |
| PEEP, $cmH_2O$ | 0.96 (0.86 – 1.08) | 0.527 |
| $FiO_2$ | 1.00 (0.98 – 1.01) | 0.861 |
| **Laboratory parameters** | | |
| $PaO_2$ / $FiO_2$, mmHg | 1.00 (0.99 – 1.01) | 0.205 |
| $PaCO_2$, mmHg | 0.99 (0.98 – 1.02) | 0.899 |
| pH | 0.09 (0.01 – 0.95) | 0.045 |
| **Vital signs** | | |
| $SpO_2$, % | 0.96 (0.91 – 1.00) | 0.055 |
| Heart rate, bpm | 1.01 (1.00 – 1.02) | 0.006 |
| Mean arterial pressure, mmHg | 0.99 (0.98 – 1.01) | 0.446 |

*CI: confidence interval; NIV: non-invasive ventilation; COPD: chronic obstructive pulmonary disease; SOFA: Sequential Organ Failure Assessment; PEEP: positive end expiratory pressure; $FiO_2$: inspired fraction of oxygen; $SpO_2$: oxygen saturation; BPM: beats per minute*
Mortality is defined as mortality at hospital discharge or at 90 days after start of mechanical ventilation

**Fig. 28 (Abstract P37).** Kaplan-Meier curves for mortality according to the median of maximum airway pressure, tidal volume, PEEP and driving pressure

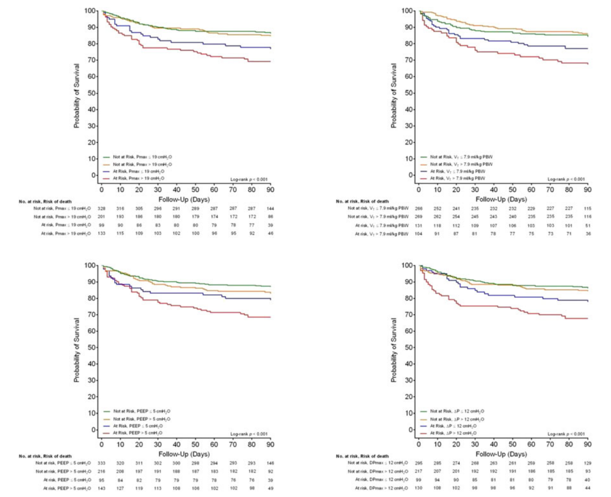

**Fig. 29 (Abstract P37).** Factors associated with mortality in patients without ARDS receiving mechanical ventilation

### P38

### A case of Successful Extracorporeal Oxygenation Membrane (ECMO) after training a multiprofessional ECMO´s Team in Brazil

Filipe Utuari, Marcele Pesavento, Mauriceia Souza, Neide Lucinio
HIAE - Israelita Albert Einstein Hospital, São Paulo, São Paulo, Brazil
*Critical Care* 2017, **21(Suppl 2):**P38

**Introduction:** Adult Respiratory Distress Syndrome (ARDS) is a frequently condition observed in an intensive care unit (ICU), and the use of ECMO as a therapeutic resource has currently good survival rates.1
**Objective:** To report a case of successful ECMO application, after training the Multi-professional ECMO´s Team.
**Methods:** Case Report.
**Results:** A 30-year-old male patient was hospitalized for H1N1 Acute Respiratory Insufficiency. ICU care was required after worsening of the respiratory condition, with signs of severe ARDS. The patient did not respond to non-invasive measures, he was placed in in Mechanical Ventilation (MV) with FiO2 100%. Measures for protective ventilation were initiated, and even with alveolar recruitment, use of electrical impedance tomography and neuroblockers still maintained a gasometric ratio of 70, without improvement of peripheral oxygen saturation (SpO2) 90%, then placed the Patient in Prone position. However, there was no improvement and the ECMO Venovenous was indicated. Additionally, ECMO specialists had recently been trained, this was one of the first cases that occurred after this training. The ECMO being passed to bedside and guided by Transoesophageal Echocardiography. Cannulas of size 18-F were used in Right Jugular Vein and 25-F in Right Femoral Vein without difficulties. After ECMO application, there was improvement in SpO2 to 96%, decrease of FiO2 to 40%, and improvement of gasometric hypoxia. The nursing team was responsible for monitoring all support in the search for clots system, as well as observing the pressures of the oxygenator membrane, and with the medical and physiotherapy team to discuss the gasometric values and possible adjustment of the gas pressures and equipment flow. On the fifth day of ECMO, the patient remained stable. Due to this, with acceptable values of gasometry and radiographic findings, it was decided to withdraw the support temporarily to evaluate if the patient presented some decompensation.There was no diference in respiratory condition, the patient was decannulated with no complication and extubation was performed after 12 hours of turn off ECMO. Patient was discharged to intermediate unit after ninth day of ICU. Finally, there were 21 days of hospitalization, followed by hospital discharge with recovered lung function and good general clinical condition for the residence.

**Conclusions:** This case had a favourable outcome to the patient, the training of the multiprofessional team in ECMO is essential for good results. Nursing is inserted in this context by performing actions aimed at ensuring the safety of the procedure, identifying signs suggestive of possible complications and acting in decision making with the team.

**Reference**

1. Repessé X, Aubry A, Vieillard-Baron A. On the complexity of scoring acute respiratory distress syndrome: do not forget hemodynamics!. J Thorac Dis. 2016;8(8):E758-E764.

### P39

### Electrical impedance tomography at clinical practice

Karina T. Timenetsky, Denise Carnieli-Cazati, Carolina S.A. Azevedo, Marcelo Beraldo, Raquel A.C. Eid
HIAE - Critically Ill Department,Hospital Israelita Albert Einstein, São Paulo, SP, Brasil
*Critical Care* 2017, **21(Suppl 2):**P39

**Introduction:** Electrical impedance tomography (EIT) monitors bedside lung ventilation distribution, allowing lung heterogeneity diagnosis, as lung collapse, overdistension, and ventilation asynchrony (1,2).

**Objective:** To describe cases of EIT clinical applicability and its impact in therapeutic management of critically ill mechanically ventilated patients.

**Methods:** Observational, descriptive case series study of electrical impedance tomography use to guide bedside mechanical ventilation (MV) during a six month period.

**Results:** 7 patients had hypoxemic respiratory failure and necessity to adjust ventilator setting in order to reverse hypoxemia. Bedside PEEP titration: 4 patients were submitted to lung recruitment and PEEP titration guided by the EIT in order to choose a PEEP level with the least lung collapse. The median PaO2/FiO2 ratio before recruitment was 158 (100-170). After lung recruitment and PEEP titration PaO2/FiO2 ratio improved, with a median value of 264 (190-302). The PEEP level varied between these patients, as two patients after lung recruitment showed no lung collapse during PEEP titration, maintaining a PEEP level of 10 and 12 cmH2O. Before lung recruitment an empirical PEEP level of 10 and 18 respectively. The other two patients that had PaO2/FiO2 ratio lower than 200, during PEEP titration there was lung collapse, after adjusting the PEEP level by EIT there was an improvement in gas exchange (160 improving to 302, and another patient from 143 to 240). The initial PEEP levels of these patients were 10 and 12 cmH2O and after PEEP titration, 14 and 20 cmH2O. EIT to minimize lung overdistension: One patient had bonchopleural fistula after lung biopsy, with a PaO2/FiO2 ratio of 100. Initially, PEEP level was empirically adjusted to 18 cmH2O, with no improvement in gas exchange. At EIT baseline a flattening image in the upper right lung was observed representing a gain in the bronchopleural fistula debit. After choosing PEEP by EIT there was an improvement in the upper right lung ventilation. The second patient submitted to unilateral lung transplantation due to lung fibrosis had PaO2/FiO2 ratio of 225 with a PEEP level of 6 cmH2O and FiO2 of 60%. This low gas exchange was due to the non-transplanted lung, and to avoid lung overdistension during MV EIT was installed. The third case patient with Non Hodgkin Lymphoma had an air cyst and EIT was maintained to guide patient's MV in order to avoid barotrauma and lung injury.

**Conclusions:** Electrical impedance tomography seems a very helpful tool in clinical practice of patients in mechanical ventilation.

**References**

1. Victorino JA, Borges JB, Okamoto VN, Matos GF, Tucci MR, Caramez MP, et al. Imbalances in regional lung ventilation: a validation study on electrical impedance tomography. Am J Respir Crit Care Med 2004;169:791–800.
2. Costa EL V, Amato MBP. Electrical impedance tomography in critically ill patients. Clin Pulm Med 2013;20:178–86.

### P40

### Use of a new isokinetic device oriented by software for inspiratory muscle training in prolonged weaning

Bruno Leonardo Guimarães[1,3], Leonardo de souza[2,4], Fernando Guimaraes[4], Hebe Cordeiro[3], Fernanda Puga[3], Cristiane Almeida[3], Sergio Alvim[3], Thiago Regis[3], Jocemir Lugon[1]
[1]UFF - Universidade Federal Fluminense, Niterói, RJ, Brasil; [2]UNESA - Universidade Estácio de Sá, Niterói, RJ, Brasil; [3]HNMD - Hospital Naval Marcílio Dias, Rio de Janeiro, RJ, Brasil; [4]HI - Hospital Icaraí, Niterói, RJ, Brasil
*Critical Care* 2017, **21(Suppl 2):**P40

**Introduction:** Introduction: It is well established that inspiratory muscle training (IMT) improves muscle performance in hospitalized individuals [1]. Nowadays, patients on prolonged ventilation are among the major challenges of intensive care [2,3]. In this regard, specific exercises for respiratory muscles can result in better functional performance in critically ill patients during weaning process [1].

**Objective:** To evaluate the effects of inspiratory muscle training (IMT) program on patients in prolonged weaning.

**Methods:** Prospective randomized controlled trial. By the time participants were judged as apt for ventilator weaning, they underwent IMT with a new device called POWERbreathe K-5 (UK), Intervention group, or were managed in a conventional way with a tracheal collar, Control group. The primary endpoint was successful weaning. Muscle strength, and 30 days after intervention survival in the ICU were also analysed. Inspiratory strength was measured by the timed inspiratory effort index (TIE) employing a digital vacuometer MVD300 (Globalmed, Brazil) [4].

**Results:** Sixty-five tracheostomized patients were selected. Thirty-four patients comprised the Intervention group (20 male, mean age $65 \pm 17$ years, mean APACHE II score $28.9 \pm 4.8$). The weaning process duration was $13.7 \pm 8.8$ days, and the initial and final TIE indexes were $0.8 \pm 0.3$ and $1.7 \pm 0.9$, respectively ($P = 0.0001$). Thirty patients (88%) were successfully weaned, and 11 (31%) had a fatal course. The control group encompassed 31 patients (14 male, mean age $68 \pm 16$ years, mean APACHE II score $26.1 \pm 5.5$). The weaning process duration was $21.7 \pm 16.4$ days, and the initial and final TIE indexes were $0.8 \pm 0.6$ and $1.0 \pm 0.8$ respectively ($P = 0{,}053$). Thirteen cases (42%) were successfully weaned, and 22 (63%) had a fatal course. The 30 days after intervention survival rates for the intervention and control groups were 79% and 44%, respectively ($P = 0.025$), Fig. 30.

**Conclusions:** In support to our working hypothesis, the rate of weaning success and the survival rate 30 days after intervention were higher for patients undergoing inspiratory muscle training.

**Reference**

1. Mark E, Ruth D. Inspiratory muscle training facilitates weaning from mechanical ventilation among patients in the intensive care unit: a systematic review Journal of Physiotherapy 61 (2015) 125–134. 2. Boles JM, Bion J, Connors A, Herridge M, Marsh B, Melot C, et al. Weaning from mechanical ventilation. Eur Respir J. 2007; 29:1033-56. 3.Jubran A, Grant BJB, Duffner LA, Collins EG, Lanuza DM, Hoffman LA, Tobin MJ. Effect of Pressure

Support vs Unassisted Breathing through a Tracheostomy Collar on Weaning Duration in Patients Requiring Prolonged Mechanical Ventilation. A Randomized Trial. JAMA. 2013; 309: 671-677. 4.Souza LC, Guimarães FS, Lugon JR: Evaluation of a new index of mechanical ventilation weaning: the timed inspiratory effort. J Intensive Care Med; 2015a;30(1):37-43.

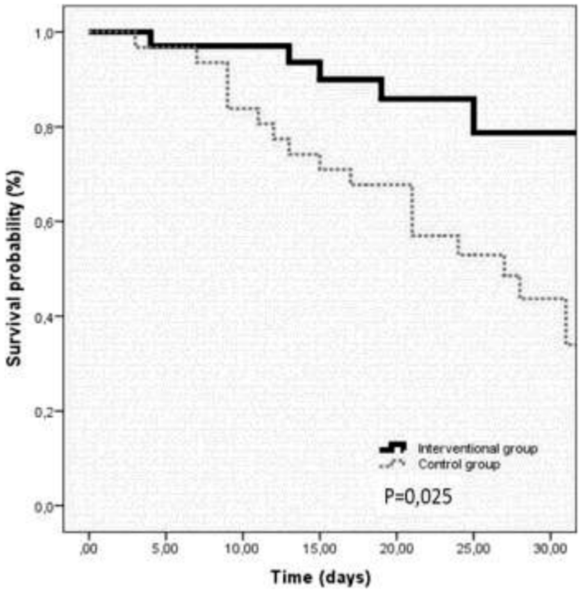

**Fig. 30 (Abstract P40).** Survival probability between interventional and control group

### P41

### Accuracy of the new timed inspiratory effort (TIE) index in prolonged weaning

Bruno Leonardo Guimarães[2,4], Leonardo Souza[1,3], Fernando Guimarães[2], Jocemir Lugon[2]
[1]UNESA - Universidade Estácio de Sá (Niterói, RJ, Brasil); [2] UFF - Universidade Federal Fluminense (Niterói, RJ, Brasil); [3]HI - Hospital Icaraí (Niterói, RJ, Brasil); [4]HNMD - Hospital Naval Marcílio Dias (Rio de Janeiro, RJ, Brasil)
*Critical Care* 2017, **21(Suppl 2):**P41

**Introduction:** Nowadays, patients on prolonged ventilation are among the major challenges of intensive care [1]. The recent timed inspiratory effort index (TIE) has been shown to be a clinically valuable tool to predict the success of ventilatory weaning, especially for patients with difficult weaning [2].
**Objective:** To validate the accuracy of the TIE index in patients in prolonged weaning.
**Methods:** This prospective observational study was approved by the hospital research ethics committee under the number CAAE: 50264415.2.000.5256. The exams were performed, evaluated and classified at the beginning and after weaning outcome. The area under the ROC curves was used to validate the accuracy of the TIE index, and the Youden method for calculating the cut-off point. The Med Calc version 12.1 program was used for statistical analysis.
**Results:** Sixty-five patients were selected, 33 men (51%), mean age $67 \pm 16$ years. All were tracheostomized, mean duration of mechanical ventilation was $22.7 \pm 14.1$ days, and APACHE II was $27.6 \pm 5.4$, 41 (63%) were successfully weaned, but 31 (47.7%) had a fatal course. One hundred and thirty examinations were performed during the prolonged weaning process. The sensitivity was 87.8 and the specificity was 88.8. The cut-off point > 0.99 was established to predict success at weaning. More relevant to the purpose of the study was the area under the ROC curve of $0.93 \pm 0.02$ (Fig. 31).
**Conclusions:** The performance of the TIE index as a predictor in prolonged weaning was consistent with previous publications reinforcing that it can be a precious tool in this setting.

### References

1. Jubran A, Grant BJB, Duffner LA, Collins EG, Lanuza DM, Hoffman LA, Tobin MJ. Effect of Pressure Support vs Unassisted Breathing through a Tracheostomy Collar on Weaning Duration in Patients Requiring Prolonged Mechanical Ventilation. A Randomized Trial. JAMA. 2013; 309: 671-677.
2. Souza LC, Guimarães FS, Lugon JR: Evaluation of a new index of mechanical ventilation weaning: the timed inspiratory effort. J Intensive Care Med; 2015a;30(1):37-43.

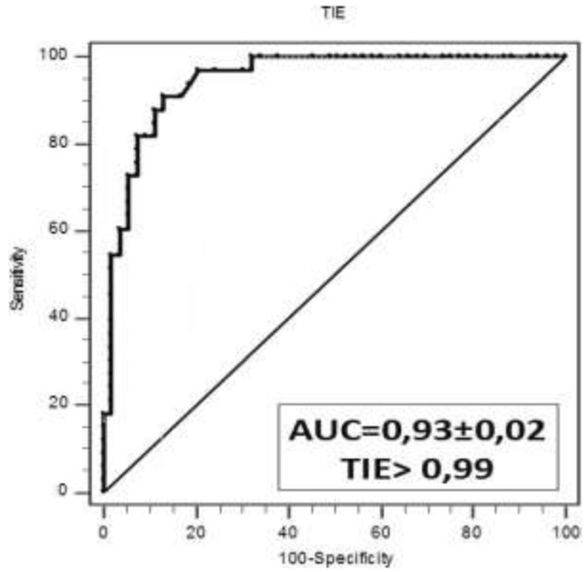

**Fig. 31 (Abstract P41).** ROC curve accuracy TIE index

### Safety/ Quality/ Management

### P42

### - Oncologists' and intensivists' attitudes towards the care of critically ill cancer patients

Antonio Paulo Nassar Jr[1,4], Aldo Lourenço Abadde Dettino[1], Cristina Prata Amendola[2], Rodrigo Alves dos Santos[2], Daniel Neves Forte[3,4], Pedro Caruso[1,4]
[1]ACCC - AC Camargo Cancer Center (São Paulo, SP, Brazil); [2]Pio XII - Pio XII Foundation, Barretos Cancer Hospital (Barretos, SP, Brazil); [3] HSL - Hospital Sirio Libanes (São Paulo, SP, Brazil); [4]HCFMUSP - Hospital das Clinicas, FMUSP (São Paulo, SP, Brazil)
*Critical Care* 2017, **21(Suppl 2):**P42

**Introduction:** Cancer patients represent an important proportion of intensive care unit admissions[1]. Oncologists and intensivists have distinct knowledge backgrounds, and conflicts about the appropriate management of these patients may emerge.
**Objective:** To compare the proportion of oncologists and intensivists who favored withdrawal of life support measures for two critically ill cancer patients.
**Methods:** We surveyed oncologists and intensivists at two academic cancer centers regarding their management of two hypothetical patients with different cancer types (metastatic pancreatic cancer and metastatic breast cancer with positive receptors for estrogen, progesterone and HER-2) who develop septic shock and multiple organ failure. We asked two questions in each case vignette: 1. What would be your choice regarding ICU admission? 2. Considering the patients was admitted to ICU and full support was instituted, but the patient's clinical condition continued to deteriorate after three days of full support, how would you then define the patient status? a) Full code b) Withholding of life support measures; c) Withdrawal of life support measures.

**Results:** Sixty intensivists and 46 oncologists responded to the survey. More intensivists than oncologists [45/60 (75%) vs. 24/46 (52%), p = 0.02] opted for ICU admission with restriction of life support measures for the pancreatic cancer patient (Fig. 32a). More intensivists than oncologists favored an ICU admission with restrictions of life support measures [46/59 (78%) vs. 27/46 (59%), p = 0.055] (Fig. 32b). Oncologists and intensivists similarly favored withdrawal of life support measures for the pancreatic cancer patient [33/46 (72%) vs. 48/60 (80%), p = 0.45] (Fig. 32c). On the other hand, intensivists favored more withdrawal of life support measures for the breast cancer patient than oncologists [32/59 (54%) vs. 9/44 (21%), p < 0.001]. In the multinomial logistic regression, the oncology specialists were more likely to advocate for a full code status for the breast cancer patient (OR = 5.931; CI 95%, 1.762-19.956; p = 0.004). On the other hand, no variable was associated with withdrawal of life support measures for the pancreatic cancer patient.

**Conclusions:** Oncologists and intensivists share different views regarding life support measures in critically ill cancer patients. Oncologists tend to focus on the cancer characteristics, whereas intensivists focus on multiple organ failure when weighing in on the same decisions. Regular meetings between oncologists and intensivists may reduce possible conflicts regarding the critical care of cancer patients.

### Reference

1. Soares M, Caruso P, Silva E, Teles JM, Lobo SM, Friedman G, Dal Pizzol F, Mello PV, Bozza FA, Silva UV et al: Characteristics and outcomes of patients with cancer requiring admission to intensive care units: a prospective multicenter study. Crit Care Med 2010, 38(1):9-15.

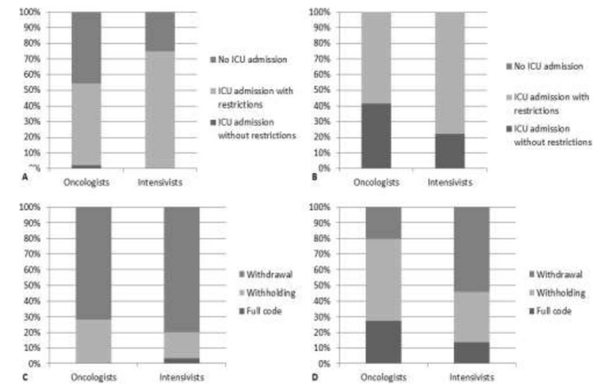

**Fig. 32 (Abstract P42).** Answers to the survey. (**a**) ICU admission of the pancreatic cancer patient, (**b**) ICU admission of the breast cancer patient, (**c**) Code status of the pancreatic cancer patient after three days of ICU full-support, and (**d**) Code status of the breast cancer patient after three days of ICU full-support

### P43

### Impact of urgent chemotherapy in critically ill patients: a propensity score matched retrospective study

Maria Cristina Oliveira[1], Antonio Paulo Junior[1], Juliana Ferreira[1,2], Aldo Dettino[1], Pedro Caruso[1,2]

[1]HACC - Hospital A.C. Camargo (São Paulo, SP, Brasil); [2]InCor - HCFMUSP - InCor - Hospital das Clínicas da FMUSP (São Paulo, SP, Brasil)

*Critical Care* 2017, **21(Suppl 2):**P43

**Introduction:** Some critically ill patients receive urgent chemotherapy that is nowadays considered feasible [1-3]. Urgent chemotherapy has the potential to decrease or increase the mortality in critically ill patients, but its effect on mortality was only evaluated in descriptive studies and not in studies comparing similar patients that received or not urgent chemotherapy.

**Objective:** The primary objective of the present study was to compare the ICU mortality, hospital mortality and the long-term survival between critically ill patients that received or not urgent chemotherapy during the ICU stay. The secondary objective was to compare the effect of urgent chemotherapy on mortality and survival of patients with hematological malignancies or solid tumors.

**Methods:** We designed a retrospective observational study including adult patients that received at least one day of urgent intravenous chemotherapy for a cancer-related life-threatening complication. Using the propensity score method and adjusting for ten different variables, we elected a population of critically ill patients that not received urgent chemotherapy but was similar to the one that received. We compared the intensive care unit mortality, hospital mortality and long-term survival between patients that received nor not urgent chemotherapy. As a subgroup analysis, we compared the mortality of patients with solid tumors or hematological malignancies.

**Results:** We included 47 patients that received urgent chemotherapy and 94 matched patients that did not receive it. At intensive care unit admission, the groups were similar except that patients that received urgent chemotherapy less frequently had received chemotherapy before the intensive care unit. The intensive care unit (48.9% vs 23.4%; P < 0.01) and hospital (76.6% vs 46.8%; P < 0.01) mortality of the patients that received urgent chemotherapy was higher than the patients that did not. Likewise, the long-term survival of patients that received urgent chemotherapy was lower (P < 0.01). The subgroup analysis showed that the higher mortality was limited to the patients with solid tumors, while the urgent chemotherapy did not change the mortality of the patients with hematological malignancies (Fig. 33).

**Conclusions:** The use of urgent chemotherapy in unselected critically ill patients increases the ICU mortality, hospital mortality and decrease the long-term mortality in patients with solid tumors but not in patients with hematological malignancies.

### References

1. Azoulay E. A new standard of care for critically ill patients with cancer. Chest 2014; 146: 241-244.
2. Benoit DD, Depuydt PO, Vandewoude KH et al. Outcome in severely ill patients with hematological malignancies who received intravenous chemotherapy in the intensive care unit. Intensive Care Med 2006; 32: 93-99.
3. Darmon M, Thiery G, Ciroldi M et al. Intensive care in patients with newly diagnosed malignancies and a need for cancer chemotherapy. Crit Care Med 2005; 33: 2488-2493.

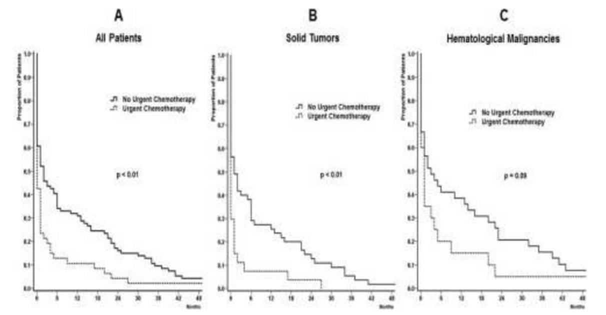

**Fig. 33 (Abstract P43).** Kaplan-Meier survival curves for patients that received urgent chemotherapy

### P44

### Correlation between the time of nursing care and care quality indicators in Intensive Care Units

Paulo Garcia[1], Daisy Tronchin[2], Fernanda Fugulin[2]
[1]HU USP - Hospital Universitário da Universidade de São Paulo (São Paulo, SP, Brasil); [2]EEUSP - Escola de Enfermagem da Universidade de São Paulo (São Paulo, SP, Brasil)
*Critical Care* 2017, **21(Suppl 2):**P44

**Introduction:** Introduction: The evidence of association between nursing care time spent with patients and healthcare quality indicators may be an important management tool, supporting the decision-making process on issues related to quantitative and qualitative adequacy of nursing professionals when handling the care quality and safety standards needed by health services.
**Objective:** Objective: To investigate the correlation between average times of nursing care spent with adult patients in intensive care units (ICU) in São Paulo and care quality indicators: Non Planned Incidence of Loss of Oro / Nasogastric tube (NGT) for Nutritional Intake; Non Planned Extubating (NPE) incidence of Endotracheal Cannula; Incidence of Loss of Central Venous Catheter (CVC).
**Methods:** Method: this is a quantitative observational, correlational study carried out in 11 adult ICU patients in two public and one private hospital, located in the city of São Paulo. The population understood quantity and quality records of nursing professionals, the number of patients with at least one of therapeutic devices (NGT, NPE, CVC) and occurrences relating to device losses. Hospitals were nominated by HA, HB and HC initials. Data collection in HA and HB, occurred in the period from 17 July to 17 November 2015. In HC data were collected from August 12 to December 12, 2015. The analysis was based on descriptive and inferential statistics, with 5% significance.
**Results:** Results: 2,569 patients were assisted in the four months of the study. The average time of nursing care spent with patients accounted for 18, 86 hours at HA, HB and 21 hours at HB and 19, 50 at the HC. The average percentage of time dedicated by the nurses was 37.75% in HA, 35.00% in HB and 41.36% in HC. The indicator Non Planned Loss of NGT for Nutritional Intake averaged 2.19 / 100 patient-days (SD = 10.93). The average indicator incidence of NPE of Endotracheal Cannula corresponded to 0.42 / 100 patient-days (SD = 4.51) and the average indicator Incidence loss of CVC was 0.22 / 100 patient-days (SD = 2, 04). There was no statistically significant correlation between the average time of nursing care spent by the team, average time spent by professional category and the mentioned quality indicators.
**Conclusions:** Conclusions: Though the study hypothesis was disproved, this research moves towards elucidating other variables that may affect the correlation between adverse events related to the analyzed therapeutic artifacts and the nursing professionals. The results of this study may support methodological decisions for the verticalization of the technical / scientific knowledge in nursing and the management of future researches aiming to demonstrate the impact of nursing human resources in the quality and safety of patients, health professionals and institutions.

### P45

### Analysis of adverse events during intra-hospital transportation of critically ill patients

Francielli Gimenez[1], Tomás Moreira[1], Jeisibel Maroco[1], Isadora Grion[2], Josiane Festti[1], Claudia Carrilho[1], Cintia Grion[1]
[1]UEL - Universidade Estadual de Londrina (Londrina/Paraná/Brasil);
[2]UNICESUMAR - Centro Universitário de Maringá (Maringá/Paraná/Brasil)
*Critical Care* 2017, **21(Suppl 2):**P45

**Introduction:** Intra-hospital transport is defined as the temporary or definitive referral of patients within the hospital environment and may have a diagnostic and/or therapeutic purpose. It is a complex activity and must ensure the preservation of clinical conditions to those who are transported, throughout the course of the procedure.
**Objective:** To describe the adverse events occurring during the intra-hospital transportation of adult patients hospitalized in an Intensive Care Unit (ICU) and to evaluate the association with morbidity and mortality.
**Methods:** A prospective cohort study conducted from July 2014 to July 2015. Data collection comprised demographic data and clinical data, such as medical diagnosis, prognostic scores of the Sequential Organ Failure Assessment (SOFA) and Simplified Acute Physiology Score (SAPS 3), the presence of comorbidities, length of stay, and outcome at discharge from the ICU and hospital. Data was also collected on transport and adverse events. Adverse events were classified according to the World Health Organization following the degree of damage into: None, Mild, Moderate, Severe, and Death. The level of significance was set at 5%.
**Results:** A total of 293 patients were analyzed during the study period, with follow-up of 143 patient transportations and records of 86 adverse events. Of these events, 44.1% were related to physiological alterations, 23.5% occurred due to equipment failure, 19.7% due to team failure, and 12.7% due to delays. Half of the events were classified as moderate degree. The mean time spent in the ICU of the group that presented adverse events during transportation was higher when compared to patients transported without the occurrence of adverse events (21.7 versus 9.2 days respectively, $p < 0.001$), as was the mean time of hospital stay (31.4 versus 16.6 days, respectively, $p < 0.001$). No difference in mortality was found between these two groups of patients.
**Conclusions:** Adverse events were frequent during intra-hospital transportation of critically ill patients. Physiological alterations were the most frequently encountered events, followed by equipment and team failures. The degree of damage associated with the adverse events was classified as moderate in most cases and associated with an increase in the length of ICU and hospital stay.

**References**

Blakeman TC, Branson RD. Inter- and intra-hospital transport of the critically ill. RespirCare. 2013 Jun;58(6):1008-23.
Jia L, Wang H, Gao Y, Liu H, Yu K.High incidence of adverse events during intra-hospital transport of critically ill patients and new related risk factors: a prospective, multicenter study in China. Crit Care. 2016 Jan 18;20:12.

### P46

### Customer satisfaction: reducing complaints in the SAC in real time

Maira Lima, Adriana Marcos, Debora Schettini, Neide Lucinio
HIAE - Department of Severe Patients Morumbi (São Paulo, SP, Brasil)
*Critical Care* 2017, **21(Suppl 2):**P46

**Introduction:** Customer satisfaction is an essential issue for companies to stay on the market. In the hospital environment, the dissatisfied customer spreads negative information and thus the image of the organization is impaired, therefore, customer satisfaction is an important marketing tool that can be used to retain customer loyalty, avoid complaints and make the company more competitive in the Marketplace.
**Objective:** The objective of this study is to evaluate in real time the satisfaction of the client regarding the assistance offered during the hospitalization in the Intensive and Semi-Intensive Therapy and consequently reduce the number of complaints in the SAC.
**Methods:** Used the Ishikawa diagram, brainstorming and 5W2H tools. The Care Team defines as action strategy for the year, chooses customer satisfaction as a value proposition, bother with high number of complaints, performs mapping before implementation of the customer satisfaction index project: Client / family / medical team, performs first draft of the project and presents for nursing leadership, defines vision of the unit - "to be a reference in patient satisfaction, Coordination of the area meets with team - strengthens the project, Establishment of the first channel Communication with client - Visit the seniors in the first 48 hours of hospitalization (routines / glossary items / team presentation), Defines metrics: no complaints X no exits, Elaborates and implants satisfaction flag - "Lighthouse of satisfaction", Elaborates an active search Complaint: "Near complaint", Identifies patients at risk for complaint and visits the bedside (active unstructured search), Project structure - triggering Stakeholders

**Results:** There were 97 patient visits to the ICU during the data collection period, with 64% visits by senior nurses to guide the filling of the satisfaction lighthouse, of these 52% were in the first 48 hours of hospitalization, 8% reported dissatisfaction. 2% related to the courtesy of the multiprofessional team, 2% hygiene of the apartment, 3% furniture, 1% nursing practice. All non-formalized complaints were resolved in a timely manner, even during ICU patient admission, thereby avoiding 8 formal complaints to the critical care sector.

**Conclusions:** Future actions will be necessary to disseminate and expand this method of work for every institution. As well as creating a dynamic framework to assist the quantification of data and to spread new collaborators with the Patient Satisfaction Week

**References**

Aasland, O. G., Olff, M., Falkum, E., Schweder. T. & Ursin, H. (1997). Health complaints and job stress in norwegian physicians: the use of an overlapping questionnaire design. Social Science and Medicine, 45 (11), 1615-1629.

Begley, T. M. & Czajka, J. M. (1993). Panel analysis of the moderating effects of commitment on job satisfaction, intent to quit, and health following organizational change. Journal of Applied Psychology, 78 (4), 552-556.

### P47

### Implementation of a managed sepsis protocol based on the IHI-quality improvement model in a city hospital in São Paulo, Brazil: report of the experience

Leidy Katerine Calvo Nates[1,2], Ariano Jose Pereira[1,2], Antonio Capone Neto[1,2], Eliezer Silva[1,2]
[1]HMVSC - Hospital Municipal da Vila Santa Catarina (São Paulo,SP,Brasil);
[2]HIAE - Hospital Israelita Abert Einstein (São Paulo,SP,Brasil)
*Critical Care* 2017, **21(Suppl 2):**P47

**Introduction:** Although 13 years have passed since the first publication of the international treatment guidelines, sepsis continues to be the leading cause of death in Brazilian Intensive Care Units (ICU), with lethality rates of 50% [1].

**Objective:** The present study reports our experience at implementing a Managed Sepsis Protocol (MSP) in a public hospital. This action was based on the Quality Improvement Model (QIM), a methodology proposed by the Institute for Healthcare Improvement (IHI –USA).

**Methods:** The study is a longitudinal, prospective, non-randomized one, lasting 18 months (Jan-16 to Jun-17), and took place in a high complexity municipal hospital of the city of São Paulo. Applying the IHI methodology required establishing Multidisciplinary Work Teams (MWT), in 3 phases (ICU, ward and emergency department - ED). Area-specific driver diagrams were developed as well as "Plan-Do-Study-Act"-PDSA cycles in order to test the proposed changes and improvements. Semi-structured interviews were performed with members of MWT and hospital staff.

**Results:** The main challenges identified by the research team were: need of adaptation the new local MSP, poor clinical staff engagement, problems with case notifications and data collection, lack of alarm systems to identify, absence of a reference multidisciplinary team, inadequate settings of priorities, unclear patient flows and team member roles. The interviewed MWT members (8 in total) agreed to the need of improving the procedures regarding the care for septic patients and that the use of the QIM in MSP implementation improved their work performance by making it more synchronized, faster, on time and more standardized. Furthermore, they did not perceive workload increases and reported satisfaction with the used methodology, as they became participants in the decision making processes. In addition, specific management changes were introduced and sustainable long lasting improvements are expected to improve recognition, diagnosis and treatment of sepsis, and reduce lethality.

**Conclusions:** The IHI quality-improvement model seems to be a suitable tool to implement sepsis management protocols and should be further tested. Full results of this study will be available by the end of 2017, as quantitative analysis about its impacts on outcomes (lethality rates), processes (compliance 3-hour care bundles) and staff opinions about workload.

**Reference**

1. Instituto Latino-Americano para Estudos da Sepse. Sepse: Um problema de saúde pública. Brasília (DF): Conselho Federal de Medicina (BRA); 2015. Disponível em: http://www.ilas.org.br/materiais-adulto.php.

### P48

### Patients with oncological terminal stages and its relations: student medical school perspective

Enzo Santiago Rezende[1], Ana Lídia Ferreira de Oliveira[1], Luiz Henrique de Almeida Ventura[1]
[1]FASA - Faculdades Santo Agostinho (Vitória da Conquista, BA, Brasil);
[2]FASA - Faculdades Santo Agostinho (Vitória da Conquista, BA, Brasil);
[3]FASA - Faculdades Santo Agostinho (Vitória da Conquista, BA, Brasil)
*Critical Care* 2017, **21(Suppl 2):**P48

**Introduction:** Cancer is the third major cause of death in Brazil. The students of medical schools should know how to deal with patients diagnosed with oncological final stages very carefully. The care of the patient in these oncological final stages must be holistic and consider his biosocial environment. Nowadays, procedements and therapeutic diagnosis try to improve the patient´s quality of life. It also gives to the patient palliative cares. In this bibliographic review, we found articles with strong evidences about the professional’s difficulties on how to deal with oncological final stages patients. Those healthcare professionals try to preserve life and see the death of a patient as a loss.

**Objective:** This study intends to verify how the formation of the new medical professionals is in front of the terminal oncologic phase; Analyze how the contact with the patient in this period affects the professional future; As well as discussing the importance of the multiprofessional team against these patients.

**Methods:** For the construction of the article, the bibliographic review was also used, where references were made available in the Scielo and PubMed database. We selected articles published between 2006 and 2016, the research being carried out through four basic descriptors, the terminal patient, Cancer institutes, Humanization of care and Oncology. Thus articles were used as inclusion criteria in Portuguese, and exclusion of re-readings of works and articles with an exclusive view of nursing.

**Results:** Humanized care has increasingly been pointed out as the ideal way of taking care of health, considering an integrated vision and an adoption of an ethics of care, leading to a quality of care, but many patients are still not followed up in this way. It is indisputable that the institutions that involve the medical areas need to focus on this pillar of the training of future professionals always showing the importance of this concept in the improvement of the patient, since these professionals are finding difficulties in dealing with this type of situation.

**Conclusions:** It is important that the health professional understands the death process since graduation, in order to prepare it that can reduce stress and anxiety. In this way, he will be able to maintain an interpersonal relationship of help, which is the essence of the act of caring humanized.

**References**

1. Govidan, Ramaswamy; Arquette, Matthew. Washington Manual de Oncologia, Rio de Janeiro – RJ: Guanabara Koogan, 2002, páginas 203-236
2. FARES, Abdo. Et al. Tratado de Oncologia, Volume 02, São Paulo – Sp; Atheneu, 1°edição, 2013, páginas 1625-1647, 1731-1780
3. Figueiredo, Euridice; Monteiro, Mauro, Ferreira, Alexandre. Tratado de Oncologia, volume 1, Rio de Janeiro – RJ; Revinter 2013, páginas 795-859

### P49

### Analysis of the knowledge of adequate hygiene and profile of ultrasound users in an intensive care environment

Ricardo Minuzzi, Juliana Fernandes
Hed - Hospital Ernesto Dorneles (Porto Alegre, Rgs, Brasil)
*Critical Care* 2017, **21(Suppl 2):**P49

**Introduction:** Ultrasonography (US) has been increasingly used in intensive care units due to its easy access, low cost and also ability to guide the diagnosis of many clinical conditions, assist in procedures and punctures and also evaluate the response to specific therapies. However, ultrasound transducers and associated materials have been an increasingly important source of cross-infection and nosocomial infections.
**Objective:** To evaluate the knowledge about adequate hygiene and the profile of the users of ultrasound devices and probes in the emergency and intensive care units (ICU) of the Ernesto Dorneles Hospital during the 12-month period.
**Methods:** A prospective, cross-sectional study at a single medical center (Ernesto Dornelles Hospital), with a questionnaire analyzing the profile and knowledge about the ultrasonography of the physicians working in the emergency and ICU sections of the hospital. We interviewed with structured questionnaire all physicians contracted active of the respective sectors in the period of February 3, 2016 until February 3, 2017. For analysis were described categorical variables by frequencies and percentages
**Results:** The responses of 52 physicians (n = 52) were analyzed. It was seen that 31 (60%) of the participants were female. The predominant age group of the studied population was 20 to 40 years old (88%). 49 (95%) had already performed some type of specific training with the ultrasound device. The main reason for using the device was to guide procedures - 51 (98%). Finally, 43 (83%) participants concluded that they had never received any guidance and / or training on proper hygiene of the ultrasound device
**Conclusions:** Most physicians, with a labor relationship with the institution, who use ultrasound in their work routine, have never received guidelines and or formal instructions on proper hygiene techniques of the device. The numbers reached by this study are in agreement with the results found in research carried out in other institutions, which reinforces the need for more investments and studies in this area and the creation of a protocol to be followed by the institution.

**Reference**

1. Miyague André Hadyme, Mauad Fernando Marum, Martins Wellington de Paula, Benedetti Augusto César Garcia, Ferreira Ana Elizabeth Gomes de Melo Tavares, Mauad-Filho Francisco. O exame ultrassonográfico como potencial fonte de infecção cruzada e nosocomial: uma revisão de literatura. Radiol Bras, 2015 Oct [cited 2016 Sep 01] ; 48( 5 ): 319-323. 2.Flato Uri Adrian Prync, Petisco Gustavo Mascari, Santos Fernanda Bezerra dos. Punção venosa guiada por ultra-som em unidade de terapia intensiva. Rev. bras. ter. Intensiva [Internet]. 2009 June [cited 2016 Sep 01] ; 21( 2 ): 190-196. 3.Nasri Carolina O, Martins Wellington P. Higienização dos transdutores ultrassonográficos. Experts in Ultrasound: Reviews and Perspectives. 2011; 3(2): 38-40. 4.Ridley EL. Ultrasound probe cleaning education may be lacking. Disponível em: http://www.auntminnie.com/index.aspx?-sec=ser & sub = def & pag = dis & ItemID = 85322.

### P50

### Successful strategy to reduce ventilator-associated pneumonia hospital municipal in Sao Paulo Brasil

Ana Helena Andrade, Roseleine Rosa, Jane Catarina Ananias, Victor Leonardo Amorim, Vinicius Agibert De Souza, Carlos Alberto Abreu
HMBOIMIRIM - Hospital Municipal Dr Moysés Deutsch Municipal Hospital (Sao Paulo)
*Critical Care* 2017, **21(Suppl 2):**P50

**Introduction:** Ventilator-associated pneumonia (VAP) is a major complication of patients admitted to intensive care and follow-up is necessary to implement preventive actions, as well as analyze the results and train as teams. VAP rates in Brazil are higher than those related in Europe and USA.
**Objective:** was to examine the effect of the Institute for Healthcare Improvement's ventilator bundle plus oral decontamination with chlorhexidine (ODC) in the incidence of VAP in an intensive care unit.
**Methods:** The study was conducted in a 20-bed, medical-surgical ICU. Criteria for nosocomial pneumonia are those from the CDC. Strategy was to implement the IHI's ventilator bundle plus ODC. The goals were the ICU team adhesion of 80% achieved in ninth month after bundle implementation and 100% after one year of follow up. These measures included five strategies to prevent ventilator-associated pneumonia: 30°- 45° elevation of the head of the bed, adequate sedation level (RASS 1 or 2), DVT/PE prevention, peptic ulcer prophylaxis and oral decontamination with chlorhexidine 0.12%. From February 2012 on, the ICU nursing staff and ICT performed a daily checklist in order to observe the five issues accomplishment. If any item was found to be inadequate it was promptly corrected.
**Results:** February 2012 and December 2016, adherence to the whole package was gradually increased up to 100%, respectively ($p < 0.001$). VAP density was proportionately smaller to group membership in the same period, 20 per 1000 ventilation / day and 4.5, respectively. In 2015 - 2016 zero density VAP.
**Conclusions:** Initial VAP rates were extremely high even for Brazilian benchmarks. Although we could not implement expensive technologies like continuous aspiration of subglottic secretions, ICU team and ICT efforts were crucial for satisfactory results, as well the administrative board support, which turned this issue an institutional priority. Our goals are to reduce even more, implementing "ventilator bundle—getting to zero" program, maintaining a continuum effort to sustain these results.

**Reference**

American Thoracic Society. Guidelines for the management of adults with hospital-acquired, ventilator-associated, and healthcare-associa- ted pneumonia. Am J Respir Crit Care Med, Diretrizes sobre Pneumonia associada a Ventilação mecânica (PAV)

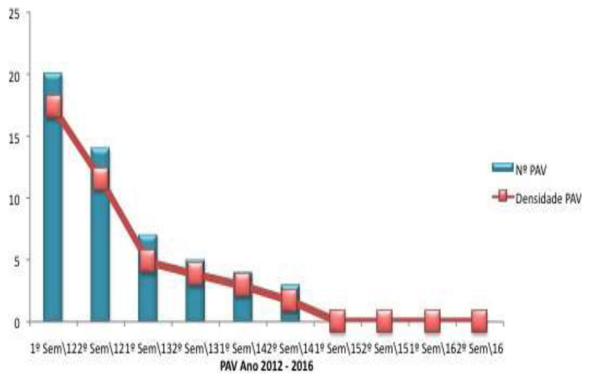

**Fig. 34 (Abstract P50).** Distribution semiannual number PAV density Year 2012 - 2016

### P51

### Impact mobilize early in critical patients admitted in ICU - Municipal Hospital Sao Paulo Brasil

Ana Helena Andrade, Ulisses Pereira, Ana Paula Paiva, Jane Catarina Ananias
HMBOIMIRIM - Dr Moysés Deutsch Municipal Hospital (Sao Paulo, Sao Paulo, Brasil)
*Critical Care* 2017, **21(Suppl 2):**P51

This abstract is not included here as it has already been published [1]

[1] Intensive Care Medicine Experimental 2016, 4(Suppl 1):28

### P52

### An unstructured quality improvement program to reduce opioid consumption in mechanically ventilated patients is effective and may reduce duration of mechanical ventilation: a retrospective before-and-after cohort study

Bruno Adler Maccagnan Pinheiro Besen, Carlos Eduardo Brandão, Carla Marchini Dias Silva, Lucas Fernandes de Oliveira
HLuz - Hospital da Luz - Vila Mariana - Rede NEXT (São Paulo, SP, Brasil)
*Critical Care* 2017, **21(Suppl 2):**P52

**Introduction:** Fentanyl is a commonly prescribed opioid for the treatment of pain in patients undergoing mechanical ventilation. In Brazil, a 50 mcg/mL solution is commonly used in clinical practice, although doses of 10 mcg/h may be enough to achieve pain resolution.
**Objective:** We aimed to compare fentanyl consumption between a "before" period of concentrated solution and an "after" period of diluted solution use and pain assessment and treatment training.
**Methods:** This is a retrospective cohort study with a historical control including all mechanically ventilated patients. A quality intervention program was implemented on October, 2014, in which a diluted solution of fentanyl (10 mcg/mL) was defined as the standard of care of the unit, which started on October, 2014, with the infusion starting at 1-2 mL/h and titrated according to patients' pain (either through a numerical rating scale or behavioral pain scale as assessed by nurses or physicians). We compared fentanyl consumption (ampules per month) between the first period (January, 2014 through September, 2014) and the second period (November, 2014 through December, 2015).
**Results:** There were 306 patients (Period 1, Before) and 515 patients (Period 2, After) in each period. Baseline characteristics were comparable between groups (Fig. 35). Fentanyl consumption (ampules/month) substantially reduced from 843.33 in Period 1 to 339.07 in Period 2 (Fig. 36). Median mechanical ventilation duration reduced from 2 days to 1 day ($p = 0.0001$) and median intensive care unit length-of-stay reduced from 5 days to 4 days ($p = 0.0371$). ICU and hospital mortality were unchanged between groups.
**Conclusions:** The use of a dilute fentanyl infusion and basic nurse and physician training resulted in lower opioid consumption, a shorter mechanical ventilation duration and shorter ICU length-of-stay. These results should be interpreted with caution, given that this is not a time-series analysis and we did not control for confounding factors.

| | Before Intervention (n = 306) | After Intervention (n = 515) | p-value |
|---|---|---|---|
| Age, years-old | 58.1 (19.0) | 59,3 (19.2) | 0.3753 |
| Male, n (%) | 138 (41.44%) | | 0.3051 |
| Type of Admission, n (%) | | | 0.436 |
| Medical | 254 (76.28%) | 350 (72.61%) | |
| Emergency Surgery | 27 (8.11%) | 50 (10.37%) | |
| Elective Surgery | 52 (15.62%) | 82 (17.01%) | |
| SAPS 3 score | 62.4 (19.4) | 60.2 (18.0) | 0.1506 |
| SOFA score | 4 [2, 7] | 4 [2, 6] | 0.1464 |
| Charlson Comorbidity Index | 2 [1, 4] | 2 [1, 4] | 0.5804 |
| Fentanyl Consumption (ampules / month) | 843.33 | 339.07 | |
| MV Duration, days | 2 [1, 7] | 1 [0, 4] | 0.0001 |
| ICU LOS, days | 5 [2, 10] | 4 [2, 8] | 0.0371 |
| Hospital LOS, days | 15 [7, 28] | 12 [6, 23] | 0.0009 |
| ICU Mortality, n (%) | 136 (40.84%) | 187 (38.80%) | 0.558 |
| Hospital Mortality, n (%) | 183 (54.95%) | 236 (48.96%) | 0.092 |

*Legend: MV, mechanical ventilation; ICU, Intensive Care Unit; LOS, Length-of-stay; SOFA, Sequential Organ Failure Assessment; SAPS, Simplified Acute Physiology Score.*

**Fig. 35 (Abstract P52).** Patient's characteristics and outcomes before and after the intervention

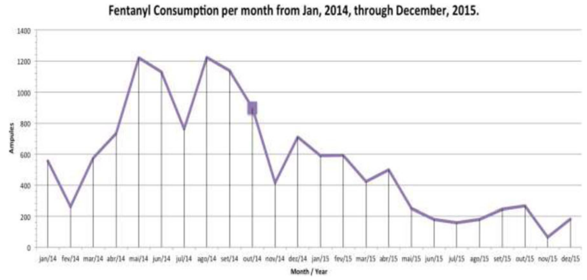

**Fig. 36 (Abstract P52).** Fentanyl consumption per month, from January, 2014, through December, 2015

### P53

### An extended and flexible visit protocol for ICU patients reduces delirium occurrence

Miriam Cristine Machado, Mayara Schirmer Moerschberger, Geonice Sperotto, Glauco Adrieno Westphal
Centro Hospitalar Un - Centro Hospitalar Unimed (Joinville (SC), Brazil)
*Critical Care* 2017, **21(Suppl 2):**P53

**Introduction:** The flexibilization of ICU visits possibly brings benefits to patients and their families, allowing a greater interaction between them. This interaction could reduce the incidence of delirium.
**Objective:** We evaluate the impact of the implementation of an extended and flexible visit protocol for ICU patients on delirium occurrence.
**Methods:** This is a before-after study conducted in a south-Brazilian hospital during 2015 and 2016. In 2015 (Phase I), visiting periods of up to 6 hours per day were offered to the relatives of patients hospitalized in the ICU. In 2016 (Phase II), we adopted an extended and flexible regime of visitation allowing the presence of family members for up to 24 hours/day. Critically ill patients were daily evaluated with the Intensive Care Delirium Screening Checklist (ICDSC). We compared the 2 cohorts in terms of the presence of delirium predisposition and delirium diagnosis.

**Results:** From the 676 ICU admissions, 482 patients were eligible for the ICDSC application (227 during Phase I and 255 during Phase II). The total number of delirium episodes decreased from 30.8% to 15.3% ($p < 0.001$) and patients with delirium remained stable (14.9% to 10.2%, $p = 0.11$) There were no differences between phases I and II in terms of patients with predisposition to delirium (n = 45, 19.8% to n = 59, 23.1%; $p = 0.37$). Among predisposed patients, there was a decrease in the occurrence of delirium (n = 23, 51.1%; to n = 10, 16.9%; $p < 0.001$). Regarding the number of occurrences, there was a tendency to increase the number of predisposition (n = 96, 42.2% to n = 128, 50.2%; $p = 0.08$), and a decrease in the number of delirium events (n = 59, 61.4% to n = 23, 17.9%; $p < 0.001$).

**Conclusions:** Our extended and flexible visit protocol seems to have contributed to reduce the rates of occurrence of delirium in ICU patients with predisposition to delirium.

### Sepsis/ Septic Shock

### P54

### Drug plasma measurements and PK/PD approach to guarantee Imipenem effectiveness against nosocomial pathogens in septic burn patients based on empiric dose regimen

Léonard da Vinci Kanda Kupa[1], João Manoel da Silva[2], Edvaldo V. Campos[2], Carlindo Vieira da Silva Jr[1], Silvia R. C. J. Santos[1], David S. Gomez[2]
[1]FCF USP - Pharmacokinetics Center, School of Pharmaceutical Sciences (Sao Paulo/SP/Brazil); [2] HCFMUSP - Division of Plastic Surgery and Burns (Sao Paulo/SP/Brazil); [3]FCF USP - 1Clinical Pharmacokinetics Center, School of Pharmaceutical (Sao Paulo/SP/Brazil); [4] HCFMUSP - Division of Anaesthesiology (Sao Paulo/SP/Brazil)
*Critical Care* 2017, **21(Suppl 2):**P54

**Introduction:** Usual antimicrobial dose regimen often cannot achieve drug effectiveness in critically ill septic burn patients (ICU) and may result in drug plasma levels below those required to reach effectiveness against the most common nosocomial pathogens, which might affect desired outcome1.

**Objective:** Based on pharmacokinetics-pharmacodynamic (PK/PD) approach, it was investigated imipenem effectiveness in burn patients receiving the initial dose regimen recommended against nosocomial susceptible strains.

**Methods:** Ethical approval was obtained from the Ethics Committee of hospital under approval number n°069/09-2015. Thirty four burn patients (ICU) undergoing treatment for septic shock were investigated. Recommended imipenem daily dose was 2 g eq. to 29 (27-31) mg/kg at dose regimen (0.5 g qh6 0.5 hr infusion) eq. 7.1 (6.7-7.7) mg/kg time inter doses (tid), and a series of four blood samples were collected (1.5 mL/each) for drug plasma measurement by liquid chromatography. Pharmacokinetics (PK) was performed by a noncompartmental data analysis. PK/PD approach was performed to estimate the probability of target attainment (PTA) based on the predictive index of drug effectiveness recommended to carbapenem agents (40%fT > MIC)2.

**Results:** Thirty four burn patients of both gender (11 F/23 M), 35 (31-40) yrs, 68 (65-71) kg, total burn surface area 39 (32-46) %, and Clcr 144 (129-159) mL/min, means (CI95%). Thermal/electrical injuries occurred in 29/5; inhalation injury, orotracheal intubation/vasoactive drugs occurred in 32/34 of them. It was obtained free drug in plasma trough level 1.8 (1.4-3.5) mg/L after the empiric daily dose 29 (27-31) mg/kg administered every 6 hrs at dose regimen 7.1 (6.7-7.7) mg/kg, means (CI95%), Fig. 37. Target was attained for all patients against MIC 2 mg/L strains isolated (Haemophylus influenza; Serratia marcencens) after the empirical dose regimen; while dose adjustment was required in 5/34 patients against MIC 4 mg/L, strains as Enterococcus spp, Morganella spp, Proteus spp, Pseudomonas spp, (Fig. 37). Imipenem PK showed to be altered by comparison of data obtained in septic burn patients with previously reported data in healthy adult volunteers3. Biological half-life was prolonged and the volume of distribution was increased by trice compared to adult healthy volunteers; a strong correlation between these parameters was obtained, (Fig. 38). Eradication of nosocomial pathogens occurred in 29/34 by the initial dose regimen, and daily dose was adjusted against MIC 4 mg/L strains in five of them for target attainment.

**Conclusions:** Imipenem PK is altered in burns with consequences in drug effectiveness. Desired outcome in general is reached based on TDM emphasizing the recommendation of PK/PD approach once a week during the antimicrobial therapy.

#### Reference

1. Varghese JM et al: Crit Care Clin 2011; 27:19-34 2. Ikawa K et al: J Infect Chemother 2008; 14: 330-332 3. Drusano GL et al: Antimicrob Ag Chemother 1984; 26: 715-721

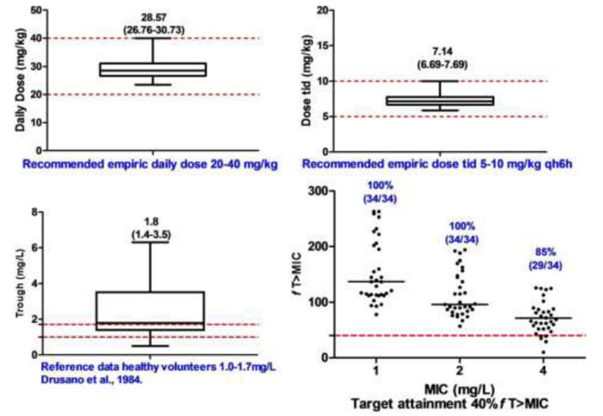

**Fig. 37 (Abstract P54).** Imipenem in septic burn patients (ICU): (**a**) Daily dose recommended; (**b**) Dose tid; (**c**) Trough obtained, means (CI95, min/max values). (**d**) Imipenem target attainment in burn patients at the recommended empiric dose regimen 2g daily (0.5g qh6). Medians, Mann Whitney test, Prism v.5

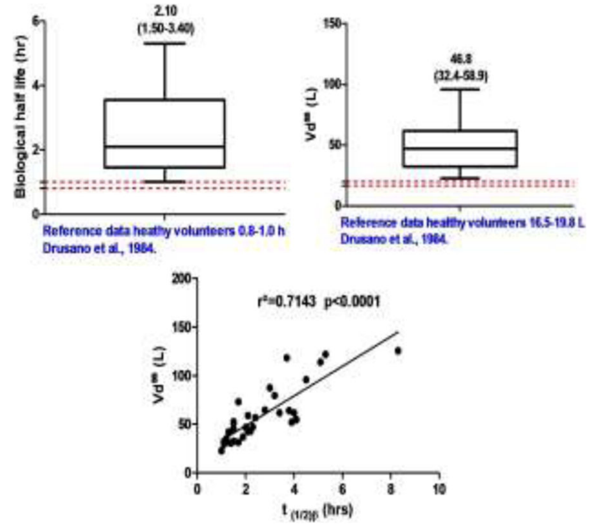

**Fig. 38 (Abstract P54).** Pharmacokinetics of Imipenem, medians (quartiles, min/max values). Abbreviations: **a**) $t_{(1/2)\beta}$: biological half life, **b**) $Vd^{ss}$: volume of distribution, **c**) Linear correlation $Vd^{ss}$ *versus* $t_{(1/2)\beta}$. Statistics: Mann Whitney test, Prism v.5

### P55

### Piperacillin-vancomycin effectiveness by PK/PD approach in septic burn patients with renal failure receiving the empiric dose regimen recommended

Vanessa Kasubeck[1], João Manoel da Silva[2], Edvaldo V. Campos[2], Carlindo Vieira da Silva Jr[1], Silvia R. C. J. Santos[1], David S. Gomez[2]
[1]FCF USP - Pharmacokinetics Center, School of Pharmaceutical Sciences (Sao Paulo/SP/Brazil); [2]HCFMUSP - Division of Plastic Surgery and Burns (Sao Paulo/SP/Brazil)
*Critical Care* 2017, **21(Suppl 2):**P55

**Introduction:** Metabolic and changes in pharmacokinetics-pharmacodynamics (PK/PD) changes are expected in burn patients (ICU). In general, renal insufficiency occurs during septic shock; then, the control of infection is a challenge, once drug effectiveness depends on drug plasma levels during the antimicrobial therapy.
**Objective:** The aim was to investigate drug effectiveness in septic burn patients receiving vancomycin-piperacillin combined at the dose regimen recommended to renal dysfunction based on drug plasma monitoring and PK/PD approach.
**Methods:** The protocol was approved by ethical committee N. 0069/15. Ten adult burn patients of both genders (2 F/8 M) were investigated: 33 (31-42) years, medians (quartiles), 69 (60-80) kg, total burn surface area 43 (32-49) %; creatinine clearance was 31 (23-40) ml/min. The agent was fire, inhalation injury occurred in 6/10 patients. Tracheal intubation and vasoactive drugs were required in all patients; none was under haemodialysis. Antimicrobials were combined as follows according to dose recommended to renal dysfunction: vancomycin, 1 hour infusion (1 g once a day) and piperacillin-tazobactan 0.5 hr infusion (2.25 g qh6). Nosocomial pathogens isolated from haemoculture and MIC values were obtained by the susceptibility testing performed in the central laboratory. A series of four blood samples were collected (2 ml/each) for drug plasma measurements through liquid chromatography, and PK data was based on noncompartmental analysis. PK/PD approach was performed by Prism 5.0, based on predictive index recommended: AUC/MIC > 400 for vancomycin, 100%fT > MIC for piperacillin, MIC value is the minimum inhibitory concentration, in vitro data obtained. Percentage of target attainment (PTA) related to β-lactam agent was % fT > MIC that means the percentage of time dose interval that free drug plasma levels are above the MIC data for isolated strains.
**Results:** Pharmacokinetics was altered in a different manner for antimicrobials associated to burn patients with renal failure, Fig. 39. Vancomycin target was attained against isolated gram-positive pathogens, MIC < 2 mg/L (Streptococcus spp, MIC 1 mg/L, Staphylococcus aureus MSSA/MRSA, MIC 2 mg/L) at the recommended dose for patients with renal failure, Fig. 40. The target was reached to piperacillin empirical dose regimen for all patients against MIC < 8 mg/L, strains; while dose adjustment was required in 4/10 patients against MIC 16 mg/L, strains (Enterococcus faecalis, Pseudomonas aeruginosa). Cure was registered in all patients during the clinical course by eradication of pathogens.
**Conclusions:** Drug plasma monitoring and PK/PD approach are today considered clinically relevant tools for target attainment; cure of nosocomial infections and also to avoid microbial resistance in critically ill burn patients (ICU).

**Reference**

1. Gomes DM et al: Pharmacotherapy 2014; 34:663-669

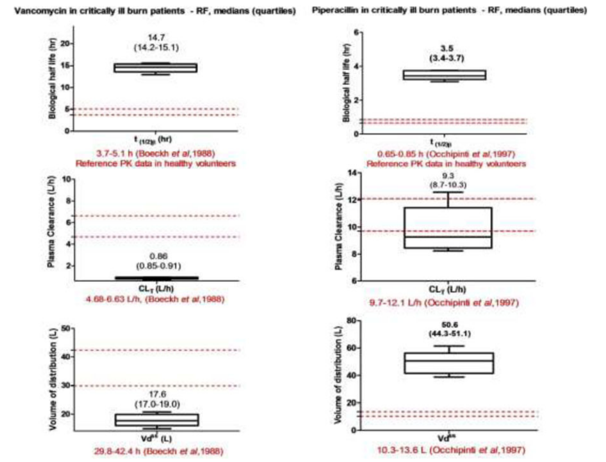

**Fig. 39 (Abstract P55).** Pharmacokinetics of Vancomycin and Piperacillin, medians (quartiles, min/max values). Abbreviations t(1/2)b biological half-life, Vdss volume of distribution, CLT plasma clearance. Statistics: Mann Whitney test, GraphPad Prism v. 5.0, significance p <0.05

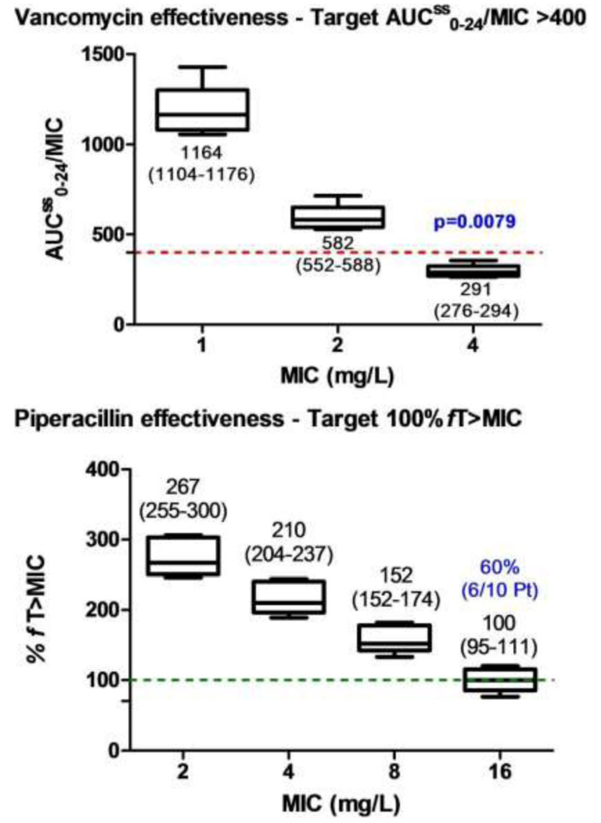

**Fig. 40 (Abstract P55).** Vancomycin and Piperacillin combined therapy in septic burns for drug effectiveness, medians (quartiles, min/max values). Abbreviations AUCss0-24: area under the plasma concentration time curve; fT > MIC: percentage of time dose interval that free drug plasma levels are above the MIC data

### P56

### Piperacillin effectiveness in septic burn patients by comparison of two empiric daily dose 12 versus 16 g against susceptible strains based on drug plasma measurements done in a real time

João Manoel da Silva[2], Amanda Maria Ribas Rosa de Oliveira[2], Carlindo Vieira da Silva Jr[1], Silvia R. C. J. Santos[1], David S. Gomez[2]
[1]FCF USP - Pharmacokinetics Center, School of Pharmaceutical Sciences (Sao Paulo/SP/Brazil); [2]HCFMUSP - Division of Plastic Surgery and Burns (Sao Paulo/SP/Brazil)
*Critical Care* 2017, **21(Suppl 2):**P56

**Introduction:** Metabolic changes occur in critically ill patients, and pharmacokinetics-pharmacodynamics (PK/PD) altered for antimicrobial agents is expected. Consequently, the control of severe infections in burn patients is a challenge, once drug effectiveness depends on antimicrobial plasma levels during the therapy.

**Objective:** To evaluate drug effectiveness for target attainment by PK/PD approach in burn patients receiving piperacillin-tazobactam 4.5 g tid by comparison of two empirical dose regimens.

**Methods:** 35 adult patients of both genders (6 F/29 M) and Clcr > 50 ml/min were included: 39+/-16 yrs (mean +/- SD), 70+/-12 kg, total burn surface area 33+/-20%. Thermal/electrical injuries occurred in 32/3 patients and inhalation injury in 21/35 of them. Tracheal intubation and vasoactive drugs were necessary in 30/35 patients. Length of stay in the Intensive Care Burn Unit was 21-37 days (95% CI). Empirical dose regimen recommended was prescribed to burn patients in septic shock caused by piperacillin susceptible nosocomial strains and daily dose 12 g versus 16 g were compared. Patients presented Clcr 115 (100 - 130) ml/min, white blood cells 11,601 (9,923 - 13,280) cells/mm3, C-reactive protein 202 (187- 255) mg/L, means (95% CI). Blood sampling was done for cultures, pathogens isolation and susceptibility testing done in the central laboratory. A series of 3-4 blood samples (2 mL/each) were done at time dose interval for drug plasma measurement done by liquid chromatography. Pharmacokinetics-pharmacodynamics (PK/PD) approach was performed by Prism 5.0, based on the predictive index of drug effectiveness (100%fT > MIC), and MIC is the minimum inhibitory concentration.

**Results:** It was shown a significant difference between piperacillin dose regimens, once the initial dose 4 g tid (4 g qh8 versus 4 g qh6) was compared: 173+/-29 (163-182) mg/kg versus 248+/-54 (230-265) mg/kg, $p < 0.0009$, means/SD (95% CI). In addition, trough was significant different 8.7 mg/L versus 20.2 mg/L, $p < 0.0001$ by comparison of doses 12 g/d and 16 g/d, respectively, Fig. 41. Piperacillin target was attained in septic burn patients only against isolated pathogens, MIC < 2 mg/L for 26/26 patients (100%), MIC 4 mg/L in 23/26 pat (88%), MIC 8 mg/L 14/26 pat (54%) at the recommended daily dose 4 g qh8; while PTA was reached daily dose 4 g qh6 for all patients up to MIC 16 mg/L, strains (Enterococcus faecalis, Pseudomonas aeruginosa). Cure was registered for all patients receiving 16 g daily (4 g qh6) during the clinical course by eradication of pathogens, Fig. 42. During the clinical course 12 deaths against 23 releases occurred.

**Conclusions:** Drug plasma monitoring done in real time based on PK/PD approach is quite useful permitting an earlier clinical intervention to treat the nosocomial infection in burn patients. Finally, these tools can impact the drug effectiveness related to avoid the bacterial resistance for more aggressive gram-positive and gram-negative piperacillin susceptible strains.

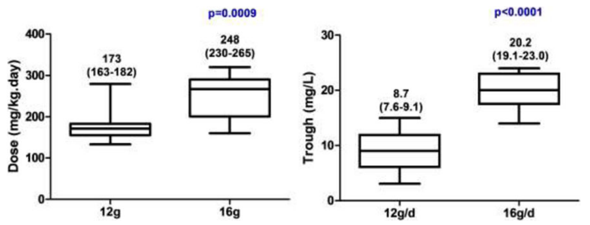

**Fig. 41 (Abstract P56).** Piperacillin initial dose recommended and trough obtained in septic burn patients (ICU), means (CI95). Abbreviations: CI confidence intervals. Statistics: T test, Prism v. 5.0, p <0.05

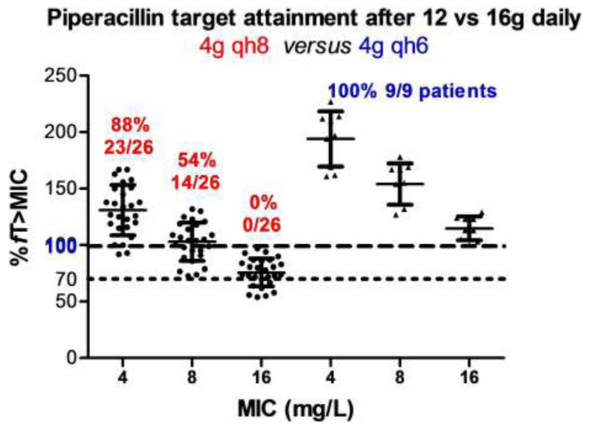

**Fig. 42 (Abstract P56).** Piperacillin target attainment in burn patients receiving the recommended empiric dose regimen 12 g versus 16 g daily (4 g tid). Abbreviations: fT > MIC: percent of time dose interval that free drug in plasma are above the MIC, MIC: minimum inhibi*tory concentration*

### P57

### Meropenem effectiveness in septic burn patients by comparison of extended infusion versus fast infusion against susceptible strains based on drug plasma measurements done in a real time

João Manoel da Silva Jr[2], Léonard da Vinci Kanda Kupa[1], Amanda Maria Ribas Rosa de Oliveira[2], Carlindo Vieira da Silva Jr[1], Silvia R. C. J. Santos[1], David S. Gomez[2]
[1]FCF USP - Pharmacokinetics Center, School of Pharmaceutical Sciences (Sao Paulo/SP/Brazil); [2]HCFMUSP - Division of Plastic Surgery and Burns (Sao Paulo/SP/Brazil)
*Critical Care* 2017, **21(Suppl 2):**P57

**Introduction:** Meropenem is a large spectrum carbapenem agent of high potency largely prescribed to critically ill patients in ICU against MIC < 4 mg/L, gram-positive and enteric gram-negative pathogens including Pseudomonas spp and MIC 8 mg/L, Enterococcus spp.
**Objective:** To evaluate drug effectiveness in burn patients (ICU) receiving recommended daily dose by comparison of extended (3 hrs) infusion with fast (0.5 hr) infusion by pharmacokinetics-pharmacodynamics (PK/PD) approach.
**Methods:** In spite of similar initial daily dose 41 versus 43 mg/kg (0.5 hr vs 3 hrs infusion) equivalent to 14.3 mg/kg tid in both groups of patients, medians (p = 0.6750, NS), it was shown significant difference between infusions by comparison of extended 3 hrs infusion with fast 0.5 hr infusion, once trough plasma levels 7.2 mg/L (3 hrs) were higher than 2.6 mg/L (0.5 hr), p = 0.0017, Fig. 43. Meropenem target after both infusions was attained in all patients against MIC < 4 mg/L, enteric bacteria and Pseudomonas aeruginosa. Concerning drug effectiveness MIC 8 mg/L strains, it was shown the superiority of extended infusion once the target was reached in these group of patients by comparison with target attainment only in 7/10 patients (0.5 hr infusion) against Enterococcus faecalis, Enterococcus faecium. Cure was registered for all patients receiving extended infusion during the clinical course by eradication of isolated pathogens, Fig. 44.
**Results:** In spite of similar initial daily dose 41 versus 43 mg/kg (0.5 hr vs 3 hrs infusion) equivalent to 14.3 mg/kg tid in both groups of patients, medians (p = 0.6750, NS), it was shown significant difference between infusions by comparison of extended 3 hrs infusion with fast 0.5 hr infusion, once trough plasma levels 7.2 mg/L (3 hrs) were higher than 2.6 mg/L (0.5 hr), p = 0.0017, (Fig. 43). Meropenem target after both infusions was attained in all patients against MIC < 4 mg/L, enteric bacteria and Pseudomonas aeruginosa. Concerning drug effectiveness MIC 8 mg/L strains, it was shown the superiority of extended infusion once the target was reached in these group of patients by comparison with target attainment only in 7/10 patients (0.5 hr infusion) against Enterococcus faecalis, Enterococcus faecium. Cure was registered for all patients receiving extended infusion during the clinical course by eradication of isolated pathogens, Fig. 44.
**Conclusions:** Drug plasma monitoring done in real time based on PK/PD approach showed to be important tool that can impact drug effectiveness and desired clinical outcome in critically ill septic patients.

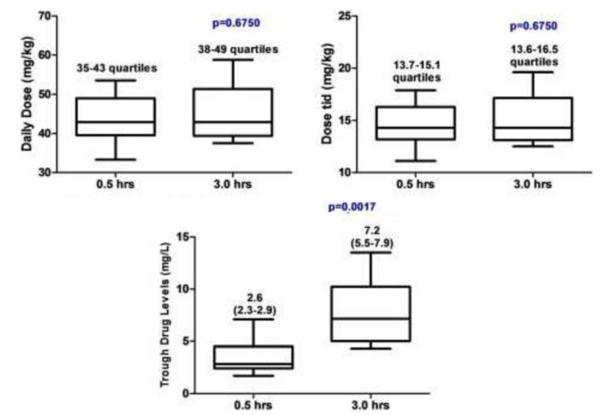

**Fig. 43 (Abstract P57).** Meropenem initial dose recommended and trough obtained in septic burn patients (ICU), medians (quartiles) by comparison of extended versus fast infusion. Abbreviations: CI confidence intervals. Statistics: Mann Whitney test, Prism v. 5.0, p < 0.0

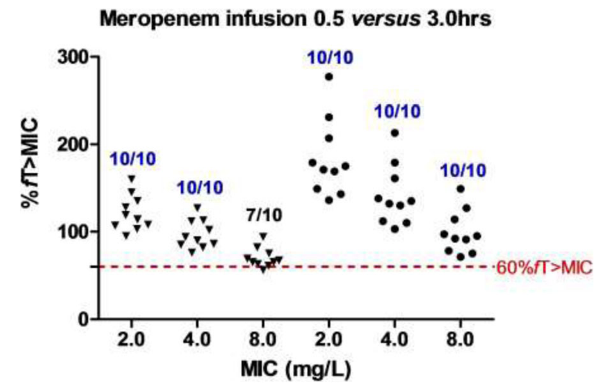

**Fig. 44 (Abstract P57).** Superiority of extended Meropenem infusion for target attainment in burn patients receiving the empiric dose Abbreviations: fT > MIC: percent of time dose interval that free drug in plasma are above the MIC

### P58

### Assessment of the adequacy of the Sepsis-III criteria for the diagnosis of sepsis in Brazil

Ian Ward Abdalla Maia[1,2], Mario Castro Alvarez Perez[1], Luís Filipe Silva Figueiredo[1,2], João Gouveia Lacerda Marinho[2]
[1]UNIFESO - Fundação Educacional Serra dos Órgãos (Teresópolis, RJ, Brasil); [2] HCPM - Hospital Central da Polícia Militar (Rio de Janeiro, RJ, Brasil)
*Critical Care* 2017, **21(Suppl 2):**P58

**Introduction:** In 2016, a new consensus on the definitions of sepsis (SEPSIS-III) was released amid major controversy. The new criteria endorse the SOFA score (Sequential Organ Failure Assessment) as the main diagnostic tool [1]. In Brazil, the main criticism for the application of these new criteria is the expected difficulty in obtaining, in a timely manner, the results of complementary exams that are components of the SOFA, which could delay the diagnosis and treatment of sepsis, worsening the patient's prognosis. Thus, based on data collected only in developed countries, the new approach seems to be a setback for countries where the health system is precarious and there is a clear need for studies that assess its real applicability in such scenarios.
**Objective:** Verify if the proposed new approach delays the diagnosis of septic patients and, thus, worsens their prognosis.
**Methods:** This research is a prospective observational study. A questionnaire was applied to 21 patients in the emergency room of a hospital in Rio de Janeiro, which allows to compare the "door-to-diagnostic" time, according to the usual approach versus that obtained through the approach proposed by SEPSIS-III. The ΔDoor (Systemic inflammatory response syndrome) and ΔDoor times were defined as the time elapsed from entry into the unit to the diagnosis of sepsis by the SIRS and SOFA criteria, respectively. These times were considered similar when the difference between them was less than 30 minutes.
**Results:** Among the analyzed cases, 95% had two or more SIRS criteria at the time of the initial evaluation, thus being diagnosed as septic, while only 71% had a SOFA greater than or equal to two. In patients diagnosed as septic, both by SIRS and SOFA criteria, it was observed that, in five cases, ΔDoor was equal to ΔDoor. In four cases, ΔDoor was higher, whereas in other four cases ΔDoor was lower (Fig. 45). As a whole, there were no significant differences in the time elapsed since the patient entered the unit until the diagnosis of sepsis was established based on SIRS or SOFA criteria. It is important to note that in 80% of patients with altered SOFA, the diagnosis was established based only on the clinical parameters belonging to the score and the arterial blood gas analysis as shown in Fig. 46.
**Conclusions:** It is clear that more advanced methods for the identification of sepsis in the emergency room are not needed. Instead, it is necessary to do a work of awareness, among the population and health professionals, so that we can start the treatment as soon as possible and, thus, guarantee a better prognosis for the septic patient.

**References**
1. Singer M, Deutschman CS, Seymour C, et al. The Third International Consensus Definitions for Sepsis and Septic Shock (Sepsis-3). JAMA. 2016; 315(8):801-810. doi:10.1001/jama.2016.0287
2. Angus DC, van der Poll T. Severe sepsis and septic shock. N Engl J Med. 2013;369(9):840-851.

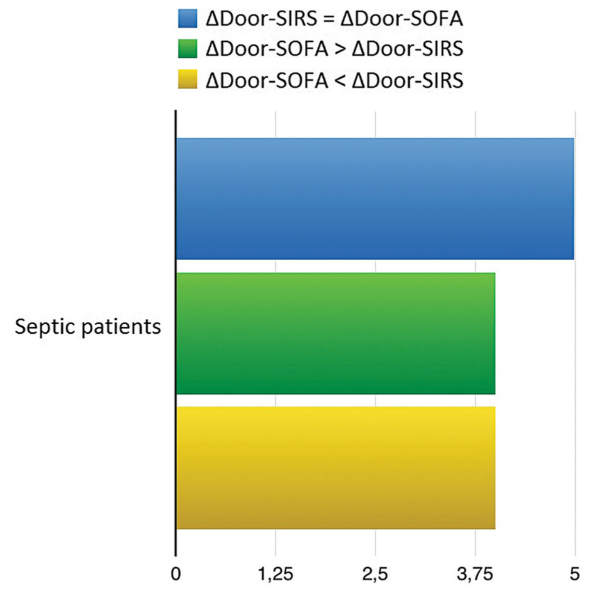

**Fig. 45 (Abstract P58).** Case distribution based on ΔDoor-SOFA and ΔDoor-SIRS times

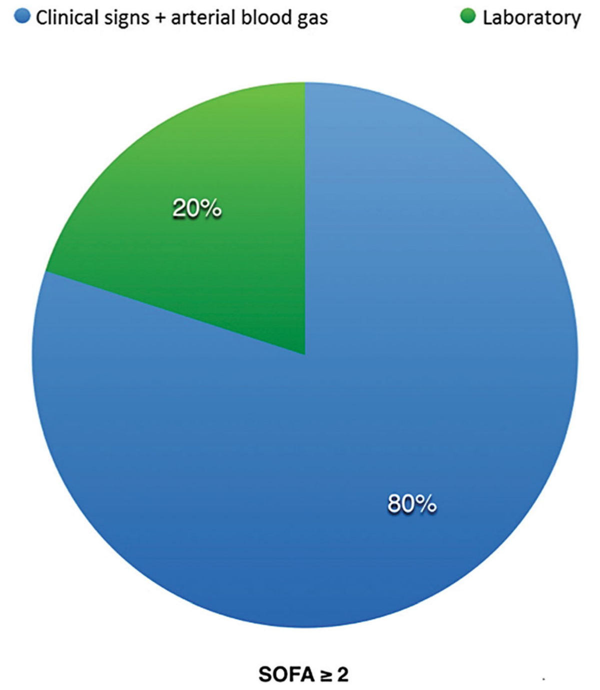

**Fig. 46 (Abstract P58).** Tools needed for the diagnosis of sepsis by SEPSIS-III

### P59

### Is qSOFA ≥ 2 the correct threshold for identifying patients at risk for sepsis?

Glauco Adriano Westphal, Míriam Cristine Machado, Flávia Ribeiro Machado, Maurício Gonçalves, Lucimeri Albino, Álvaro Koenig
Centro Hospitalar Un - Centro Hospitalar Unimed (Joinville (SC), Brazil)
*Critical Care* 2017, **21(Suppl 2):**P59

**Introduction:** A threshold of quick Sepsis-related Organ Failure Assessment (qSOFA) ≥ 2 identifies septic patients with high-risk of death outside the intensive care unit (ICU). However, there is no consensus as to whether the same threshold should be used for the identification of patients at risk of sepsis. In daily medical practice, our main challenge is not to identify who will die from sepsis, but to detect patients at risk of sepsis early, in order to interrupt the evolution to a situation of greater risk of death.
**Objective:** To identify the thresholds for both prediction of death in patients with suspected infection, and for the identification of patients at risk of sepsis.
**Methods:** We analyzed 18,724 hospital electronic records of non-ICU patients between 2013 and 2016. Suspected infection was defined with the same criteria used in Sepsis-3.0 definitions. Patients with sepsis risk were identified by the presence of any organ dysfunction, as stated by the Surviving Sepsis Campaign about the Sepsis-3.0. The area under the ROC curve (AUROC) was used not only to identify thresholds with predictive capacity for death, but also for the early detection of patients at risk of sepsis.
**Results:** 5,715 non-ICU patients with suspected infection. From them, 353 (6.2%) died, and sepsis risk was observed in 3,394 (59.4%) patients. The best qSOFA threshold for mortality prediction was ≥ 2, with an AUROC = 0.71, Sensitivity = 53.8 and Specificity = 83.7). On the other hand, the best qSOFA threshold for identifying patients at risk of sepsis was ≥ 1, with an AUROC = 0.76, Sensitivity = 86.1 and Specificity = 52.5.
**Conclusions:** Distinct thresholds of qSOFA were identified for predicting death ( ≥2) and for early detection of patients at risk of sepsis ( ≥1). Hence, a single clinically detectable organic dysfunction is sufficient to establish the risk of sepsis.

### P60

### Simplified NEWS - Combining the specificity of qSOFA and SIRS sensitivity for detection of patients at risk of sepsis

Glauco Adrieno Westphal, Míriam Cristine Machado, Flávia Ribeiro Machado, Maurício Gonçalves, Lucimeri Albino, Álvaro Koenig
Centro Hospitalar Un - Centro Hospitalar Unimed (Joinville (SC), Brazil)
*Critical Care* 2017, **21(Suppl 2):**P60

**Introduction:** qSOFA appears to be too specific, whereas SIRS is quite sensitive. Combining the specificity of qSOFA with the sensitivity of SIRS signals could theoretically generate a tool for detecting patients at risk of sepsis.
**Objective:** To evaluate the accuracy of a simplified score based on the combination of qSOFA and SIRS signals for prediction of death and identification of patients with suspected infection and at risk of sepsis, comparing it to other commonly used early warning scores.
**Methods:** We analyzed 18,724 hospital electronic records of non-ICU patients between 2013 and 2016. Suspected infection was defined with the same criteria used in Sepsis-3.0 definitions. Patients with sepsis risk were identified by the presence of any organ dysfunction, as stated by the Surviving Sepsis Campaign about the Sepsis-3.0. The combination of the clinically detectable variables of qSOFA and SIRS composed a simplified score with the majority of variables that make up the known National Early Warning Score (NEWS). The simplified NEWS (S-NEWS) was generated after attribution of weights according to the odds ratio for death of each of the following variables: temperature > 38.5oC or < 36oC (weight 1), heart rate > 90 bpm (weight 1), systolic arterial pressure ≤100 mm Hg (weight 1), brain dysfunction (weight 2), respiratory rate ≥22 mpm (weight 2) and O2 supplementation (weight 2). The area under the ROC curves (AUROC) were used to identify the prediction ability of death and risk of sepsis of the following prediction scores: S-NEWS, NEWS, Modified Early Warning Score (MEWS), quick SOFA (qSOFA) and Systemic Inflammatory Response Syndrome (SIRS).
**Results:** From 5,715 non-ICU patients with suspected infection, 353 (6.2%) died, and sepsis risk was observed in 3,394 (59.4%) patients. The accuracy for mortality prediction were the following for NEWS ≥8 (AUROC = 0.78; Sens = 64.8, Spec = 76.9), MEWS ≥4 (AUROC = 0.73, P < 0.001; Sens = 57.2; Spec = 78.5), qSOFA ≥2: (AUROC = 0.71; Sens = 53.8; Spec = 83.7), SIRS ≥2: (AUROC = 0.71; Sens = 87.2, Spec = 17.1) and S-NEWS ≥3: (AUROC = 0.70; Sens = 63.4; Spec = 64.2). The highest accuracy for sepsis risk identification was from S-NEWS ≥2: (AUROC = 0.87; Sens = 81.5; Spec = 79.0), followed by: NEWS ≥5 (AUROC = 0.83; Sens = 82.8, Spec = 65.7), MEWS ≥2 (AUROC = 0.78; Sens = 75.4; Spec = 70.0), qSOFA ≥1: (AUROC = 0.76; Sens = 86.1; Spec = 52.5), SIRS ≥2: (AUROC = 0.69; Sens = 77.7, Spec = 55.4).
**Conclusions:** S-NEWS presented the best ability to identify sepsis among patients with suspected infection but not for identifying risk of death. MEWS and NEWS were more accurate than qSOFA for predicting death in non-ICU patients with suspected infection.

### Surgery/Trauma

### P61

### Propofol free plasma level correlates with increases on hypnotic effect in patients undergoing CABG-hypothermic cardiopulmonary bypass by PK/PD approach through a new bioanalytical chromatographic method

Carlos Roberto da Silva Filho[1], Carlindo Vieira da Silva Jr[1], Maria José Carvalho Carmona[2], David S. Gomez[2], Silvia R. C. J. Santos[1]
[1] FCF USP - Pharmacokinetics Center, School of Pharmaceutical Sciences (Sao Paulo/SP/Brazil), [2]HCFMUSP - Division of Plastic Surgery and Burns (Sao Paulo/SP/Brazil)
*Critical Care* 2017, **21(Suppl 2):**P61

**Introduction:** During coronary artery bypass grafting (CABG) under hypothermic cardiopulmonary bypass (CPB-H) profound changes occur on propofol effect, once drug plasma binding could be altered in these patients.
**Objective:** A new chromatographic bioanalytical method was validated to investigate free propofol plasma levels in patients undergoing CABG under hypothermic cardiopulmonary bypass by applying the pharmacokinetics-pharmacodynamics (PK/PD) approach.
**Methods:** Ten patients (9 M/1 F) were investigated, medians: 62 yrs; 75 kg; 28 kg/m2. General anesthesia included sufentanil (500 mg.kg-1.h-1), pancuronium (0.1 mg/kg) and propofol target controlled infusion to achieve a predicted plasma concentration of 2.0 mg/l during CABG and 1.0 mg/l after the end of surgical procedure standardized in the hospital. Brain activity was continuously monitored by a BIS (bispectral index) device from the admission until the end of surgery, and extended to a period of 12 hrs. Blood sampling (3 ml/each) were done for drug plasma measurements by liquid chromatography (LC). PK/PD approach was done to correlate free propofol plasma levels versus BIS. Developed bioanalytical method was validated and free drug plasma level was measured by LC10 fluorescence detector RF10AXL (276/310 nm) Shimazdu (Kyoto, Japan) after the purification of biological matrix by ultrafiltration technique Amicon Ultra Millipore Ireland Ltd to quantify bonded and free propofol plasma levels. Only 0.5 ml of plasma was required by assay and thymol was chosen as internal standard. Daily curve was prepared based on eight calibrators added to a tube with internal standard. Chromatographic conditions were reversed phase column ShimPack CLC – ODS C18 150 x 6.0 mm, 5 microns Shimazdu and a binary mobile phase that consisted of a mixture of acetonitrile and water-acetic acid acidified to pH 4.6 (60:40, v/v). Mobile phase was prepared daily and degassed under helium (99.9%) strain and pumped isocratically at 0.8 ml/min, and each run time was 25 min.
**Results:** Bioanalytical method was adequate for propofol plasma measurements, (Fig. 47). Free drug plasma levels were obtained by difference of bounded from total propofol. Free propofol plasma levels were monitored during CABG, and data obtained at the post CPB-H were compared to the period before starts the CPB-H. Total drug binding to plasma proteins was reduced; then, increases in propofol free plasma levels occurred, Fig. 48. It was shown a strong correlation between BIS values and free drug levels by the chosen Emax model sigmoid shape-variable slope analyzed by Prism v.5, Fig. 48.

**Conclusions:** It was demonstrated that free fraction of propofol was increased by CPB-H during CABG intervention. A high correlation of BIS versus free drug plasma levels was obtained. PK/PD approach indicated that the free propofol plasma levels increased and the prolonged period of orotracheal-intubation could justify the residual hypnosis that occurs in these patients.

| Parameters – Bioanalytical method validated | Propofol |
|---|---|
| Linearity (lower and upper limits) | 10 - 10000 ng/ml |
| Coefficient of linear regression | 0.9977 |
| Limit of detection | 5 ng/ml |
| Total recovery (analyte) | 95.0% |
| Relative recovery to internal standard (thymol) | 99.9% |
| Intra-day precision (CV%) n=18 | 7.9% |
| Inter-day precision (CV%) n=27 | 8.9% |
| Systematic error intra-day, n=18 | 7.6% |
| Systematic error inter-day, n=27 | 8.1% |
| Stability of standard solution (bench, 4 hrs) n=9 | 3.9% |
| Stability of internal standard solution (bench, 4 hrs) n=6 | 4.5% |
| Short stability of samples (bench, 6 hrs) n=9 | 4.9% |
| Post-process stability (rack autosampler 24 hrs) n=9 | 5.7% |
| Long stability of samples (ultralow freezer) n=118 | 10% |
| Cycles freezing/de-freezing stability (n=27) | 6.8% |
| Robusteness (chromatographic system) n=72 | 8.9% |

**Fig. 47 (Abstract P61).** Confidence limits of bioanalytical method applied to propofol plasma measurements by liquid chromatography

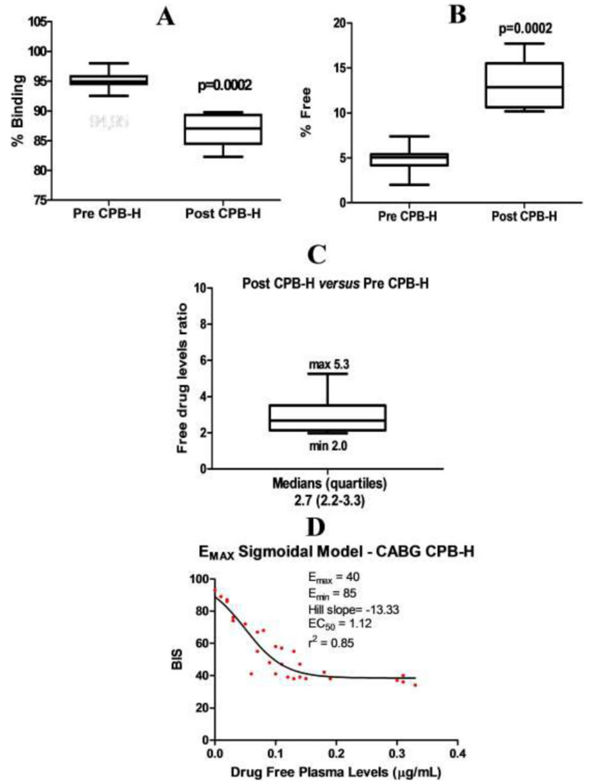

**Fig. 48 (Abstract P61).** Protein drug binding at the post versus pre-CPB-H. (**a**) Propofol bounded. (**b**) Propofol free. (**c**) Free plasma ratio (D) PK/PD model Emax

### P62

### Trauma patients' functional outcomes at discharge

Raquel A.C. Eid, José A.D.S Junior, Blenda N.G.M. Caldeira, Milton Steinman, Karina T. Timenetsky
HIAE - Critically Ill Department,Hospital Israelita Albert Einstein (São Paulo, SP, Brazil)
*Critical Care* 2017, **21(Suppl 2):**P62

**Introduction:** Trauma patients due to its severity tends to decrease functionality during hospital stay. Physiotherapy during hospital stay may minimize this impact. But how these patients return home after hospital discharge regards to functionality?
**Objective:** To evaluate trauma patient's functionality at hospital discharge.
**Methods:** A retrospective study, through medical chart and a specific prospective database of trauma patients admitted to a private hospital. All patients admitted with trauma diagnosis in the hospital, and that had information regards to functionality were included. Demographic data, trauma diagnosis, patients' severity (through SAPS), mechanical ventilation time, length of hospital stay and functionality were collected. We classified functionality in 5 categories: totally independent, modified independence (patient is independent but needs help with a specific device as walking stick, walker, etc), 25% functionality dependence, 50% functionality dependence, and total dependence.
**Results:** 26 trauma patients were admitted to the hospital in 2015 and 2016. Of these patients 42% were polytrauma patients, followed by 27% with polytrauma and brain injury associated, 11.5% firearm injury, 11.5% blunt trauma, 3.8% stab wound, 3.8% brain injury. Most patients were male (77%), with median age of 42 years old (range of 13-64), mean SAPS of 26 (±4,2), median mechanical ventilation time of 15 (range of 4-26), median length of hospital stay of 4 days (range of 1-48), most patients were sent home after hospital discharge (96%). At hospital admission, 11.5% of patients had independent functionality, 30% had 25% functionality dependence, 50% of patients had 50% functionality dependence, and 7.7% had total dependence. At hospital discharge most patients had modified independent functionality (34%), 30% were totally independent, 19.2% had 25% functionality dependence, 15.4% had 50% functionality dependence, and no patients were discharge from the hospital totally dependent, showing an improvement of patients' functionality. There was no correlation of functionality with patients' severity or hospital length of stay.
**Conclusions:** At hospital discharge there was an improvement in trauma patients' functionality, most patients with modified independent functionality. But some patients are still dependent in some degree, they would probably benefit from post hospital rehabilitation program.
Download to read this full article text

### Publisher's Note

Springer Nature remains neutral with regard to jurisdictional claims in published maps and institutional affiliations.

Submit your next manuscript to BioMed Central and we will help you at every step:

- We accept pre-submission inquiries
- Our selector tool helps you to find the most relevant journal
- We provide round the clock customer support
- Convenient online submission
- Thorough peer review
- Inclusion in PubMed and all major indexing services
- Maximum visibility for your research

Submit your manuscript at
www.biomedcentral.com/submit

BioMed Central
